# Long-term editing of brain circuits using an engineered electrical synapse

**DOI:** 10.1038/s41586-026-10501-y

**Published:** 2026-05-13

**Authors:** Elizabeth Ransey, Gwenaëlle E. Thomas, Elias M. Wisdom, Agustin Almoril-Porras, Ryan Bowman, Elise Adamson, Kathryn K. Walder-Christensen, Jesse A. White, Dalton N. Hughes, Hannah Schwennesen, Caly Ferguson, Kay M. Tye, Stephen D. Mague, Longgang Niu, Zhao-Wen Wang, Daniel Colón-Ramos, Rainbo Hultman, Nenad Bursac, Kafui Dzirasa

**Affiliations:** 1 Howard Hughes Medical Institute, Chevy Chase, MD USA; 2 Deparment of Psychiatry and Behavioral Sciences, Duke University Medical Center, Durham, NC USA; 3 Department of Neurobiology, Duke University Medical Center, Durham, NC USA; 4 Department of Neuroscience and Department of Cell Biology, Program in Cellular Neuroscience, Neurodegeneration and Repair, Yale University School of Medicine, New Haven, CT USA; 5 Department of Biomedical Engineering, Duke University, Durham, NC USA; 6 Salk Institute for Biological Studies, La Jolla, CA USA; 7 Department of Neuroscience, University of Connecticut School of Medicine, Farmington, CT USA; 8 Instituto de Neurobiología, Recinto de Ciencias Médicas, Universidad de Puerto Rico, San Juan, Puerto Rico; 9 Department of Molecular Physiology and Biophysics, Department of Psychiatry, University of Iowa, Iowa City, IA USA; 10 Department of Neurosurgery, Duke University Medical Center, Durham, NC USA

**Keywords:** Emotion, Molecular neuroscience

## Abstract

Electrical signalling across distinct populations of brain cells underpins cognitive and emotional function. However, approaches that selectively regulate electrical signalling between two cellular components of a mammalian neural circuit remain sparse. Here we engineered an electrical synapse composed of two connexin proteins^1^ found in *Morone americana* (white perch fish)—connexin 34.7 and connexin 35—to accomplish mammalian circuit modulation. By exploiting protein mutagenesis, devising a new in vitro system for assaying connexin hemichannel docking, and performing computational modelling of hemichannel interactions, we uncovered a structural motif that contributes to electrical synapse formation. Targeting this motif, we designed connexin 34.7 and connexin 35 hemichannels that dock with each other to form an electrical synapse but not with other major connexins expressed in the mammalian central nervous system. We validated this electrical synapse in vivo using worms (*Caenorhabditis elegans*) and mice (*Mus musculus*). We demonstrate that it can strengthen communication across neural circuits composed of pairs of distinct cell types and modify behaviour accordingly. Thus, we establish ‘long-term integration of circuits using connexins’ (LinCx) for precision circuit editing in mammals.

## Abstract (web summary)

Connexin proteins found in white perch fish were used to engineer synthetic electrical synapses, enabling precision circuit editing in mammals.

## Main

**Fig. 1:**
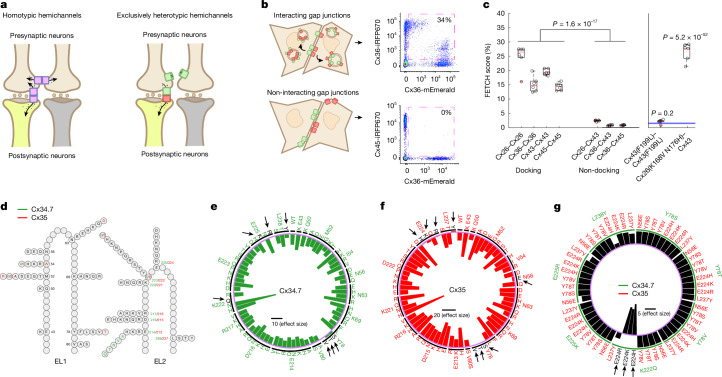
Screen to identify a mutant connexin hemichannel pair with exclusively heterotypic docking. **a**, Left, schematic outlining the limitation of introducing heterologous WT connexin hemichannels (pink rectangles) to modulate specific neural circuits composed of neurons (brown and yellow). Note that connexin hemichannels produce off-target electrical synapses between presynaptic neurons (left). Right, strategy for using exclusively heterotypic docking hemichannels (green and red rectangles) to selectively modulate specific neural circuits. **b**, Depiction of red (iRFP670) and green (mEmerald) fluorescence-exchange profiles (left) and representative flow cytometry plots (right) for hemichannel pairs with (Cx36–Cx36; top) and without (Cx36–Cx45; bottom) docking compatibility. The pink dashed squares in the flow cytometry plots highlight the proportion of cells that express two distinct fluorescent proteins. **c**, Left, proportion of dual fluorescence-labelled cells for connexin pairs with known docking compatibility profiles. Right, FETCH scores for Cx43(F199L)–Cx43(F199L) and Cx26(K168V N176H)–Cx43 (ref. ^26^). Blue lines on the right-hand graph are the mean ± s.e.m. score for the known-negative distribution of connexin pairs with docking incompatibility. **d**, Schematic of *M. americana* Cx34.7 and Cx35 mutations in EL1 and EL2 used to screen for heterotypic-exclusive hemichannels. Positions and mutations specific to Cx34.7 or Cx35 or common to both proteins, are shown in green, red and black, respectively. **e**,**f**, Plots showing homotypic FETCH results for Cx34.7 (**e**) and Cx35 (**f**) mutations. The locations of these mutations can be mapped back to the structure for EL2 in **d** by the mutation number and colour. Circular bar graphs show the Cohen’s *D* effect size of FETCH scores for homotypic mutant combinations compared with the heterotypic pairing of human Cx36 and Cx45, which fails to dock. The black horizontal line in the centre is the scale bar for effect sizes. Targeted residues are listed around the circle rim; substituted amino acids are listed in the interior. The intermittent black circle segregates each targeted residue, and the light purple circle corresponds to a Cohen’s *D* of zero. Mutations that disrupted docking are also highlighted by black arrows and letters. **g**, Heterotypic FETCH results for Cx34.7 and Cx35 mutant protein combinations. Bar graphs show the effect size of heterotypic mutant combinations relative to the WT Cx34.7 and Cx35 pair. The purple circle provides the reference point for an effect size of zero. The green intermittent circle corresponds to the Cx34.7 mutations identified by green labels in the outermost level around the rim of the plot. The black horizontal line in the centre is the scale bar for effect size. For *n* values and statistical tests, see main text. For definitions of box plots, see Methods.

Electrical synapses enable the direct flow of ions and small molecules between two cells and play a prominent part in coupling electrical activity in multiple organs, including the brain^2–4^. Electrical synapses comprise multiple gap junction channels, each composed of two docked hemichannels embedded in the membranes of two touching cells. Each hemichannel is an oligomer that consists of six monomeric proteins called connexins, of which there are 21 isoforms in humans^5,6^. Most connexins can form single-isoform hemichannels that dock with themselves to create homotypic gap junctions (Fig. 1a, left).

Neural circuit editing using gap junctions is well established in *C. elegans*^7–10^. *C. elegans* do not express connexins; thus, heterologous expression of the vertebrate connexin 36 (Cx36) across two connected *C. elegans* neurons results in the formation of an electrical synapse that does not interact with endogenous *C. elegans* gap junction proteins (innexins). Previous work has successfully implemented this editing approach to modify circuit physiology in multiple behavioural contexts, including *C. elegans* migration in response to various chemical and temperature conditions^8–11^.

The potential for using gap junctions to repair dysfunctional circuits has also been advanced in *C. elegans*, as shown in experiments that used circuit editing to restore normal behaviour in animals with induced circuit disruptions^11,12^. Nevertheless, this previous work highlighted a significant challenge in the use of gap junctions to edit select circuits in higher-complexity organisms. Specifically, when Cx36 was expressed in two sensory neurons of the same cell type and formed homotypic gap junctions, otherwise normal *C. elegans* showed disrupted behaviour in response to olfactory cues^11^. Because vertebrate brains are composed of many more cells of the same cell type than *C. elegans*, the ability of connexins to form homotypic gap junctions across more cells has the potential to substantially reduce the precision of this circuit-editing approach for mammals (for example, off-target modulation; Fig. 1a, left), which in turn produces greater behavioural disruption. Moreover, heterologous expression of connexins from other species in mammals might lead to gap junctions composed of both exogenous and endogenous connexins, thereby producing undesired connections that may impair neural circuit function.

Although nearly all connexins can form homotypic channels, several connexin isoforms can dock with other connexin isoforms to generate heterotypic channels^4,13^ (Fig. 1a, right). We reasoned that by identifying the mechanisms that underlie the docking interactions between connexins^14^, we could design a hemichannel pair biased towards heterotypic gap junction formation. We also reasoned that we could engineer this pair so that it is docking-incompatible with connexins endogenous to the mammalian CNS. This strategy can therefore produce a precise approach for regulating electrical flow between distinct cell types.

*M. americana* (white perch fish) expresses two homologues of mammalian neuronal Cx36—connexin 34.7 (Cx34.7) and connexin 35 (Cx35)—that create a heterotypic gap junction^1^. Notably, this electrical synapse exhibits channel-level rectification in the Cx34.7 to Cx35 direction when expressed in *Xenopus* oocytes^1^. The orthologues of Cx34.7 and Cx35 in the goldfish (*Carassius auratus*) CNS also create a heterotypic gap junction that shows circuit-level rectification in the same direction^15^. In summary, Cx34.7 and Cx35 can form heterotypic gap junctions with inherent directionality^15^ and can conduct currents capable of triggering action potentials^1^. Moreover, these connexins are potentially amenable to modification of biophysical properties through amino acid sequence mutations. On the basis of these findings, here we use Cx34.7 and Cx35 to engineer a new electrical synapse for editing mammalian circuits.

## In vitro assay of connexin hemichannel docking

To establish a method for evaluating connexin docking specificity and to ultimately engineer our electrical synapse, we leveraged the natural cellular turnover of docked connexin hemichannels. In mammalian cells, connexin hemichannels can be removed from the membrane through a coordinated endocytic and exocytic process that results in the internalization of fully docked gap junctions in double-bilayer vesicles called connexosomes^16–21^ (Fig. 1b, top left). By fusing connexin monomers with a fluorescent protein tag, internalization of labelled gap junctions from a fluorescent cell to a non-fluorescent cell can be visualized^21,22^ (Fig. 1b, top).

**Extended Data Fig. 1:**
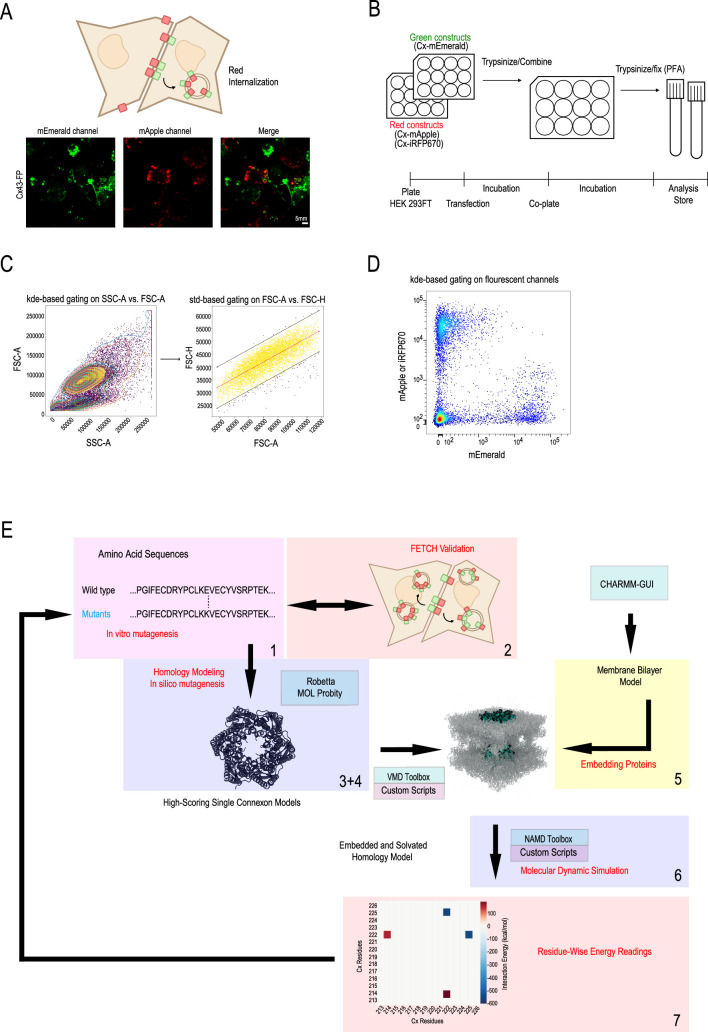
Schematic for Flow-Enabled Tracking of Connexosomes in HEK293FT Cells (FETCH) and integration with computation modeling. **A)** Top: Red fluorescence internalization following gap junction formation. Bottom: Co-plated population of cells expressing counterpart Cx43 tagged to mApple or mEmerald (42 Cx43/Cx43 samples subjected to the initial FETCH analysis). **B)** FETCH pipeline. **C)** Automated gating pipeline that uses sequential kernel density estimate (kde)- and standard deviation (std)-based gating approaches. **D)** Flow cytometry plot obtained from automated pipeline for a well containing one connexin pair. **E)** Integrated approach used to engineer Cx34.7 and Cx35 mutants with docking selectivity. Our approach consists of seven integrated components: 1) in vitro protein mutagenesis, 2) FETCH screening/validation, 3) homology model generation, 4) in silico protein mutagenesis, 5) embedding of proteins in a lipid bilayer and aqueous solution, 6) short-timescale energy minimization protocol incorporating explicit solvent and bilayer effects, 7) and residue-wise energy calculation.

Our approach used separate populations of HEK293FT cells that were transiently transfected with individual connexins as either mEmerald or iRFP670 fluorescent fusion proteins (Fig. 1b). We then co-plated and incubated the HEK293FT cells that expressed connexin counterparts. Finally, we evaluated their fluorescence exchange by flow cytometry (Extended Data Fig. 1a–d). As hemichannel docking is a prerequisite for internalizing fluorescently tagged connexins expressed by other cells, docking can be quantified as the proportion of transfected cells that are labelled by dual fluorescence in the co-plated sample (Fig. 1b,c). We first established the utility of our assay (termed flow-enabled tracking of connexosomes in HEK293FT cells (FETCH)) by testing well-characterized connexin isoforms: Cx26, Cx36, Cx43 and Cx45. Given that each of these is capable of homotypic docking^21–24^, we tested them under homotypic pairings (FETCH mean ± s.e.m. = 24.8 ± 1.8%, 15.2 ± 1.1%, 19.5 ± 0.4% and 14.4 ± 0.5% dual-labelled cells for Cx26–Cx26, Cx36–Cx36, Cx43–Cx43 and Cx45–Cx45, respectively; Fig. 1c, left). We also tested them in paired combinations for which there was previous evidence^13^ of heterotypic docking-incompatibility (for example, Cx26–Cx43, Cx36–Cx43 and Cx36–Cx45, which had FETCH values of 2.5 ± 0.1%, 0.8 ± 0.1% and 0.9 ± 0.1%, respectively; Fig. 1c, left). Notably, the proportion of dual-labelled cells in the population of docking-compatible versus docking-incompatible pairs was significantly different (*t*_40_ = 14.5, *P* = 1.6 × 10^−17^, two-tailed unpaired *t*-test). These results establish FETCH as a method that can be used to broadly assess connexin hemichannel docking compatibility.

Second, we analysed the utility of FETCH by testing two connexin mutations that affect gap junction formation. Specifically, we tested a Cx43(F199L) mutant that has previously been shown to disrupt trafficking to the cell membrane^25^. We also evaluated a Cx26(K168V N176H) mutant that confers heterotypic docking compatibility with wild-type (WT) Cx43 (ref. ^26^). In both cases, we tested whether the FETCH score for the mutants exceeded the scores for cell pairs under conditions in which docking was not anticipated (FETCH = 1.5 ± 0.2% for this ‘known negative’ distribution; *n* = 92 new cell pairs; Fig. 1c right, blue lines, and Methods). The homotypically paired Cx43(F199L) mutant did not exhibit a level of fluorescence exchange that was higher than the known-negative distribution (FETCH = 2.1 ± 0.3%, *t*_96_ = 0.85, *P* = 0.20, one-tailed unpaired *t*-test). By contrast, the Cx26(K168V N176H) mutant that heterotypically paired with Cx43 showed fluorescence exchange that was significantly increased (FETCH = 26.6 ± 1.3%, *t*_96_ = 30.9, *P* = 5.2 × 10^−52^, unpaired one-tailed *t*-test; Fig. 1c, right). Thus, we established that our FETCH assay can be used to identify connexin mutations that disrupt or enable docking compatibility.

## Cx34.7 and Cx35 mutant hemichannel docking

We used FETCH to assay a library of Cx34.7 and Cx35 mutants for their impact on hemichannel docking. Although the precise interactions that guide hemichannel docking are incompletely characterized for most connexins, structure–function and sequence analyses indicate that both the extracellular loops (EL1 and EL2) play a part in hemichannel docking^14,27–29^. To identify Cx34.7 and Cx35 variants that are unable to form homotypic gap junctions, we introduced around 70 individual mutations at 16 positions on both EL1 and EL2 of each connexin (Fig. 1d, Methods (for the design of the library of mutants) and Supplementary Fig. 1). We then compared the homotypic pairing FETCH scores of the mutants to a docking-incompatible heterotypic pair^30^ (for example, Cx36 paired with Cx45) (Fig. 1e,f). We identified several homotypic non-docking mutant proteins for Cx34.7 (Y78S, Y78T, Y78V, E225K, E225R, L238Y and K222Q) and Cx35 (N56E, Y78V, Y78S, Y78T, E224H, E224K, E224R and L237Y) (Fig. 1e,f and Supplementary Table 1). Next, to identify mutant pairs that exhibit exclusively heterotypic docking, we tested these Cx34.7 and Cx35 mutants against each other and compared their FETCH scores to WT Cx34.7–Cx35. We discovered three connexin mutant pairs for which FETCH scores were higher than those observed for Cx34.7(WT)–Cx35(WT) gap junctions. These results provide evidence of mutant pairs (Cx34.7(K222Q) with Cx35(E224H), Cx35(E224K) or Cx35(E224R)) that have intact heterotypic but reduced homotypic docking (Fig. 1g and Supplementary Table 1).

As our long-term objective was to develop a precise modulation approach that would be amenable for use in the mammalian nervous system, we also analysed whether the four identified mutant proteins could dock with the major connexins expressed by mammalian neurons and astrocytes, specifically Cx36 and Cx43, respectively^31,32^. For these analyses, we used FETCH and tested whether the scores for the mutant pairings were higher than that for the population of known-negative non-docking pair replicates (Methods).

None of the mutant proteins interacted with human Cx43: FETCH = 1.3 ± 0.1%, *t*_96_ = 0.29,* P* = 0.61 for Cx34.7(K222Q)–Cx43; FETCH = 0.4 ± 0.1%, *t*_96_ = 1.41, *P* = 0.92 for Cx35(E224H)–Cx43; FETCH = 0.5 ± 0.1%, *t*_96_ = 1.36, *P* = 0.91 for Cx35(E224K)–Cx43; and FETCH = 0.5 ± 0.1%, *t*_96_ = 1.28, *P *= 0.90 for Cx35(E224R)–Cx43 (one-tailed unpaired *t*-test; *n* = 6 replicates for all experimental connexin pairs). Cx35(E224K) and Cx35(E224R) also did not interact with human Cx36. However, Cx34.7(K222Q) and Cx35(E224H) formed heterotypic gap junctions with human Cx36: FETCH = 22.8 ± 1.9%, *t*_91_ = −24.4, *P* = 3.4 × 10^−43^ for Cx34.7(K222Q)–Cx36; FETCH = 5.9 ± 1.1%, *t*_96_ = −5.50, *P* = 1.6 × 10^−7^ for Cx35(E224H)–Cx36; FETCH = 0.8 ± 0.1%, *t*_96_ = 0.89, *P* = 0.81 for Cx35(E224K)–Cx36; and FETCH = 0.6 ± 0.1%, *t*_96_ = 1.24, *P* = 0.89 for Cx35(E224R)–Cx36 (one-tailed unpaired *t*-test). Thus, although Cx35(E224K) and Cx35(E224R) both showed docking incompatibility with Cx36 and Cx43, and neither showed homotypic docking, we did not identify an effective Cx34.7 partner that did not dock with Cx36 using single-point mutagenesis.

## Engineering a selective Cx34.7 and Cx35 pair

We used homology modelling and FETCH analysis to design a new Cx34.7 mutant that does not dock with endogenous Cx43 or Cx36, and to design its Cx35 heterotypic docking partner. In brief, we first developed computational models of WT and mutant Cx34.7 and Cx35 hemichannels under homotypic and heterotypic pairings. We then validated the computational model by comparing the key residues predicted to underlie hemichannel docking against the docking characteristics we measured for these mutants using FETCH. We also modelled their docking interactions with Cx36. Next, we used insights from all our residue-wise interaction models to computationally design Cx34.7 and Cx35 hemichannels that would dock heterotypically only with each other. Finally, we generated these proteins and confirmed their docking characteristics in vitro using FETCH (Extended Data Fig. 1e).

**Extended Data Fig. 2:**
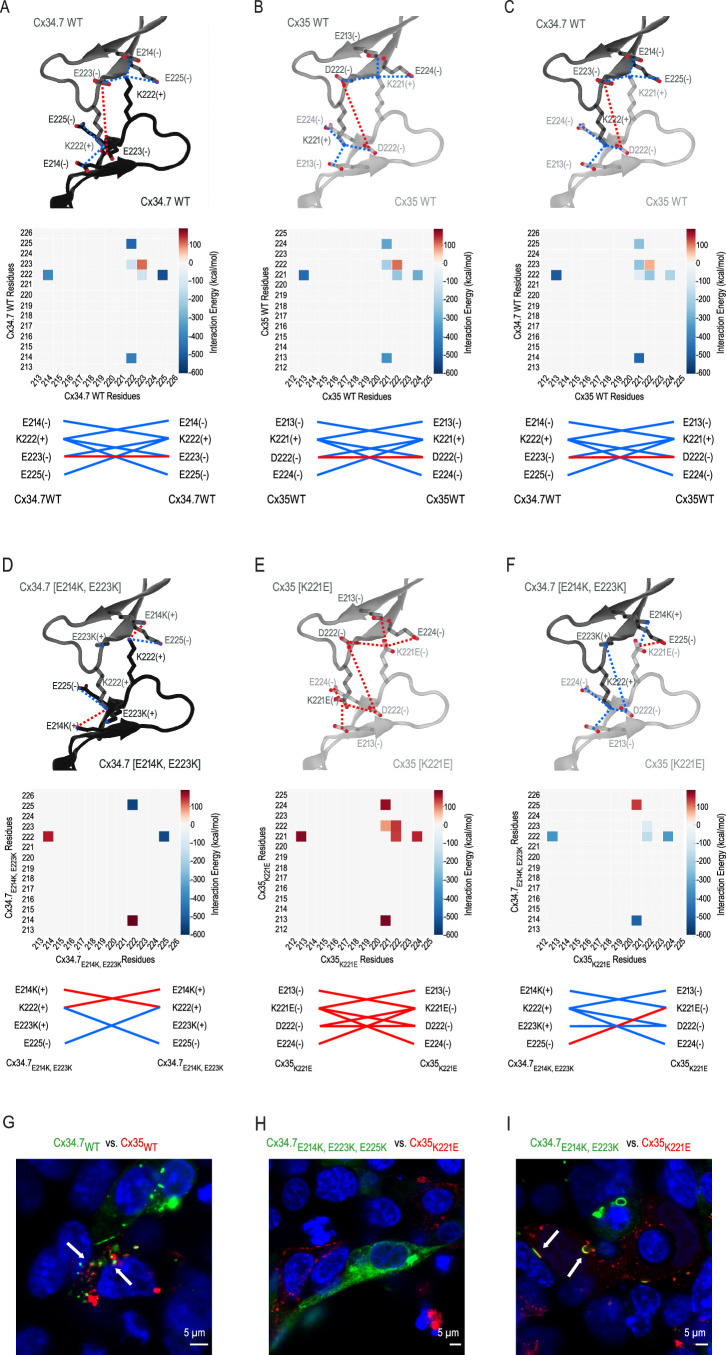
Engineering Cx34.7 and Cx35 mutants to show heterotypic, but not homotypic, hemichannel docking in mammalian cells. **A-C)** Predicted EL2-to-EL2 interactions among wild type Cx34.7 and Cx35 using homology modeling. Residues predicted to form strong attractive/repulsive interactions are highlighted in blue/red dashed lines, respectively (top). Contact plots for EL2-to-EL2 interactions produced by molecular dynamics simulation (middle), and summary of interactions predicted to stabilize hemichannels pairs (bottom). Blue in the middle and bottom rows indicates residues that show attractive interactions, and red indicates residues that show repulsive interactions. Plots are shown for **A)** homotypic Cx34.7, **B)** homotypic Cx35, and **C)** heterotypic Cx34.7 and Cx35 interactions. **D-F)** Homology models predicting EL2-to-EL2 residue interactions for Cx34.7 and Cx35 mutant hemichannels. The same color schemes from panels A-C apply here. Plots are shown for **D)** homotypic Cx34.7_E214K, E223K_, **E)** homotypic Cx35_K221E_, and **F)** heterotypic Cx34.7_E214K, E223K_ and Cx35_K221E_ interactions (Cx34.7 residues are along the y-axis and Cx35 residues are along the x-axis). **G-I)** Confocal images of heterotypic Cx pairs expressed in HEK293FT cells: G) Cx34.7_WT_/Cx35_WT_, **H)** Cx34.7_E214K, E223K, E225K_/Cx35_K221E_, and I) Cx34.7_E214K, E223K_/Cx35_K221E_. All Cx34.7 and Cx35 proteins are expressed as mEmerald and iRFP670 fusion proteins, respectively. White arrows highlight dual fluorescently labeled vesicles. Note the cytoplasmic localization of Cx34.7_E214K, E223K, E225K_ in panel H (images correspond to N = 49, 6, and 6 replicates subjected to FETCH analysis for Cx34.7_WT_/Cx35_WT_, Cx34.7_E214K, E223K, E225K_/Cx35_K221E_, and Cx34.7_E214K, E223K_/Cx35_K221E_, respectively).

First, to model the docking interactions between Cx34.7 and Cx35 hemichannels, we ran molecular dynamic simulations of homotypic and heterotypic pairs of WT and mutant Cx34.7 and Cx35 proteins^33,34^. We found large negative interaction energies involving residues E214, K222, E223 and E225 in WT Cx34.7 and residues E213, K221, D222 and E224 in WT Cx35 for both the homotypic and heterotypic docking simulations. These large negative interaction energies were suggestive of salt bridges that stabilize both homotypic and heterotypic docking interactions. This result was consistent with our FETCH screen, in which charge-swapping mutations (that is, changing a positive charge to a neutral and a negative charge to a positive at positions Cx34.7(K222) and Cx35(E224), respectively) disrupted docking. Integrating these results, we identified a common interaction motif for both Cx34.7 and Cx35 consisting of three negative residues (E214–E213, E223–D222 and E225–E224) and a positive residue (K222–K221) (Extended Data Fig. 2a–c). This interaction motif was consistent with a previously proposed theoretical framework in which four residues underlie the docking specificity of most connexin hemichannels^14^.

Next, we introduced Cx36 into our computational model. Both WT Cx34.7 and Cx35 showed strong interactions with Cx36, which paralleled the significant FETCH scores we observed (FETCH = 11.9 ± 1.2%, *t*_96_ = –12.93, *P* = 4.7 × 10^−23^ for Cx34.7–Cx36; FETCH = 18.0 ± 2.0%, *t*_96_ = –18.69, *P* = 4.7 × 10^−34^ for Cx35–Cx36, compared against the known-negative distribution, one-tailed unpaired *t*-test). We then modelled the four non-docking connexin mutants identified in our initial FETCH analysis (Cx34.7(K222Q), Cx35(E224H), Cx35(E224K) and Cx35(E224R)) against Cx36. Although the K222Q variant disrupted the large negative interaction energies we observed in the homotypic WT Cx34.7 model, the three remaining negative residues in the motif that contribute to docking compatibility in Cx34.7(K222Q) continued to show large negative interaction energies with the positive central lysine residue of Cx36. This result provides a potential mechanism for the heterotypic docking between Cx34.7(K222Q) and CX36 we observed via FETCH. By contrast, the three candidate Cx35 mutants we tested against Cx36 using FETCH (Cx35(E224H), Cx35(E224K) and Cx35(E224R)) maintained the positive K221 residue that formed strong interactions with the negative residues of Cx36. However, the Cx35(E224K) and Cx35(E224R) mutants induced strong repulsion with the positive K238 residue of Cx36. This finding provides insight into why these two mutants do not heterotypically dock with Cx36 in our FETCH analyses. Moreover, introducing a neutrally charged residue at the E224 position, as observed in the Cx35(E224H) mutant, was sufficient to restore the interaction with Cx36 in the computational model, which again mirrored the heterotypic docking profile we observed from our FETCH analyses.

**Extended Data Fig. 3:**
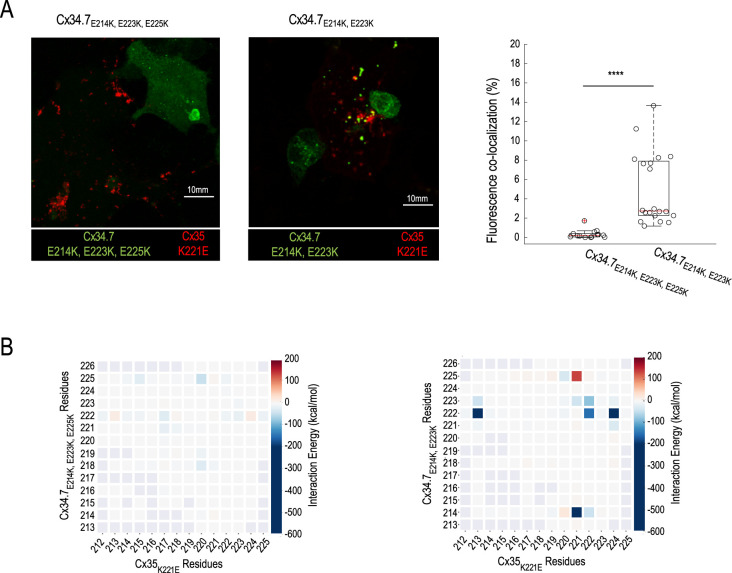
Cx34.7_E214K/E223K/E225K_ shows disrupted docking. **A)** 15 images were obtained of cell doublets of Cx34.7_E214K/E223K/E225K_ mutants (green) or Cx34.7_E214K/E223K/E225K_ mutants adjacent to Cx35 _K221E_ mutants (red). Each image obtained was a 5.5-micron Z-stack with 12 steps, which was then Z-projected to obtain a 3-D image for analysis. The Colocalization Threshold program on FIJI (ImageJ) was used to determine the percent of Cx34.7_E214K/E223K/E225K_ or Cx34.7_E214K/E223K_ pixels above the threshold that were colocalized to Cx35_K221E_ pixels. We observed a greater percentage of colocalized green and red pixels for the Cx34.7_E214K/E223K_/Cx35_K221E_ pairs than the Cx34.7_E214K/E223K/E225K_/Cx35_K221E_ pairs, indicating a higher rate of putative gap junction formation (U = 486, ****P < 10^−5^ across groups, two-tailed rank sum test). Images were obtained on the Zeiss 800 Confocal Microscope at North Carolina Central University Biomanufacturing Research Institute and Technology Enterprise facility. **B)** Contact plots for EL2-to-EL2 interactions produced by molecular dynamics simulation for Cx34.7_E214K/E223K/E225K_/Cx35_K221E_ (left) and Cx34.7_E214K/E223K_/Cx35_K221E_ (right, similar to Extended Data Fig. 3f).

Having modelled the putative interaction principles that underlie the docking specificity between Cx34.7, Cx35 and Cx36 and validated our models using FETCH, we set out to design a Cx34.7–Cx35 pair that would exhibit heterotypic docking only with each other. Our strategy was to mutate residues at the four positions of our identified docking motif such that one connexin isoform contained all negatively charged interactors (Cx35) and the other all positive (Cx34.7). Our Cx35 mutant Cx35(K221E) showed strong repulsions in our homotypic model (Extended Data Fig. 2e), did not exhibit homotypic docking, on the basis of FETCH analyses (FETCH = 1.2 ± 0.4%, *t*_96_ = 0.35, *P* = 0.64, one-tailed unpaired *t*-test) and did not dock with Cx36 or Cx43 (FETCH = 1.5 ± 0.1%, *t*_91_ = 0.02, *P* = 0.51 for Cx35(K221E)–Cx36; FETCH = 1.7 ± 0.2, *t*_96_ = –0.32, *P* = 0.37 for Cx35(K221E)–Cx43, one-tailed unpaired *t*-test). Similarly, the positively charged motif mutant Cx34.7(E214K E223K E225K) showed strong repulsions in our homotypic computational model and did not exhibit homotypic docking in FETCH analyses (FETCH = 0.2 ± 0.0%, *t*_96_ = 1.76, *P* = 0.96, one-tailed unpaired *t*-test). However, when we assayed Cx35(K221E) against Cx34.7(E214K E223K E225K) using FETCH, we did not observe significant fluorescence exchange (FETCH = 1.2 ± 0.3%, *t*_96_ = 0.37, *P* = 0.64, one-tailed unpaired *t*-test). Follow-up confocal imaging analyses of HEK293FT cells expressing the constructs revealed that Cx34.7(E214K E223K E225K) did not properly localize to the cell membrane (compare Extended Data Fig. 2g and Extended Data Fig. 2h; see also Extended Data Fig. 3). Moreover Cx34.7(E214K E223K E225K) was predicted to show weak attractive interactions with Cx35(K221E) in our heterotypic gap junction computational model (Extended Data Fig. 3). Thus, we evaluated an intermediate Cx34.7 mutant protein that exhibited positively charged residues at three out of the four critical interacting positions, Cx34.7(E214K E223K). This mutant showed repulsive interactions in our homotypic gap junction computational model (Extended Data Fig. 2d), and it showed strong attractive interactions with Cx35(K221E) (Extended Data Fig. 2f). This mutant docked with Cx35(K221E) as confirmed via FETCH analysis and confocal microscopy (FETCH = 35.7 ± 4.1%, *t*_96_ = 28.11, *P *= 2.0 × 10^−48^, one-tailed unpaired *t*-test; Extended Data Fig. 2i). Notably, Cx34.7(E214K E223K) did not show homotypic docking in our FETCH analysis (FETCH = 1.1 ± 0.2%, *t*_96_ = 0.46, *P* = 0.68, one-tailed unpaired *t*-test), nor did it dock with Cx36 or Cx43 (FETCH = 1.0 ± 0.2%, *t*_96_ = 0.58, *P* = 0.72 for Cx34.7(E214K E223K)–Cx36; FETCH = 0.9 ± 0.1%, *t*_96_ = 0.73, *P* = 0.77 for Cx34.7(E214K E223K)–Cx43, one-tailed unpaired *t*-test). Notably, the Cx34.7(E214K E223K) and Cx35(K221E) mutant pair showed a higher heterotypic FETCH score than CX36 under homotypic docking conditions (FETCH = 15.2 ± 1.1%) and the WT Cx34.7–Cx35 pair (FETCH = 12.0 ± 0.9%) as measured using our in vitro assay (*t*_10_ = 4.9, *P* = 6.4 × 10^−4^ for comparisons against Cx36–Cx36; *t*_10_ = 5.7, *P* = 1.9 × 10^−4^ for comparisons against WT Cx34.7–Cx35, two-tailed unpaired *t*-test at an *α* threshold adjusted by false discovery rate (FDR) correction for 2 comparisons; *n* = 6 replicates per group). From hereon, we refer to this connexin pair of Cx34.7(E214K E223K) and Cx35(K221E) as Cx34.7(M1)–Cx35(M1); that is, designer connexin version 1.0 from *M. americana*.

## Cx34.7(M1) and Cx35(M1) form a functional synapse

**Fig. 2:**
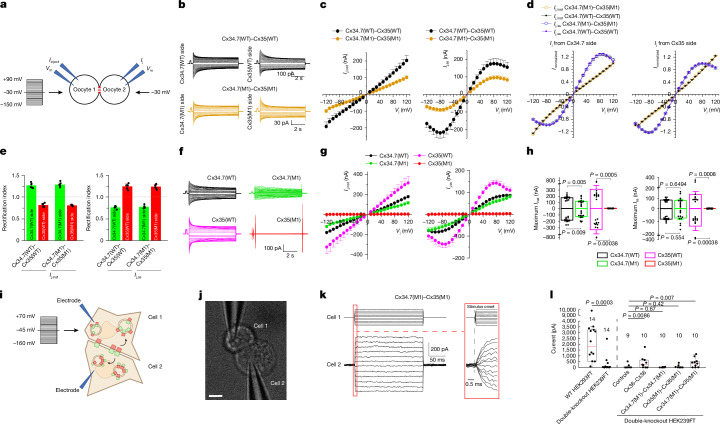
Biophysical properties of gap junctions formed by heterologous expression of WT and mutant Cx34.7 and Cx35 in *Xenopus* oocytes. **a**, Diagram showing the characterization of connexin gap junctions using oocyte pairs. The membrane voltage (*V*_m_) of oocyte 1 was stepped from a holding voltage of –30 mV to a series of voltages (−150 mV to +90 mV at 10-mV intervals), whereas that of oocyte 2 was held constant at −30 mV to record junctional currents (*I*_j_). **b**, Representative *I*_j_ traces from oocyte pairs expressing WT (Cx34.7(WT)–Cx35(WT), top) or mutant (Cx34.7(M1)–Cx35(M1), bottom) connexins. **c**, Relationships between instantaneous *I*_j_ (*I*_j,inst_) and transjunctional voltage (*V*_j_) (left), and between steady-state *I*_j_ (*I*_j__,__ss_) and *V*_j_ (right). *V*_j_ is defined as *V*_m_ of oocyte 2 – *V*_m_ of oocyte 1. **d**, Relationships between normalized *I*_j__,__inst_ and *V*_j_, and between normalized *I*_j,ss_ and *V*_j_. *I*_j_ was normalized to the peak current value of the side with the lower absolute magnitude. **e**, Comparison of *I*_j,inst_ and *I*_j,ss_ rectification indices. The rectification index is the ratio of the peak *I*_j_ recorded from the indicated side (for example, Cx34.7(WT) side or Cx35(WT) side) to the peak *I*_j_ from the opposing side. WT (Cx34.7(WT)–Cx35(WT)) and mutant (Cx34.7(M1)–Cx35(M1)) heterotypic gap junctions did not show significant differences in the rectification index. **f**, Representative *I*_j_ traces of homotypic gap junctions formed by Cx34.7(WT), Cx34.7(M1), Cx35(WT) and Cx35(M1). **g**, Relationships between *I*_j__,__inst_ and *V*_j_, and between *I*_j,__ss_ and *V*_j_ (*n* = 12 cell pairs per group). **h**, Comparison of the maximal *I*_j,inst_ and *I*_j,ss_ at positive and negative *V*_j_ among the different gap junctions. Bar graphs show the mean ± s.e.m. **i**, Schematic of the experimental setup for the functional characterization of connexin pairs. **j**, A representative HEK293FT cell pair from data presented in **l**. Scale bar, 10 μm. **k**, Schematic of recording for a Cx34.7(M1)–Cx35(M1) cell pair. Voltage steps (−160 mV to +70 mV at 10-mV intervals from a holding voltage of −45 mV) were applied to one cell (top), and the resulting *I*_j_ traces were recorded from the neighbouring cell (bottom) at an expanded time scale (only half of the voltage steps are depicted). **l**, Current at the maximum *V*_j_ for WT and connexin double-knockout HEK293FT cells and for double-knockout cell pairs transfected with Cx36, Cx34.7(M1) or Cx35(M1) under conditions of homotypic and heterotypic pairing. The number of cell pairs recorded is shown above individual boxes. For *n* values and statistical tests, see the main text. For definitions of box plots, see Methods.

To determine whether our mutant Cx34.7 and Cx35 hemichannels can form a functional electrical synapse, we used *Xenopus* oocytes as a heterologous expression system^35,36^. We also tested WT Cx34.7 and Cx35 hemichannels as controls. Connexins were expressed in separate populations of oocytes. Oocytes expressing either two different connexins or the same connexin were then paired to form heterotypic or homotypic gap junctions, respectively (Fig. 2a and Methods). In our analyses of heterotypic gap junctions, we detected junctional current (*I*_j_) in paired oocytes expressing Cx34.7(M1) and Cx35(M1) (Fig. 2b, bottom). As expected, we also detected current in pairs expressing the WT proteins (Fig. 2b, top). In response to symmetric transjunctional voltage (*V*_j_) steps (–120 mV to +120 mV), the mutant heterotypic gap junction exhibited significantly lower instantaneous *I*_j_ (*I*_j,inst_) and steady-state *I*_j_ (*I*_j,ss_) currents than the WT heterotypic gap junction (unpaired two tailed *t*-test, *n* = 6 per group; Fig. 2c). The *I*_j_ traces for mutant and WT pairs seemed to be asymmetric between the positive and negative *V*_j_ ranges, and the *I*_j_ traces recorded from one oocyte in a pair looked like a mirror image of those recorded from the other oocyte of the pair (Fig. 2b). As these findings were indicative of a rectification property of the mutant and WT gap junctions, we normalized the *I*_j,inst_ and *I*_j,ss_ and compared the rectification index (Fig. 2d,e). The *I*_j,ss_ was rectified in the Cx34.7 to Cx35 direction for the mutant and WT pairs, a result consistent with a report for the WT proteins^1^. By contrast, *I*_j,inst_ was rectified in the opposite direction for both pairs (unpaired two tailed *t*-test, *n* = 6 per group; Fig. 2e).

**Extended Data Fig. 4:**
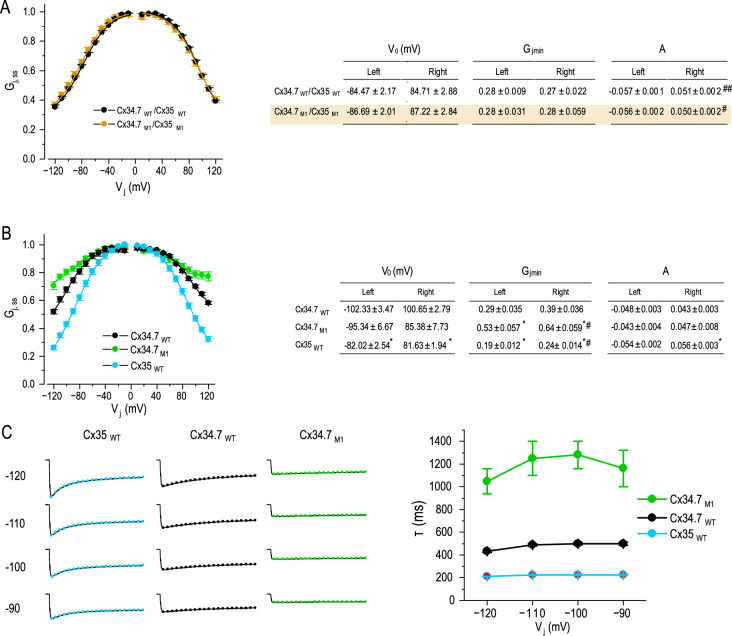
Biophysical properties of wild type and mutant Cx34.7 and Cx35 gap junctions. **A)** Relationships between steady-state junctional conductance (*G*_*j, ss*_) and transjunctional voltage (*V*_*j*_; left), and comparisons of *V*_*0*_, *G*_*jmin*_, *A*, for the left and right sides of the *G*_*j, ss*_ – *V*_*j*_ curves for Cx34.7_WT_/Cx35_WT_ vs Cx34.7_M1_/Cx35_M1_ junctions (right). These variables were quantified by fitting the Gj,ss – V_j_ curves to a Boltzmann function, where *V*_0_ is the voltage at which the conductance is half-maximal, *G*_min_ is the normalized voltage-insensitive residual conductance and *A* is a parameter defining the steepness of voltage sensitivity^98^. The *G*_*j, ss*_ – *V*_*j*_ plot on the left was based on averaged *I*_*j, ss*_ recorded from both oocytes of the pair, with *V*_*j*_ defined as *V*_*m*_ of Cx34.7_WT_ (or Cx34.7_M1_) *V*_*m*_ of Cx35_WT_ (or Cx35_M1_). For differences between the left and right *G*_*j, ss*_ – *V*_*j*_ curves, # - *p* < 0.05, ## - *p* < 0.01 (two-tailed paired *t*-test). No significant difference was detected between the two groups using a one-way ANOVA. N = 6 for all groups. **B)** Relationships between *G*_*ss*_ and *V*_*j*_ (left), and comparisons *of V*_*0*_, *G*_*jmin*_, and *A* values among the different gap junctions and between the left and right sides of the *G*_*j, ss*_ – *V*_*j*_ curves (right). * - compared with Cx34.7_WT_ (*p* < 0.01, one-way ANOVA followed by Tukey’s post-hoc test), # - differences between the left and right sides of the same *G*_*ss*_ – *V*_*j*_ curve (*p* < 0.01, two-tailed paired *t*-test). **C)** Representative *I*_*j*_ traces (solid lines) fitted by a single exponential (dotted lines), and comparison of the deactivation time constant (τ) among the different groups. N = 6 for all groups.

In our analyses of homotypic gap junctions, we detected *I*_j_ from oocytes expressing Cx34.7(WT), Cx35(WT) and Cx34.7(M1) but not Cx35(M1) (*n* = 12 per group; Fig. 2f). The homotypic gap junctions differed in *I*_j_ amplitude (unpaired two-tailed* t*-test; Fig. 2g,h), the relationship between the steady-state junctional conductance (*G*_ss_) and the *V*_j_ and the deactivation rate (Extended Data Fig. 4). Overall, these findings confirmed the formation of functional Cx34.7(M1)–Cx35(M1) gap junctions and the disruption of Cx35(M1) homotypic docking. These observations were consistent with our initial screening analysis using FETCH. Contradictory results were observed for Cx34.7(M1) under homotypic conditions, whereby we observed functional gap junctions using *Xenopus* oocytes but disrupted docking using FETCH in mammalian cells.

**Extended Data Fig. 5:**
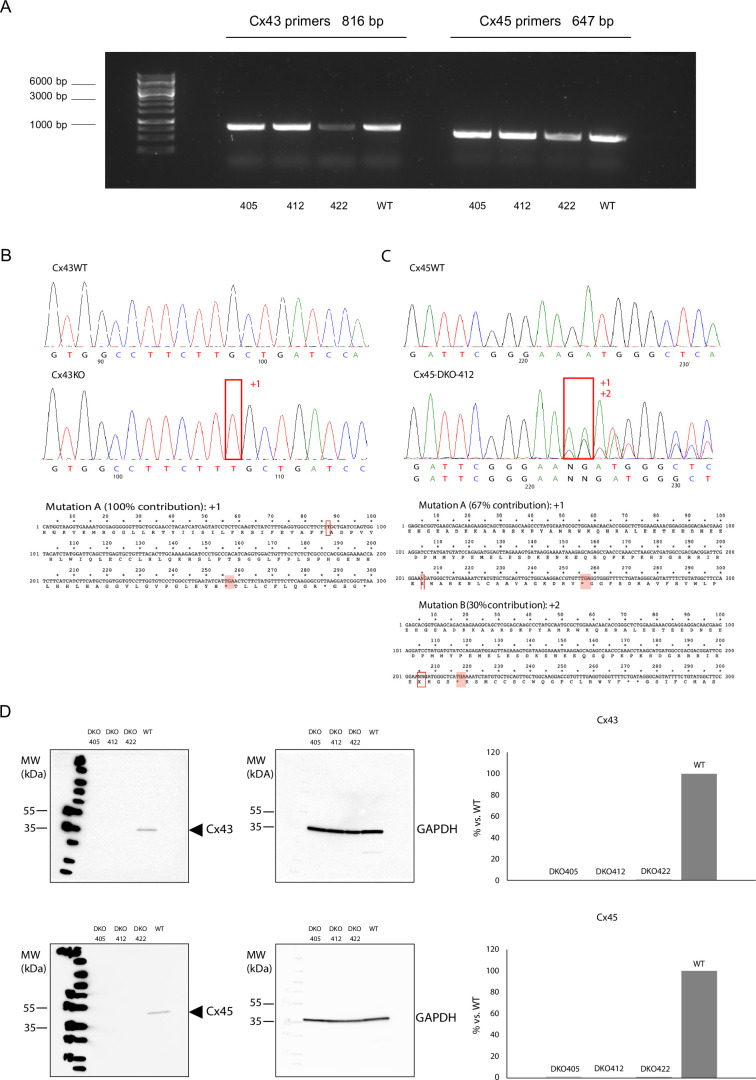
Cx34 and Cx45 double knockout HEK293FT cells. **A)** PCR of gDNA from the double knockout (DKO) HEK293FT cells showed one clean band indicating utility of the Cx43 and Cx45 primers (Cx43 expected amplicon size= 816 bp; Cx45 expected amplicon size=647 bp). Point mutations were introduced via CRISPR-Cas9 in the **B)** Cx43 and **C)** Cx45 sequences, adding respectively 1 specific nucleotide base (T) in Cx43 (Mutation A) and 2 specific nucleotide bases (AA) in Cx45 (Mutation B). Mutations are indicated by the red boxes. Those modifications induced a frameshift of the Cx43 and Cx45 DNA sequence. **D)** Western blotting analysis comparing three double knockout (DKO) HEK293FT cell lines to wild type (WT) HEK293FT cells. Note the absence of Cx43 (top row) and Cx45 bands (bottom row) in the DKO lines, but not in the WT cells. Specifically, Cx43 was decreased by 99.6%, 100%, and 99.4% in the DKO 405, 412 and 422 lines, respectively (top right); Cx45 was decreased by 98.5%, 100%, and 99% in the DKO 405, 412 and 422 lines, respectively (bottom right). Values were determined based on Cx43 or Cx45 / GAPDH. The DKO412 cell line was used for our subsequent recording analysis since it showed the strongest reduction of Cx43 and Cx45.

**Extended Data Fig. 6:**
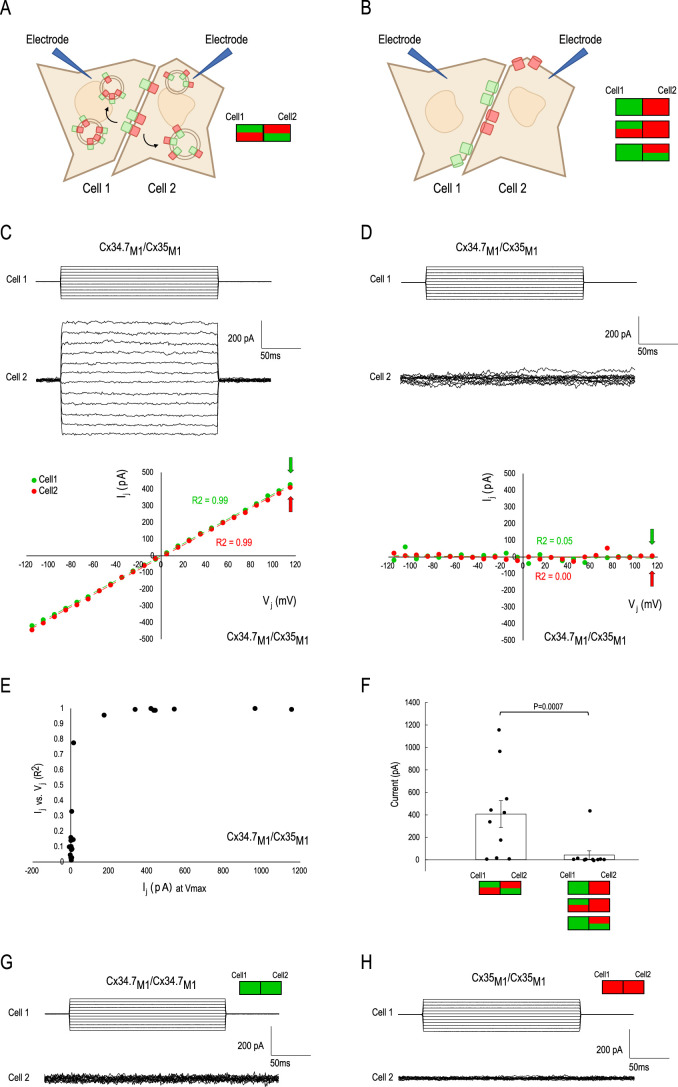
Functional validation of Cx34.7M1-mEmerald and Cx35M1-mApple using Cx43/Cx45 double knockout HEK293FT cells. **A)** We performed dual patch clamp recordings in Cx34.7_M1_-mEmerald and Cx35_M1_-mApple expressing cell pairs that were both labeled by red and green fluorophores, and **B)** fluorescently labeled HEK293FT double knockout pairs for which one of the cells was not labeled by both fluorophores. **C)** Representative injected and recorded current traces (top and middle), and the relationship between transjunctional current (*I*_*j*_) and transjunctional voltage (*V*_*j*_, bottom) for a cell pair with dual fluorescence internalization (same as Fig. 2i) and **D**) a cell pair where one cell was labeled by only one fluorescent protein (Pearson correlation values shown for each cell within the pair). **E)** Current at the max transjunctional voltage vs. relationship between current flow and transjunctional voltage for each cell pair (i.e., electrical coupling). A current > 100pA at the max transjunctional voltage was indicative of electrical coupling of the cell pair. **F)** The max transjunctional voltage current was higher for cell pairs that showed dual fluorescence labeling compared to pairs that did not (U = 75, P = 0.0007, one-tailed Wilcoxon rank sum test). **G-H)** Representative current traces for cell pairs that expressed **G)** Cx34.7_M1_ or **H)** Cx35_M1_ in a homotypic configuration. Injected and recorded currents are shown above and below, respectively. All current traces were filtered using Clampfit 11.4. They are depicted at a 20 mV step and 1 ms resolution.

To address these conflicting findings observed for Cx34.7(M1) under homotypic conditions, we next tested whether Cx34.7(M1) can form a functional homotypic electrical synapse in HEK293FT cells and thereby increase their electrical coupling (experimental setup for the functional characterization of connexin hemichannel pairs in HEK293FT cells is shown in Fig. 2i). Here we used CRISPR–Cas9 to generate a new HEK293FT cell line for which expression of the endogenous Cx43 and Cx45 proteins was disrupted (connexin double-knockout HEK293FT cells; Methods and Extended Data Fig. 5). This approach reduces the number of electrical synapses that naturally form between HEK293FT cells^37^ (Fig. 2l, left), which enabled us to assess whether functional connectivity is increased by Cx34.7(M1) expression. Heterotypic expression of Cx34.7(M1) and Cx35(M1) increased electrical connectivity between pairs of connexin double-knockout HEK293FT cells (*H*_4,48_ = 17.45, *P* = 0.0016, Kruskal–Wallis test; *U* = 60 and *P* = 0.007 compared with non-transfected connexin double-knockout cell pairs for post hoc analysis, one-tailed Wilcoxon rank-sum test at an *α* threshold adjusted by FDR for 4 comparisons; Fig. 2j–l and Extended Data Fig. 6). By contrast, neither homotypic expression of Cx34.7(M1) nor Cx35(M1) increased connectivity (*U* = 95 and *P* = 0.67 for Cx34.7(M1), and *U* = 87 and *P* = 0.42 for Cx35(M1) for post hoc comparisons with non-transfected connexin double-knockout cells, one-tailed Wilcoxon rank-sum test at an *α* threshold adjusted by FDR for 4 comparisons; Fig. 2l). Thus, in addition to finding that Cx34.7(M1) does not dock in a homotypic configuration in our FETCH analysis, Cx34.7(M1) does not form a functional homotypic electrical synapse when expressed in a mammalian cell line.

## Cx34.7(M1)–Cx35(M1) alters function and behaviour

Next, we set out to determine the in vivo docking selectivity and functionality of our Cx34.7(M1) and Cx35(M1) hemichannels by testing whether distinct hemichannel pairs can regulate the activity of two neurons that constitute a circuit and their output behaviour. Specifically, we evaluated hemichannels under homotypic and heterotypic conditions against the other major connexin proteins expressed in the mammalian CNS (Cx36 and Cx43). We anticipated that these experiments would further clarify whether the inconsistent homotypic interaction observed for Cx34.7(M1) hemichannels between our *Xenopus* oocyte and HEK293FT FETCH experiments could potentially limit their application for precision circuit editing in mammals. We also tested our Cx34.7(M1) and Cx35(M1) channels under heterotypic conditions to confirm their in vivo functionality.

Here we capitalized on *C. elegans* as a model for testing, as multiple groups have established that selectively expressing Cx36 is sufficient to reconstitute a functional electrical synapse between two connected neurons. The presence and function of this Cx36–Cx36 synapse has been confirmed through microscopy^8^, measurements of synaptic physiology^8^, calcium imaging^10^ and behaviour^7–12^. Thus, we assessed whether we could induce changes in calcium imaging and behaviour with Cx34.7(M1)–Cx35(M1) in a manner that mirrored Cx36–Cx36.

*C. elegans* do not have an innate temperature preference and can thrive in a broad range of temperatures^38^. However, *C. elegans* trained at a particular temperature in the presence of food will migrate towards that temperature when they are subsequently placed on a temperature gradient^38^. This learned preference is in part mediated by plasticity of the synapse between a thermosensory neuron (AFD, presynaptic) and an interneuron (AIY, postsynaptic)^39^. Notably, plasticity in AFD can be genetically manipulated to affect transmission to AIY and to predictably encode the behavioural preference that must otherwise be learned^10^.

**Fig. 3:**
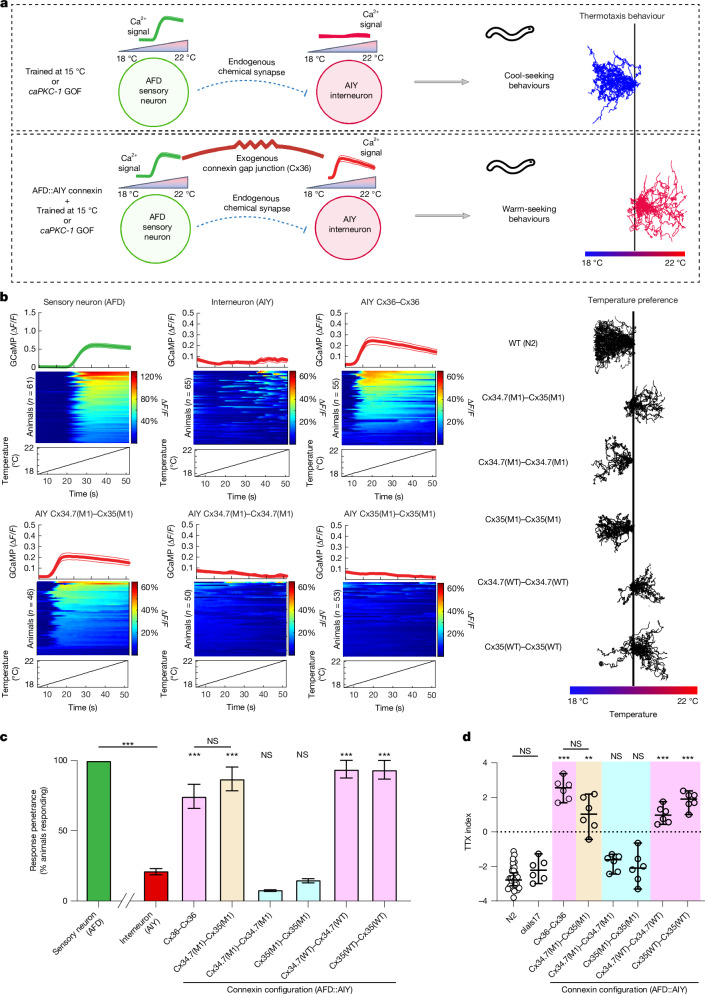
Heterologous connexin hemichannels couple *C. elegans* neurons and recode thermal preference. **a**, Schematic of AFD→AIY synaptic communication and expressed temperature preference. The AFD thermosensory neuron has a robust calcium response to warming stimuli. *C. elegans* raised in the presence of food at 15 °C, or animals with a gain-of-function mutation in the gene encoding protein kinase-C1 (*caPKC-1* GOF), move towards cooler temperatures when placed on a thermal gradient from 18 to 22 °C (top). Heterologous expression of Cx36 hemichannels between AFD and AIY synchronizes AIY and promotes warm-seeking behaviour (bottom). **b**, Left, calcium traces of neurons expressing heterologous connexin hemichannel pairs, along with baseline AFD and AIY responses. Each panel depicts the average group trace (top, mean ± s.e.m.), heatmaps of individual animals (middle) and the temperature stimulus (bottom). Δ*F*/*F* values in heatmaps are presented as percentages (for example, a value of 0.2 for Δ*F*/*F* is 20%). Right, behavioural traces for WT *C. elegans* (N2) and *C. elegans* homotypically expressing WT connexin hemichannels, homotypically expressing mutant connexin hemichannels or heterotypically expressing the mutant pair. **c**, Proportion of animals showing neuronal calcium responses based on the traces in **b**. ***P < 0.0005, two-tailed Fisher’s exact test for penetrance; error bars denote 95% confidence interval (CI). *n* = 61, 65, 55, 46, 50, 53, 49 and 46 animals (left to right). **d**, Thermotaxis preference (TTX) indices for experimental groups. olaIs17, control (expresses GCaMP and fluorophore without connexin hemichannel expression). Each individual point represents the TTX index of a separate assay. *n* = 30, 6, 6, 6, 6, 6 and 6 assays for the groups shown (left to right), *N* = 12–15 animals per assay. The black horizontal line depicts the median for each group. **P < 0.005, ***P < 0.0005 versus N2, two-tailed Dunnett’s T3 multiple comparisons test; error bars denote 95% CI. NS, not significant.

We have previously shown that heterologous expression of Cx36 can be used to edit this circuit by bypassing the presynaptic plasticity mechanisms between AFD and AIY that contribute to the learned temperature preference^10^. Thus, circuit-edited worms show a persistent preference for warmer temperatures (Fig. 3a). We therefore used this circuit to validate the functionality of our engineered gap junction proteins (as assessed by calcium imaging and quantitative behaviour testing).

**Extended Data Fig. 7:**
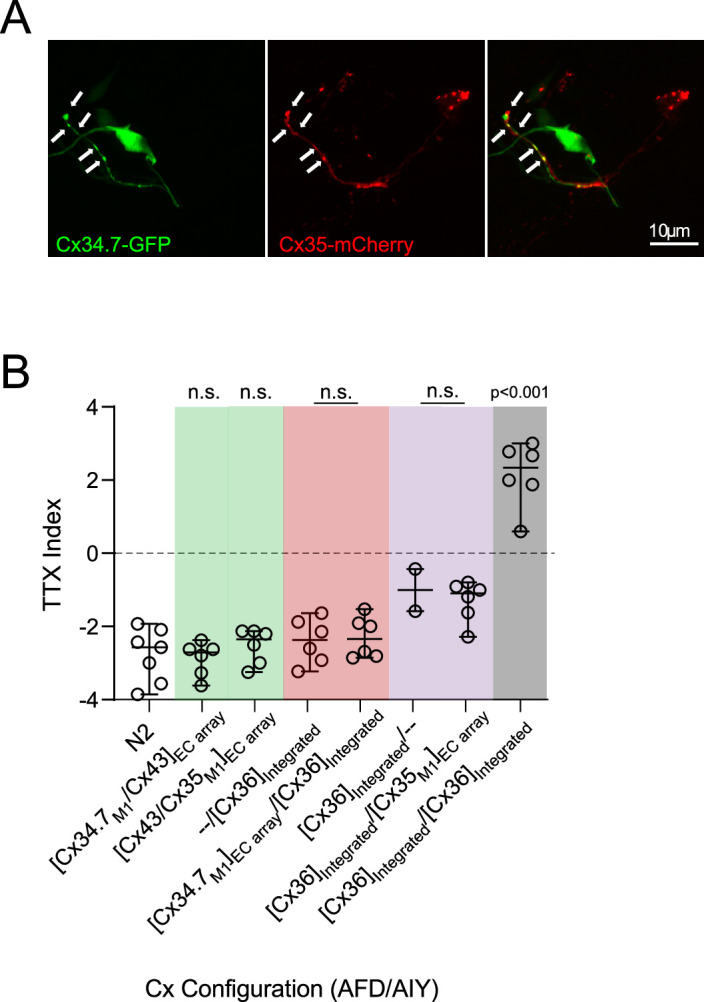
Connexin Cx34.7 and Cx35 do not alter *C. elegans* behavior when expressed as counterparts to connexins endogenous to the human brain. **A)** Confocal maximum intensity projections of *C. elegans* expressing the Cx34.7_M1_/Cx35_M1_ pair. GFP-tagged Cx34.7_M1_ is expressed in the AFD neuron, with puncta along its axon (left). mCherry-tagged Cx35_M1_ is expressed in the AIY neuron, with puncta along its neurite (middle). Composite image showing the colocalizing GFP/mCherry puncta (right). White arrows highlight puncta. The representative image was selected from 30 acquired images. **B)** We tested Cx34.7_M1_ and Cx35_M1_ hemichannels against Cx36 and Cx43. Worms with integrated arrays were used to test the mutant channels against Cx36. TTX Index (Y-axis) refers to the thermotaxis preference index, calculated as previously described^10^. Negative values represent migration towards cold temperatures while positive values indicate migration towards warm temperatures. In the X-axis, N2 refers to the wild type *C. elegans* animals expressing no arrays. The other groups describe the expression of specific arrays in an AFD (presynaptic)/AIY (postsynaptic) configuration. “EC array” = extrachromosomal arrays, “Integrated” = integrated arrays. Experimental worms were directly compared against integrated Cx36 controls, where Cx34.7_M1_ or Cx35 _M1_ expression patterns mirrored our original experimental configuration. Worms expressing extrachromosomal arrays in AFD or AIY were used to test Cx43. *C. elegans* expressing Cx34.7_M1_/Cx36, Cx34.7_M1_/Cx43, Cx36/Cx35_M1_ or Cx43/Cx35_M1_ in AFD/AIY all continued to migrate towards cold temperatures (F_7,10.67_ = 19.29; P < 0.0001, Welch one-way ANOVA followed by a two-tailed Dunnett’s T3 multiple comparisons; P < 0.001 between N2 and Cx36/Cx36), and no differences were observed across experimental pairs (P > 0.05; highlighted by red or purple), indicating they were not forming functional electrical synapses.

We first expressed Cx34.7(M1) in AFD cells and expressed Cx35(M1) in AIY cells (Extended Data Fig. 7a and Supplementary Table 2). Similar to Cx36–Cx36, expression of Cx34.7(M1)–Cx35(M1) in the AFD–AIY pair resulted in functional coupling between AFD and AIY, as assessed by calcium imaging (Fig. 3b, left, and 3c; *P* < 0.0005, two-tailed Fisher’s exact test with FDR correction). The *C. elegans* constitutively migrated towards warmer temperatures when placed on a thermal gradient, which mirrored the behaviour of animals expressing heterologous Cx36–Cx36 (*F*_7,17.91_ = 84.99, *P* < 0.0001, Welch one-way analysis of variance (ANOVA) followed by Dunnett’s T3 multiple comparisons; *P* < 0.005 versus WT N2 animals; Fig. 3b, right, and 3d). Homotypic expression of Cx34.7(WT) or Cx35(WT), but not Cx34.7(M1) or Cx35(M1), in both AFD and AIY neurons synchronized the two cells and modulated behaviour (Fig. 3b–d; *P* < 0.0005 versus WT N2 animals). We also evaluated Cx34.7(M1) and Cx35(M1) hemichannels against Cx36 and CX43. *C. elegans* expressing Cx34.7(M1)–Cx36, Cx34.7(M1)–Cx43, Cx36–Cx35(M1) or Cx43–Cx35(M1) in AFD–AIY pairs all continued to migrate towards cold temperatures (*F*_7,10.67_ = 19.29, *P* < 0.0001, Welch one-way ANOVA followed by Dunnett’s T3 multiple comparisons; *P* > 0.05 for all comparisons against N2 animals; Extended Data Fig. 7b).

Taken together, these findings confirmed that our Cx34.7(M1)–Cx35(M1) electrical synapse modified *C. elegans* behaviour and physiology in a manner that was statistically indistinguishable from the Cx36–Cx36 electrical synapse. Our findings also supported the docking properties we predicted for the mutants using our in vitro screen and in silico studies. That is, both Cx34.7(M1) and Cx35(M1) did not alter behaviour and physiology when expressed in homotypic configurations or in heterotypic configurations against Cx36 and Cx43.

## Cx34.7(M1)–Cx35(M1) enhances circuit synchrony in mice

**Fig. 4:**
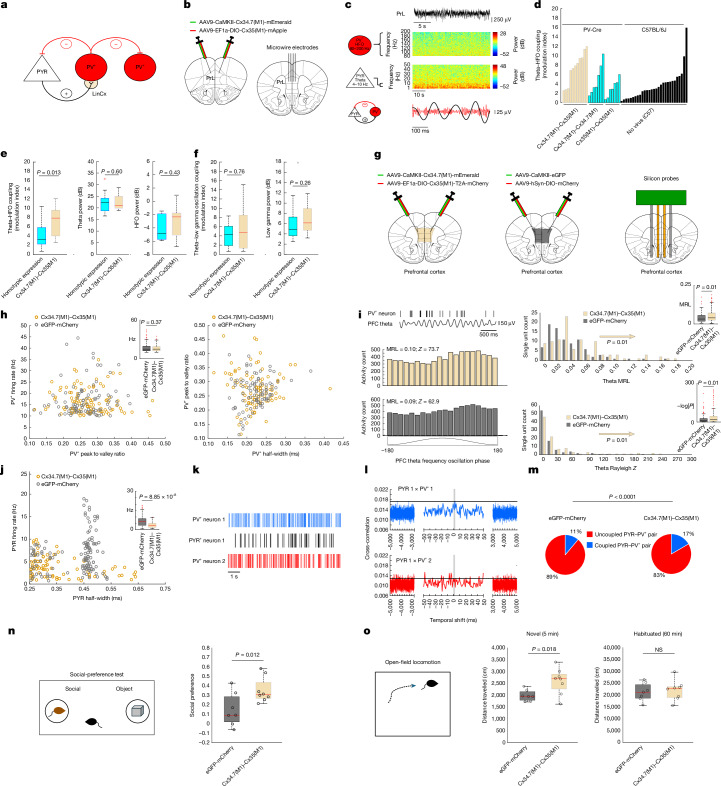
LinCx edits microcircuit dynamics at the millisecond timescale in mice. **a**, Schematic of a prefrontal cortex microcircuit comprising a PYR neuron and PV^+^ interneurons. The tan circle highlights the target for LinCx editing. **b**, PV-Cre mice were injected with AAV-CaMKII-Cx34.7(M1) (green) and AAV-DIO-Cx35(M1) (red) into the prelimbic cortex (PrL) and subsequently implanted with microwires. Control mice were injected with a Cx34.7(M1) or Cx35(M1) pair of viruses to express the same hemichannel in both cell types. **c**, Representative LFPs recorded from the PrL (top row). Power spectrograms show theta and HFO activity, corresponding to PYR neuron and PV^+^ interneuron firing, respectively (middle row). Microcircuit function is represented by the coupling between the phase of theta oscillations (black) and the amplitude of HFOs (red) (bottom row). **d**, Distribution of theta–HFO coupling scores (modulation index) observed across non-injected C57BL/6J mice, PV-Cre mice injected with a Cx34.7(M1) or Cx35(M1) pair of viruses or mice injected with a docking Cx34.7(M1)–Cx35(M1) pair. **e**, Mice with the Cx34.7(M1)–Cx35(M1) pair showed higher theta–HFO coupling (left), but not theta or HFO power (middle, right), than mice expressing Cx34.7(M1) and Cx35(M1) under homotypic conditions (same mice as shown in Fig. 5d). **f**, Circuit editing had no significant impact on theta–low gamma oscillatory coupling (left) or low gamma power (right) for these mice (same mice as shown in Fig. 5d). **g**, PV-Cre mice were bilaterally injected with AAV-CaMKII-Cx34.7(M1) and AAV-DIO-Cx35(M1) (left). Control mice were injected with AAV-CaMKII-eGFP and AAV-DIO-mCherry (middle). Mice were subsequently implanted with silicon probes (right). **h**, Waveform properties of PV^+^ interneurons recorded from Cx34.7(M1)–Cx35(M1) and eGFP-mCherry (control) mice (*P* = 0.29 for peak valley ratio; *P* = 0.88 for half-width). Inset in left-hand graph shows the mean firing rate of PV^+^ interneurons. **i**, Left, activity of PV^+^ interneuron and theta oscillations recorded concurrently from the same channel (top). Theta oscillation phase firing distributions of a PV^+^ interneuron recorded from an experimental mouse (middle) and a control mouse (bottom). Right, distribution of MRLs across the population of PV^+^ interneurons recorded from experimental and control mice (top). Distribution of Rayleigh test statistics across the same populations of PV^+^ interneurons (bottom), in which *Z* = –log [*P*]. Insets show the same data as a box and whisker plot. Same neurons as shown in **h**. **j**, Waveform properties of PYR excitatory neurons recorded from Cx34.7(M1)–Cx35(M1) and control mice. Inset shows the mean firing rate of PYR neurons, which was lower in LinCx-edited mice. **k**, Raster plot showing a PYR neuron and two PV^+^ interneurons that were recorded concurrently. **l**, Cross-correlation between the PYR neuron and two PV^+^ interneurons shown in **k**, determined at temporal shifts up to ±5 s between the spike trains. The horizontal line in each plot is the maximum cross-correlation in the 1–4-ms window. For the PV^+^ interneuron in blue, the maximum cross-correlation in 1–4 ms exceeds more than 98.75% of the values determined at temporal shifts ranging from ±3–5 s (corresponding to *α* = 0.05/4 for the 1–4-ms window), whereas the maximum cross-correlation did not exceed this threshold for the red PV^+^ interneuron. Thus, only the blue PV^+^ interneuron neuron shows coupling to the PYR. **m**, A higher proportion of PYR–PV^+^ pairs with significant short-latency coupling was found in the LinCx-edited mice (right) relative to the control mice (left). **n**, Left, schematic of the social-preference test to assess the impact of PYR–PV^+^ interneuron microcircuit editing on social behaviour. Right, LinCx-edited mice exhibited an increase in social preference relative to control mice. **o**, LinCx-edited mice exhibited higher exploratory drive in a novel environment compared with controls (left, middle). No group difference in gross locomotor behaviour was observed following habituation. Same mice as shown in **n**. For *n* values and statistical tests, see main text. For definitions of box plots, see Methods. Brain slice images in **b** and **g** were adapted from ref. ^55^.

**Extended Data Fig. 8:**
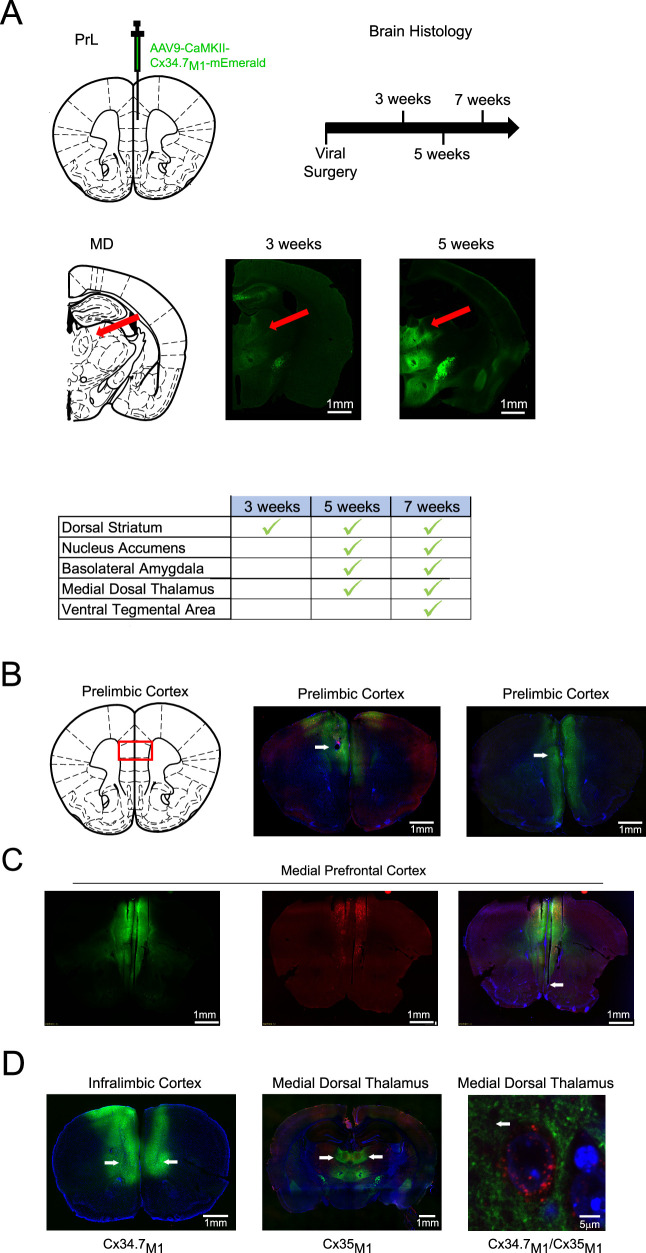
Representative histological images for mouse circuit-editing experiments. **A)** Time course of pronounced Cx34.7 expression and trafficking in prelimbic cortex soma and terminals. Five C57BL/6J mice were infected with AAV9-CaMKII-Cx34.7_M1_-mEmerald in prelimbic cortex (PrL; top left). Histology was performed 3 weeks (N = 1 mouse), 5 weeks (N = 2 mice), or 7 weeks (N = 2 mice) after viral surgeries (top right). Red arrows highlight the medial dorsal thalamus (MD) target for the tail suspension test experiments (middle). Note the prominent trafficking of CaMKII-Cx34.7_M1_-mEmerald apparent at 5 weeks (the time course utilized for our behavioral experiments; middle right). The table on the bottom indicates the post-surgical timeline at which prominent CaMKII-Cx34.7_M1_-mEmerald expression was observed in each region. **B-D)** Representative histological images for mouse circuit-editing experiments. **B)** Representative histological image of prelimbic cortex in a Cx34.7_M1_/ Cx35_M1_ infected mouse (left) and a Cx34.7_M1_/ Cx34.7_M1_ control (middle). White arrows highlight electrode tracks. For mice included in the prelimbic cortex editing experiment, individual electrode wires were distributed within the area highlighted by the red rectangle (right). Representative of N = 26 mice. **C)** Histological images of a PV-Cre/Vglut2-flp mouse injected in medial prefrontal cortex with AAV9-CaMKII-Cx34.7_M1_-mEmerald (left) and AAV9-DIO-Cx35_M1_-T2A-mCherry (middle). Overlay is shown to the right. White arrow highlights the tip of one of the two shanks traversing the medial prefrontal cortex. Representative of N = 6 mice. **D)** Histological image of mouse injected with AAV9-CaMKII-Cx34.7_M1_-mEmerald in infralimbic cortex (left). Image showing medial dorsal thalamus in mouse injected with AAV9-CaMKII-Cx34.7_M1_-mEmerald in infralimbic cortex and AAV9-CaMKII-Cx35_M1_-mApple in medial dorsal thalamus (middle). White arrows highlight viral injection tracks. Composite confocal image of medial dorsal thalamus showing expression of Cx35_M1_-mApple (red) at the soma of a cell stained with DAPI (blue) and Cx34.7_M1_-mEmerald (green) at IL nerve terminals (right). Representative of N = 26 mice. Brain slice images in **a** and **b** were adapted from ref. ^55^.

Having established the in vivo docking selectivity and functionality of our Cx34.7(M1)–Cx35(M1) pair, we set out to determine whether these proteins can modulate mesoscale neural circuitry in mammals. After verifying their expression and trafficking (Extended Data Fig. 8a), we chose to edit a circuit composed of two distinct cell types. Mice are an ideal species in which to test cell-type specificity because they are highly amenable to cell-type-specific access via selective promoters and Cre-recombinase targeting. Excitatory pyramidal neurons (PYR) and parvalbumin-expressing fast-spiking (PV^+^) interneurons can form microcircuits whereby PYR neurons excite PV^+^ interneurons, which in turn inhibit PYR neurons (Fig. 4a). This PYR–PV^+^ interneuron microcircuit has been well characterized in the hippocampus. In detail, medial prefrontal PYR neurons show activity coupled to the phase of hippocampal theta frequency oscillations (4–10 Hz) during spatial exploration^40^, whereas PV^+^ interneuron activity is coupled to hippocampal gamma frequency oscillations (30–80 Hz)^41^. Notably, the activity of this PYR–PV^+^ interneuron microcircuit is reflected in the synchrony between the phase of theta oscillations and the amplitude of gamma oscillations in rodents^42^.

PYR–PV^+^ interneuron microcircuits are also observed in the prefrontal cortex, but with slightly different neurophysiological properties^43^. As observed in the hippocampus, prefrontal cortex PYR neurons phase-couple to locally recorded theta oscillations^44^. Conversely, prefrontal PV^+^ interneurons best couple to the phase and amplitude of local high-frequency oscillations (HFOs, 80 ≠ 200 Hz)^45^. Thus, to determine the effect of our electrical synapse, we quantified the coupling between the phase of prefrontal cortex theta oscillations and the amplitude of prefrontal cortex HFOs as a proxy for prefrontal PYR–PV^+^ interneuron microcircuit activity. Specifically, we expressed our Cx34.7(M1)–Cx35(M1) synapse at the PYR–PV^+^ interneuron interface. We proposed that this manipulation would enhance the coupling between theta oscillations and HFOs in the prefrontal cortex.

We developed an AAV virus (AAV9-Ef1α-DIO-Cx35(M1)) to selectively target Cx35(M1) to cells expressing Cre-recombinase and another virus (AAV9-CaMKII-Cx34.7(M1)) to express Cx34.7(M1) nonselectively across all neurons. We then injected PV-Cre mice with both viruses bilaterally in the prelimbic cortex (*n* = 11; Fig. 4b). A group of PV-Cre control mice was injected with viruses to only express Cx34.7(M1) or Cx35(M1) nonselectively across all neurons (*n* = 7 per connexin; Methods and Extended Data Fig. 8b). Finally, we also tested a third group of control C57BL/6J mice that had not been injected with virus (*n* = 29). Neural oscillatory activity was recorded from the prelimbic cortex while mice explored an open field.

To determine the coupling between theta oscillations (4–10 Hz) and HFOs (80–200 Hz), we isolated local field potential (LFP) activity in these two frequency bands (Fig. 4c). We then determined their phase–amplitude coupling relationships using the established modulation index (*z* score), which quantifies the statistical likelihood that measured relationships between two oscillations would be observed by chance^46^ (Methods). We found significant theta–HFO coupling from the majority of implanted mice (80%, 43 out of 54; Fig. 4d). We then tested whether coupling was higher in mice expressing our electrical synapse than in controls, a result that signified an increase in electrical coupling. Indeed, theta–HFO coupling was significantly higher in the LinCx-expressing mice than in the pooled group of control mice expressing the connexins under homotypic configurations (*U* = 141, *P* < 0.013, one-tailed rank-sum test, Cohen’s *D* = 0.99; Fig. 4e). Thus, expression of the synapse was sufficient to enhance coupling in a microcircuit defined by two precise cell types in mammals. Notably, our post hoc analysis did not show differences in theta–HFO coupling between mice expressing homotypic Cx34.7(M1)–Cx34.7(M1) or Cx35(M1)–Cx35(M1) synapses compared with uninfected C57BL/6J control mice (*U* = 509 and *P* = 0.28 for Cx34.7(M1)–Cx34.7(M1), *U* = 542 and *P* = 0.84 for Cx35(M1)–Cx35(M1), two-tailed rank-sum test). These findings support the heterotypic selectivity of the two mutant proteins in vivo.

In our secondary analyses, there were no differences in theta or HFO power between mice expressing the synapse and the control mice expressing mutant connexins in homotypic configurations across the PYR–PV^+^ interneuron microcircuit (*U* = 172 and *P* = 0.60 for theta power, *U* = 167 and* P* = 0.43 for HFO power, two-tailed rank-sum test; Fig. 4e). Similarly, no group differences in theta–low gamma oscillation cross-frequency phase coupling (*U* = 176 and *P* = 0.76, two-tailed rank-sum test; Fig. 4f) or low gamma oscillation power (*U* = 161 and *P* = 0.26, two-tailed rank-sum test) were observed (Fig. 4f). Thus, our electrical synapse selectively increased the synchrony between theta and HFO activity in the medial prefrontal cortex.

Next, we tested whether this increased synchrony could be observed at the level of single neurons. Three new experimental PV-Cre mice were infected with AAV9-hsyn-DIO-Cx35(M1)-T2A-mCherry to selectively target Cx35(M1) to cells expressing Cre-recombinase and with the nonselective AAV9-CaMKII-Cx34.7(M1)-mEmerald virus. Three control mice were infected with AAV9-hsyn-DIO-mCherry and AAV9-CaMKII-eGFP. Two weeks later, these mice were implanted with high-density silicon recording probes (Fig. 4g and Extended Data Fig. 8c). Following recovery, neural activity was recorded for 10 min while mice were in their home cage. PV^+^ interneurons were identified on the basis of previously validated waveform criteria (for example, a peak to valley ratio of <1.1 and a mean firing rate of >10 Hz)^47^. No group differences in waveform properties were observed (*t*_190_ = 1.07 and *P* = 0.29 for peak valley ratio, *t*_190_ = 0.15 and *P* = 0.88 for half-width, unpaired two-tailed *t*-tests; *n* = 91 and 101 total medial prefrontal cortex PV^+^ interneurons for the experimental and control groups, respectively; Fig. 4h).

**Extended Data Fig. 9:**
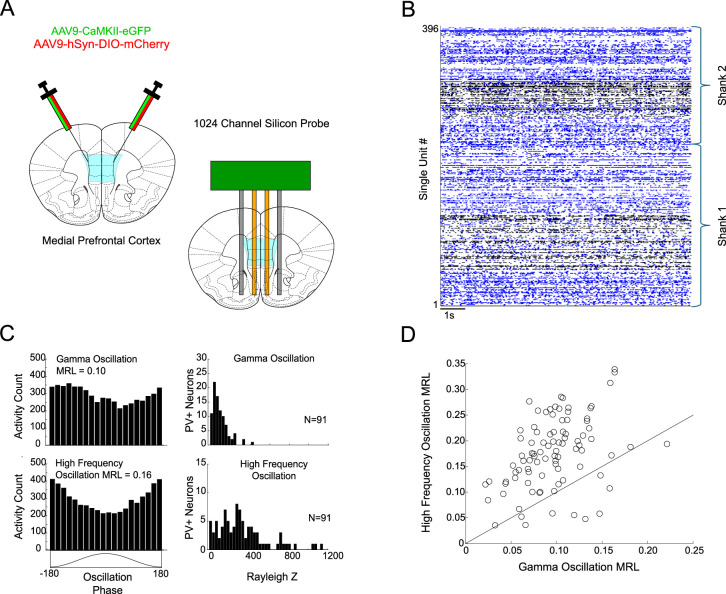
Medial Prefrontal Cortex (mPFC) PV+ interneurons optimally phase lock to high frequency oscillations. **A)** Viral infection and silicon probe implantation targets in control mice used for analysis. **B)** Raster plot showing 396 single units recorded concurrently from two implanted shanks that traversed the mPFC in a female mouse. PV+ interneurons from mPFC are depicted in black. **C)** Histograms depicting a mPFC PV+ interneuron firing relative to the phase of gamma oscillations (30-80 Hz; top left) and high frequency oscillations (80-200 Hz; bottom left) recorded from the same channel as the neuron. Histogram showing phase locking of 91 PV+ interneurons to gamma oscillations (top right) and high frequency oscillations (bottom right) are shown to the right. Data was acquired from two female mice and one male mouse. All the PV+ interneurons showed phase locking to both oscillations. **D)** Mean resultant length (MRL) for all recorded PV+ interneurons showing coupling to gamma and high frequency oscillations. The diagonal line corresponds to equivalent coupling to both oscillations. We observed higher phase coupling to high frequency oscillations than to gamma oscillations (t_90_ = 12.2, P = 6 × 10^−21^, two-tailed paired t-test, N = 91 neurons). Brain slice images in **a** were adapted from ref. ^55^.

After establishing that the activity of prefrontal cortex PV^+^ interneurons was better coupled to the phase of HFOs than gamma oscillations (Extended Data Fig. 9), we compared the activity profiles of PV^+^ interneurons across the two groups. We did not observe group differences in the mean firing rate of PV^+^ single units (*t*_190_ = 0.90 and *P* = 0.37 for comparisons of firing rate, unpaired two-tailed *t*-test; Fig. 4h). This result was consistent with our finding that the electrical synapse did not affect the amplitude of HFO activity in the prefrontal cortex (Fig. 4e, right). Next, we quantified the coupling of activity for each PV^+^ interneuron to local theta oscillations by determining its mean resultant length (MRL) and with the Rayleigh test of circular uniformity (Fig. 4i). In contrast to a previous study^45^, most PV^+^ interneurons showed phase coupling to theta oscillations in both groups (69 out of 91 and 83 out 101 neurons for experimental and control mice, respectively). As proposed, the experimental mice showed stronger PV^+^ phase coupling to theta oscillations than the control group (*t*_190_ = 2.34 and *P* = 0.01 for MRL, *t*_190_ = 2.31 and *P* = 0.01 for Rayleigh *Z*, unpaired one-tailed *t*-tests, Cohen’s *D* = 0.33; Fig. 4i). Thus, the engineered electrical synapse increased the coupling of PV^+^ interneurons to theta oscillations, again consistent with our observations for the coupling of HFO activity.

Next, we directly quantified coupling between cells in the PYR–PV^+^ interneuron microcircuit. We isolated 128 and 79 putative PYR neurons from experimental mice and control mice, respectively (Fig. 4j), on the basis of previous criteria (for example, a spike half-width of >250 µs and a mean firing rate of <20 Hz)^48^. Incidentally, we observed a reduced PYR neuron firing rate in the LinCx-edited mice (*t*_205_ = 5.56 and *P* = 8.58 × 10^−8^, two-tailed unpaired *t*-test; Fig. 4j, inset). We then performed cross-correlation analysis between 5,616 pairs of concurrently recorded PYR neurons and PV^+^ interneurons (Fig. 4k) and used permutation testing to determine whether each pair was significantly coupled with short latency (1–4 ms; Fig. 4k,l, Methods and Supplementary Fig. 2). We found that 312 out of 2,801 PYR–PV^+^ pairs (11%) were significantly coupled in the control mice. A higher proportion of PYR–PV^+^ pairs (17%) were significantly coupled in the LinCx-edited mice (468 out of 2,815 pairs; *χ*^2^ = 35, *P* < 0.0001, chi-square test, *Φ* = 0.08; Fig. 4m). Together, these data demonstrate that our electrical synapse selectively increases millisecond timescale coupling in the PYR–PV^+^ interneuron circuit in the medial prefrontal cortex.

Finally, increased prefrontal cortical excitability and microcircuit dysfunction have been implicated in mediating social deficits in autism. As LinCx editing decreased prefrontal cortex excitability and increased microcircuit coupling, we tested whether this manipulation would enhance social behaviour. LinCx-edited mice exhibited a higher preference for the social stimulus in a social preference task (*t*_13_ = 2.55 and *P* = 0.012 for LinCx-edited versus control mice, unpaired one-tailed *t*-test, *n* = 7–8 per group, Cohen’s *D* = 1.1; Fig. 4n). Notably, LinCx-edited mice also exhibited increased exploratory drive when placed in a novel open-field (*t*_13_ = 2.69 and *P* = 0.018 for LinCx-edited versus control mice, two-tailed unpaired *t*-test; Fig. 4o, left and middle). No differences in gross locomotor behaviour were observed when animals were previously habituated to the testing arena (*t*_13_ = 0.37 and *P* = 0.72 for LinCx-edited versus control mice, two-tailed unpaired *t*-test; Fig. 4o, right). Overall, these findings show that expression of the LinCx electrical synapse causally enhances coupling of single-unit activity in a local mammalian cortical microcircuit and modifies behaviour accordingly.

## Cx34.7(M1)–Cx35(M1) potentiates a long-range circuit

**Fig. 5:**
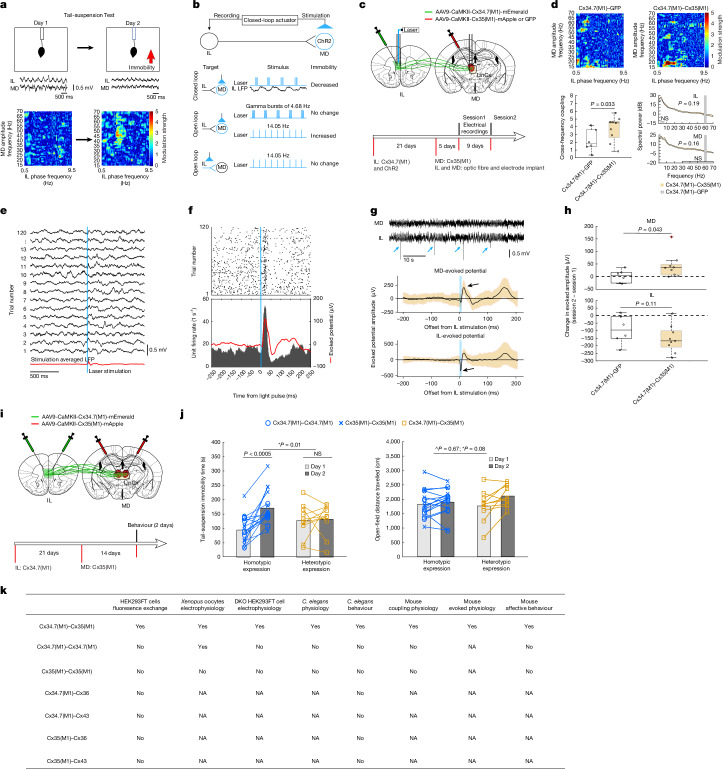
LinCx edits a long-range circuit in mice. **a**, Behavioural and neurophysiological impacts of repeated tail-suspension stress (data from ref. ^50^). Bottom, increased coupling between IL 2–7 Hz oscillations and MD 30–70 Hz oscillations due to repeat tail-suspension test exposure. **b**, Top row, schematic of the closed-loop optogenetic approach to synchronizing MD firing to IL oscillations as performed in our previous work^50^. Second row, behavioural impact of causally coupling MD activity to IL 2–7 Hz oscillations using closed-loop optogenetic stimulation. Middle and bottom rows, behavioural outcomes of stimulating MD (middle) or IL terminals (bottom) in the MD with a pattern uncoupled to ongoing IL activity (open-loop optogenetic stimulation). **c**, Viral and optical fibre or electrode targeting approach (top), and experimental timeline for optogenetic interrogation of the IL→MD circuit (bottom). **d**, Representative plots showing coupling between IL and MD oscillations for Cx34.7(M1)–GFP control mice (top left) and Cx34.7(M1)–Cx35(M1) mice (top right). Mice injected with the Cx34.7(M1)–Cx35(M1) pair showed higher coupling between IL 2–7 Hz and MD 30–70 Hz oscillations (bottom left), but there were no group differences in IL 2–7 Hz or MD 30–70 Hz oscillatory power (bottom right; data shown as the mean ± s.e.m.; note that the distributions overlap). **e**, Representative LFP oscillations from the MD in response to an optogenetic light pulse in the IL (light blue vertical line). LFP activity was averaged across 120 light pulses (1 mW, 10 ms pulse width) to produce the MD-evoked potential in red. **f**, MD neuronal response to an optogenetic light pulse in the IL (light blue vertical line). Data shown for MD unit M1P1-135. Cellular activity across 120 light pulses is shown below, with the evoked potential recorded from the same channel overlaid in red. The positive deflection in the evoked potential reflects firing of the neuron. **g**, Top, representative LFP oscillations recorded from the IL and MD during light stimulation. Note the four highlighted supraphysiological IL responses induced by light stimulation (light blue arrows). Representative mean light-evoked potential recorded concurrently from an MD (middle) and IL microwire (bottom). Note the large negative instantaneous deflection in the IL channel and the positive deflection the MD channel (black arrows). Data shown as the mean ± s.d. across about 30 light pulses. **h**, Changes in amplitude of evoked potential across sessions in the MD (top) and IL (bottom) in mice expressing Cx34.7(M1)–Cx35(M1) versus control mice. Same mice as shown in **d**. **i**, Viral injection strategy and experimental timeline for quantifying the impact of IL→MD LinCx editing on behaviour. **j**, Immobility time during repeat tail suspension (left) and distance travelled during repeat open-field testing (right). A pooled group of mice injected with Cx34.7(M1) or Cx35(M1) in homotypic non-docking configurations showed stress-induced behavioural adaptation during repeat tail-suspension testing, whereas mice injected with the functional Cx34.7(M1)–Cx35(M1) pair did not. No behavioural differences were observed between viral groups in the open-field test (^group effect; *group × day interaction effect, mixed-effects model ANOVA). Bars reflect group means. **k**, Summary of biophysical and electrophysiological properties of Cx34.7(M1) and Cx35(M1). DKO, double knockout; NA, not applicable. For *n* values and statistical tests, see the main text. For definitions of box plots, see Methods. Brain slice images in **c** and **i** were adapted from ref. ^55^.

We next tested whether our electrical synapse could potentiate a long-range circuit consisting of cells in two different brain regions. The infralimbic cortex (IL, an anatomical subdivision of the mouse medial prefrontal cortex) sends a monosynaptic projection to medial dorsal thalamus (MD) in mice. We selected this circuit to test the functionality of Cx34.7(M1)–Cx35(M1) given our previous experience in quantifying its physiological properties and role in stress behaviour^49,50^. Specifically, the tail-suspension test is a classical assay that measures the behavioural response of mice to an inescapable negative experience in which they are suspended upside down by their tail^51^. Exposure to stress reduces behavioural responses during the assay^52^, and the assay induces a robust stress response^53^. Thus, repeated exposure to the tail-suspension test increases immobility during subsequent testing^50^ (Fig. 5a, top). This behavioural adaptation is specific to the stress context, as decreased mobility is not observed when locomotor behaviour is quantified in an open field immediately before each tail-suspension stress session^50^.

In our previous work, we found that exposure to the tail-suspension test induced coupling between low-frequency oscillations in the IL and low gamma oscillations in the MD^50^ (Fig. 5a). Furthermore, when we exogenously recapitulated coupling between the IL and MD using a brain–machine interface, mice showed reduced behavioural adaptation in the assay^50^. Stimulation of the MD in a manner that was uncoupled to IL activity did not produce this outcome^50^ (see Fig. 5b for a summary). Together, these findings established the role of the IL→MD circuit in stress compensatory behaviour^50^.

In the current study, we injected BALB/cJ mice with AAV9-CaMKII-Cx34.7(M1) and AAV9-CaMKII-ChR2 in the left IL. Three weeks later, we injected these mice with AAV9-CaMKII-Cx35(M1) in the left MD and implanted microwire recording electrodes in the IL and MD (Fig. 5c). After another 5 days of surgical recovery, we recorded baseline LFP activity and activity in response to 10-ms pulses of optogenetic stimulation to the IL. This timeline ensured expression of ChR2 and Cx34.7(M1) in the IL, but minimal trafficking of Cx34.7(M1) to the IL axonal terminals in the MD (Extended Data Fig. 8a,d) and minimal local expression of Cx35(M1) in the MD. We acquired additional recording and stimulation data 9 days later (session 2: 5 weeks after the initial IL injection and 2 weeks after the MD injection), which enabled strong trafficking of Cx34.7(M1) to the IL and substantial local Cx35(M1) expression. A control group was infected with AAV9-CaMKII-GFP in the MD instead of Cx35(M1).

We first compared coupling across the IL→MD circuit between the two groups 2 weeks after the MD injection (that is, a second baseline recording session). As proposed, mice injected with Cx35(M1) showed stronger coupling between IL 2–7 Hz oscillations and MD 30–70 Hz oscillations than mice injected with GFP (*U* = 88 and *P* = 0.033, one-tailed rank-sum test, Cohen’s *D* = 0.92; Fig. 5d, top and bottom left). No group differences were observed in MD 30–70 Hz power (*U* = 80 and *P* = 0.19, two-tailed rank-sum test) or in IL 2–7 Hz power (*U* = 81 and *P* = 0.16, two-tailed rank-sum test; Fig. 5d, bottom right). Thus, expression of Cx34.7(M1)–Cx35(M1) enhanced oscillatory coupling across the IL→MD circuit as it had for the prelimbic cortex PYR–PV^+^ interneuron microcircuit (Fig. 4).

Next, we directly interrogated the IL→MD circuit in mice expressing Cx34.7(M1)–Cx35(M1). In our previous study, we observed a positive evoked potential in the MD within 25 ms of IL stimulation^49^. We first confirmed that this evoked response directly reflects the activation of single units in the MD (Fig. 5e,f and Supplementary Fig. 3) to establish its local relevance. During our first recording session, we again observed a positive evoked potential in the MD within 25 ms of IL stimulation with 1 mW of blue light (Fig. 5g and Supplementary Fig. 4). We proposed that electrical synapse expression would strengthen this response. Indeed, when we repeated our stimulation experiment 9 days later, mice expressing Cx35(M1) showed an increase in the amplitude of their evoked MD activity (*n* = 9 experimental mice, 41 ± 16 mV). This increase was significantly higher than what we observed from the control group across sessions (*n* = 6, 1 ± 10 mV for control mice, *t*_13_ = 1.9 and *P* = 0.043 for group comparisons, one-tailed *t*-test, Cohen’s *D* = 0.91; Fig. 5h, top). There was no group difference in the change in evoked response amplitude in the IL (–154 ± 30 mV and –93 ± 38 mV for the Cx35(M1) and GFP groups, respectively, *t*_13_ = –1.3, *P* = 0.11, one-tailed *t*-test; Fig. 5h, bottom). Taken together, these findings provided causal evidence that expression of our synapse potentiates the IL→MD circuit.

## Cx34.7(M1)–Cx35(M1) modifies stress behaviour

Finally, we set out to determine whether expressing our engineered electrical synapses across a long-range circuit can modify behaviour. We have previously shown that exogenous stimulation of the IL→MD circuit using closed-loop optogenetic stimulation enhances stress compensation^50^. Therefore, we proposed that expression of the Cx34.7(M1)–Cx35(M1) electrical synapse across the IL→MD circuit would also enhance stress compensation and reduce the stress adaptation observed between the two sessions of the tail-suspension test (that is, increased immobilization). We injected mice with AAV9-CaMKII-Cx34.7(M1) bilaterally into the IL followed by a second injection of AAV9-CaMKII-Cx35(M1) bilaterally in the MD 3 weeks later (*n* = 10 mice; Fig. 5i and Extended Data Fig. 8d). A negative control group of mice was injected with either AAV9-CaMKII-Cx34.7(M1) (*n* = 8) or AAV9-CaMKII-Cx35(M1) (*n* = 8) in both regions. All mice were subjected to 2 days of testing in an open field and with tail suspension after 2 weeks of recovery.

Mice expressing the Cx34.7(M1)–Cx35(M1) hemichannel pair across the IL→MD circuit did not show significant behavioural adaptation in response to repeat tail-suspension testing (*F*_1,24_ = 7.85 and *P* = 0.01 for group × day interaction effect, mixed-effects model ANOVA, *t*_9_ = 0.19 and *P* = 0.85 for post hoc testing, two-tailed paired *t*-test for Cx34.7(M1)–Cx35(M1) mice across days, Cohen’s *D* = 1.0 for change in immobility across groups; Fig. 5j, left). Increases in immobility were observed in the negative control group (*t*_18_ = 4.9 and *P* = 1.7 × 10^−4^ for post hoc testing, two-tailed paired *t*-test for pooled group of Cx34.7(M1)–Cx34.7(M1) and Cx35(M1)–Cx35(M1) mice across days). Moreover, post hoc analysis revealed increases in immobility in the Cx34.7(M1)–Cx34.7(M1) and Cx35(M1)–Cx35(M1) control groups expressing the mutant hemichannels in non-docking configurations independently (*t*_7_ = 3.3 and *P* = 0.01 for Cx34.7(M1)–Cx34.7(M1), *t*_7_ = 5.5 and *P* = 9.5 × 10^−4^ for Cx35(M1)–Cx35(M1), two-tailed paired *t*-test). Moreover, these control mice showed increases in tail-suspension immobility that were statistically indistinguishable from that observed in uninfected BALB/cJ mice (Supplementary Fig. 5). No significant differences in open-field exploration were observed between the mice that expressed Cx34.7(M1)–Cx35(M1) and control mice expressing the hemichannels in non-docking configurations (*F*_1,24_ = 0.19 and *P* = 0.67 for group effect, *F*_1,24_ = 7.69 and *P* = 0.01 for day effect, *F*_1,24_ = 3.4 and *P* = 0.08 for group × day interaction effect, mixed-effects model ANOVA) (Fig. 5j, right). Thus, expression of Cx34.7(M1)–Cx35(M1) in a long-range circuit selectively affects behaviour in mice.

## Discussion

To edit brain circuits in mammals, we created an electrical synapse based on two Cx36 homologues. All our preparations supported the formation of exclusively heterotypic gap junctions between Cx34.7(M1) and Cx35(M1), and the physiological and behavioural outcomes of circuit editing were only observed in mice when we expressed these hemichannels under heterotypic conditions (Fig. 5). We also verified that these hemichannels did not modify behaviour when expressed heterotypically against Cx36 and Cx43 (Extended Data Fig. 7b). Thus, our results support the use of this engineered electrical synapse for precision circuit editing in mammals (Figs. 4 and 5). Notably, we found an increase in the calculated effect sizes of the impacts of LinCx editing in mice as we moved up towards larger levels of analysis (cells to circuits to networks to behaviour; Supplementary Table 3). This finding raises the intriguing idea that small physiological changes induced by LinCx at the level of single-unit coupling may scale their way through neural systems to exert magnified effects on behaviour. Future work will explore this phenomenon in more detail.

Like established protein-based modulation tools, such as optogenetics and DREADDs, LinCx can be targeted to precise cell types in mammals. However, LinCx builds on these technologies by enabling each hemichannel to be expressed in a different cell type. The hemichannels expressed by these two distinct cell types then dock in vivo to form an electrical synapse. Thus, LinCx offers enhanced spatial precision compared to optogenetics and DREADDs in that it enables targeting of one of the specific spatial features that constrain circuits (for example, the structural integration of two distinct cell types). Moreover, LinCx does not require an exogenous actuator, such as light, electricity or a pharmacological compound. Rather, LinCx leverages endogenous brain activity to modulate target neurons to provide a tool for precise circuit editing. Of importance, the impact of LinCx editing on circuit function depends on the physiological properties of the cell types that constitute a circuit, including their resting membrane potentials, input resistance and basal firing rates. Thus, LinCx editing is unlikely to produce identical physiological outcomes across all neural circuits. However, we suggest several approaches to assess how LinCx affects in vivo neural activity. High-density single-unit recordings (as demonstrated here) or wide-field two-photon imaging can be used to assess correlated firing between distinct cell types. Alternatively, when the manner in which cellular activity integrates across levels of analysis is known for a targeted circuit, it may be feasible to assess the impact of LinCx expression on a mouse-by-mouse basis using a population-based measure of neural activity, such as LFPs.

The integrated engineering approach we used to develop LinCx (Extended Data Fig. 1) can be used to develop a toolbox of connexin protein pairs that exhibit selective docking properties. Future work may also reveal new hemichannel pairs with customized conductance properties, mirroring approaches applied to modify the conductance of invertebrate electrical synapses^35^. Thus, it may be possible to use multiple novel LinCx pairs in the same animal to simultaneously edit distinct circuit components and regulate behaviour. LinCx can also be used alongside other well-established preclinical modulation approaches, including DREADDs and optogenetics, to facilitate broad manipulation of brain networks across multiple scales of spatial, temporal and contextual resolution concurrently. Finally, LinCx could potentially be used to edit neural circuits outside the CNS for therapeutic purposes or to enhance emerging cellular-based therapies that use or target specific connexin-expressing cells.

## Limitations

There are several important limitations of our LinCx approach. First, like previous circuit-editing approaches based on inserting gap junctions between specific cell types, our approach is only suitable for editing circuits composed of cells that make physical contact. Second, LinCx has the potential to generate mixed synapses (in neurons) for which chemical and electrical synapses operate in parallel. As our connexin channels are chronically expressed, we anticipate that LinCx also induces activity-dependent changes in local chemical synapses. Indeed, this plasticity at chemical synapses may contribute to the physiological and behavioural changes we observed in our mouse assays.

**Extended Data Fig. 10:**
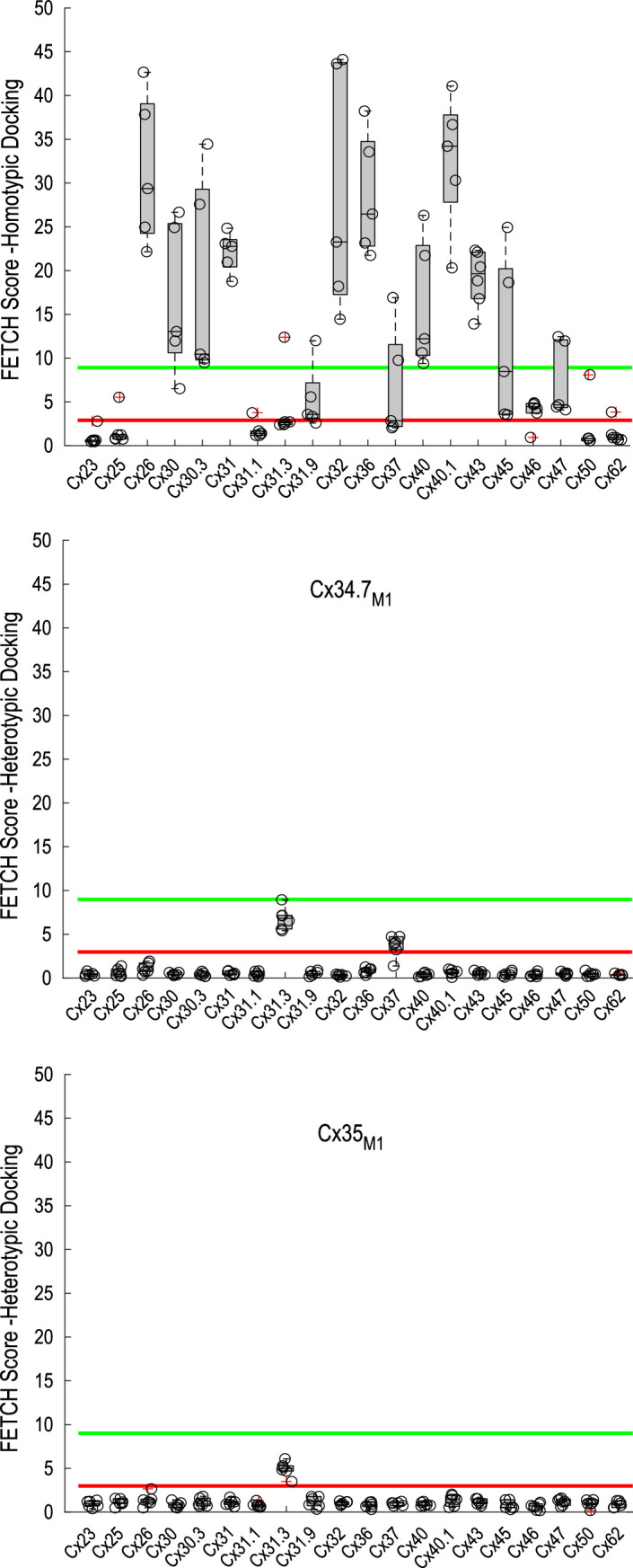
Docking compatibility of Cx34.7_M1_ and Cx35_M1_ with human connexins. We used FETCH to quantify the homotypic docking characteristics of 20 human connexin isoforms (top). We then characterized the heterotypic docking characteristic of our mutant proteins with the 20 endogenous isoforms (middle and bottom). The red and green lines correspond to the upper and lower limits of non-docking and docking population shown in Fig. 1c, respectively. Notably, expression of our Cx59 constructs appeared to promote cell death and was thus excluded from our studies. Additionally, we were unable to establish that Cx23, Cx25, Cx31.1, Cx46, Cx50, and Cx62 were amenable to FETCH analysis given very low homotypic FETCH scores. We did not perform statistical analysis across all the pairs shown here. Rather, we provide this data to highlight potential interactions that our engineered proteins may exhibit with other endogenous connexins, and to guide future experiments. For each Cx box and whisker plot: central mark is the median, box edges are the 25th and 75th percentiles, whiskers extend to extreme data points that are not outliers, and outliers are plotted individually as “+.” (MATLAB, The MathWorks, Inc., Natick, MA).

We engineered Cx34.7 and Cx35 mutant proteins to be docking-incompatible with Cx43 and Cx36. As there are other connexin proteins expressed by mammals, we also used FETCH to screen our connexin mutants for heterotypic docking with other human connexin proteins. We observed FETCH scores for Cx31.3 and Cx37 that were higher than the docking-incompatible pairs we used for our initial analysis (Extended Data Fig. 10) but lower than the docking-compatible pairs. Cx31.3 is expressed in the parenchyma of the mammalian CNS^54^, whereas Cx37 is not. Finally, we were unable to quantify docking for all human isoforms using FETCH analysis (Extended Data Fig. 10). Thus, future work is warranted to assess the docking compatibility of our mutant pairs with Cx31.3 in vivo, and the functional significance of any additional putative docking interactions, for CNS applications of LinCx.

Our engineered hemichannels did not modify circuit function and behaviour when expressed homotypically. For Cx35(M1), these findings were consistent across our FETCH, paired oocyte and mouse experimental findings. However, our paired oocyte electrical recordings indicated that Cx34.7(M1) formed functional homotypic channels. To better understand the potential impact of this discrepancy on the function of our mutated proteins in mammals, we also quantified the function of Cx34.7(M1) using an engineered HEK293FT cell line in which endogenous Cx43 and Cx45 were knocked out to reduce its electrical coupling. In this cell line, homotypic expression of Cx34.7(M1) did not induce functional channels (Fig. 2l), a result that clarifies that the discrepancy was solely observed in the *Xenopus* oocytes system. This discrepancy may be due to protein interactions that restored the docking capacity of Cx34.7(M1) in *Xenopus* oocytes. Future experiments may help to clarify these mechanisms and aid in further optimization of Cx34.7(M1).

Finally, we cannot exclude the possibility that our mutants oligomerize with endogenous Cx36 in mammals to generate heteromeric hemichannels. Indeed, any such heteromeric channels may exhibit docking properties that are distinct from homomeric hemichannels composed exclusively of Cx36, Cx34.7(M1) or Cx35(M1), thereby ultimately limiting the functionality of LinCx across some neural circuits. Future work to assess and optimize the oligomerization specificity for Cx34.7(M1) and Cx35(M1) may further enhance the applicability and utility of LinCx to mammalian neural circuit editing. Future work may also generate new features to regulate the cellular-level directionality of Cx34.7–Cx35 in vivo^15^.

## Methods

### Design of the Cx34.7 and Cx35 mutant library

A semi-rational design approach was used to design the mutant library. Sequence alignments between the *M. americana* connexins and the connexins for which the most structure–function data existed (Cx26, Cx32, Cx36, Cx40 and Cx43) were performed in ClustalW. Sites identified by previous studies as conferring specificity for docking were used, as well as those identified by homology modelling from the structures of Cx26 (ref. ^56^). Specifically, we primarily focused on residues in the extracellular loops: four residues at the interface in EL2, KEVE/KDVE (*M. americana* Cx34.7 and Cx35), and one residue in EL1. The homologous residues in other connexins had been demonstrated to be highly tolerant to mutations and critical for docking specificity^57^. Mutations were modelled in Swiss PDB Viewer using homology models of Cx34.7 and Cx35 from a Cx26 and Cx32 interface structure so as not to create mutations with obvious steric hindrance. A wide range of substitutions were made for these five residues of interest, including those intended to introduce compatible electrostatic interactions, as well as less likely candidates. Mutations were also created that targeted other residues nearby and/or adjacent to these five for which there was some published evidence that they contributed to docking specificity. However, our semi-rational approach was such that not as many variants were evaluated for these more distal site mutations; mutations made in those sites were more conservative with regards to the steric and electrostatic properties of the change.

### Construct cloning and preparation

The initially acquired *M. americana* Cx34.7 and Cx35 cDNA constructs did not express efficiently in HEK293FT cells. Thus, connexin gene information was procured from the National Center for Biotechnology Information (NCBI) and the Ensembl genome browser. The human codon-optimized genes were ordered from Integrated DNA Technology (IDT) as gBlocks Gene Fragments. To generate constructs for transient transfection of HEK293FT cells, genes were subcloned into BamHI-digested and SacI-digested mEmerald-N1 (Addgene, 53976) and piRFP670-N1 (Addgene, 45457) vectors using In-Fusion cloning (Takara Bio), which resulted in connexin fluorescent fusion proteins, specifically with the fluorescent proteins adjoined to the connexin carboxy terminus. Mutant constructs were generated by using overlapping primers in standard Phusion polymerase PCR reactions to facilitate site-directed mutagenesis.

The Gateway recombination (Invitrogen) system was used to generate all Cx36, Cx34.7, Cx35, WT and mutant protein *C. elegans* expression plasmids. For PCR-based cloning and subcloning of components into the Gateway system, either Phusion or Q5 High-Fidelity DNA polymerase (NEB) was used for amplification. All components were sequenced in the respective Gateway entry vector before combining components into expression plasmids via a four-component Gateway system^58^. The different connexin versions were introduced into pDONR221a using a similar PCR-based strategy from plasmid sources^8,59,60^. Cell-specific promoters were introduced using the pENTR 50-TOPO vector (Invitrogen) after amplification from genomic DNA or precursor plasmids. Transgenic lines were created by microinjection into the distal gonad syncytium^61^ and selected on the basis of expression of one or more co-injection markers: Punc-122::GFP or Pelt-7::mCherry::NLS.

### Cell culture

HEK293FT cells were purchased from Thermo Fisher Scientific (R70007) and were maintained according to the manufacturer’s instructions. In brief, cultures were grown in 10-cm tissue culture-treated dishes in high-glucose DMEM (Sigma Aldrich, D5796) supplemented with 6 mM l-glutamine, 0.1 mM MEM non-essential amino acids and 1 mM MEM sodium pyruvate in a 5% CO_2_, 37 °C incubator. Cells were passaged by trypsinization every 2–3 days or until 60–80% confluency was reached. The cell line identity was not validated beyond the manufacturer’s certification of authenticity, and it was not tested for mycoplasma contamination. HEK293FT cells are not included in the list of commonly misidentified cell lines published by the International Cell Line Authentication Committee.

### Transient transfection

HEK293FT cells were plated in 10 μg ml^–1^ fibronectin-coated multiwell dishes to achieve about 75% confluency after overnight incubation. For transfection, 250 ng DNA was combined with polyethylenimine (PEI) diluted in Opti-MEM in a 1:3 ratio (DNA (µg) to PEI (µl)) and incubated at room temperature for 10 min. Following incubation, PEI–DNA complexes were added dropwise to wells of the plated cells. Treated cells were then incubated at 37 °C for 16–18 h, followed by a change in the medium. Expression of the connexin–fluorescent protein constructs were evaluated at 24 and 48 h after transfection by widefield or confocal microscopy and western blotting.

### FETCH

FETCH analysis is fundamentally a two-component system (Extended Data Fig. 1). To complete FETCH analysis, replica multiwell plates with HEK293FT cells were transfected with either of the two components being evaluated. The media of transfected wells were changed 16–18 h after transfection and cells were trypsinized. Next, HEK293FT cells expressing experimental connexin counterparts were combined. The entirety of combined samples was then plated onto new, 10 μg ml^–1^ fibronectin-coated wells of the same size, which resulted in hyperdensity and overconfluency. Following co-plating, samples were incubated for around 20–24 h, which allowed cells to make contacts and to potentially generate and internalize dually labelled gap junctions. Samples were then trypsinized, resuspended in PBS with 10 U ml^–1^ DNAse and fixed with paraformaldehyde (PFA; final concentration of 1.5%). Co-plated samples in 96-well plates were resuspended to a final volume of around 150 µl, whereas samples from 24-well plates were resuspended to a final volume of about 600 µl.

Flow cytometry data were collected on a BD FACSCanto II (2-colour experiments and high-throughput 96-well plates; 488 nm and 633 nm lasers), which uses BD FACSDiVa software. Samples were analysed in two selection gates before evaluation of fluorescence. First, presumable HEK293FT cells were identified by evaluating sample forward versus side scatter area. Next, single cells were selected by evaluating cells that maintained a linear correlation of forward scatter height to forward scatter area. Finally, the fluorescence profiles of each sample were generated.

### Automated FETCH output processing pipeline

Each FETCH experiment produces *.fcs files that contain all the channel data for fluorescence in the sample. Our automated pipeline loads these files and extracts forward scatter-area (FSC-A), side scatter-area (SSC-A) and forward scatter-height (FSC-H). Depending on the machine used, we either loaded the green channel as 1-A or as FITC-A. For the red channel, we had two options (APC-A (iRFP670) and PE-A (mApple)) or just one (5-A(iRFP670)). Next, our code produces two matrices containing SSC-A with FSC-A, and FSC-A with FSC-H, respectively.

Our first gate was drawn on the FSC-A versus SSC-A axes to exclude cellular debris that clustered in the lower left corner and the cells that saturated the laser (at the maximum of both axes). On a FSC-A versus SSC-A plot, the cellular debris usually is smoothly transitioning into the population of intact cells; therefore, we used a Gaussian kernel density estimator with the estimator bandwidth selection defined by the Scott’s rule to draw contours around the data in the SSC-A versus FSC-A matrix. We next used a set of heuristics to determine which of the contour lines should be used to define the first gate. Specifically, cellular debris usually clusters below 25,000 on both axes; therefore, any contour that included values at or below this value was excluded. Similarly, any contour within 1,000 of the maximum value of each axis was also excluded. Of the remaining contours, the largest one was selected and an oval equation was fitted to the points defining that contour to attenuate occasional protrusions that tap into the cellular debris population in rare cases. The fitted oval became the first gate.

For all the elements inside the first gate, a second gate was drawn in the axis of FSC-A and FSC-H to exclude non-single cells. For the second gate, first we fit a line to all the points. Next, for each point we identified a norm to the fit line and a standard deviation of all such norms. Using this standard deviation, we defined a second gate that was four standard deviations away from the fitted line on both sides and excluded all the points outside this gate (Extended Data Fig. 1c).

After applying the first two gates, we plotted the data with the red fluorophore on the *y* axis and the green fluorophore on the *x* axis. If a sample contained more than two fluorescent signals, the last gate was drawn for each possible combination. As some readings were below zero owing to fluorescence compensation, we shifted all the data points by the smallest value along both axes and then took a natural log of fluorescence levels. To achieve the optimal bandwidth for the kernel density estimation, we ran a cross-validation grid search algorithm on the points in the log space. Then we fit a Gaussian kernel density with the bandwidth estimated to obtain density contours. For properly expressing samples, we expected a large population of non-transfected cells in the bottom left quadrant of the plot, a population of cells strongly expressing the red fluorophore along the *y* axis and a population of cells strongly expressing the green fluorophore along the *x* axis (Extended Data Fig. 1d). We anticipated that the autofluorescence does not exceed 500 on either axis; therefore, the non-transfected population was defined to be below this value along both axes. To draw a tighter bound on the non-transfected population, we chose the first contour for which the mean kernel density estimate (k.d.e.) value was at or above the 60th quantile (identified as a generalizable heuristic value) of the distribution of k.d.e. values in the largest contour (at or below the autofluorescence cutoff). The top-most point of the tight contour defined the horizontal gate and the right-most point as the vertical gate, thereby separating the plot into four quadrants.

The upper-left quadrant Q1 corresponded to the cells expressing just the red fluorophore, the upper-right quadrant Q2 represented dual-coloured cells, the lower-right quadrant Q3 the cells expressing just the green fluorophore and the lower-left quadrant Q4 represented non-transfected population. The FETCH score is defined as the proportion of transfected cells that were dual-coloured: 100 × (Q2/(Q1 + Q2 + Q3)).

As we were expecting approximately equal expression levels of each fluorophore, if the number of cells in Q1 was two or more times larger or smaller than the number of cells in Q3, the FETCH score was classified as ‘dubious’ and marked accordingly in the output table. The ‘dubious’ label was also given to samples that had fewer than 500 cells total after the application of the second gate and to the samples that failed at any of the steps in the pipeline (usually due to poor expression or the absence of cells in the sample). The code for this pipeline is available from GitHub (https://github.com/carlson-lab/FETCH).

### In vitro screening of Cx34.7 and Cx35 mutants for docking selectivity

For homotypic docking screening analysis, five FETCH replicates were obtained for each mutant. These scores were benchmarked against scores for Cx36 with Cx45 (FETCH = 1.2 ± 0.1%, *n* = 54 replicates). For our heterotypic docking screening analysis, five replicates were obtained for each mutant pair. These scores were then benchmarked against scores for WT Cx34.7 with Cx35 (FETCH = 14.7 ± 0.4%, *n* = 49 replicates).

To quantitatively determine whether a connexin pair docked, we determined FETCH scores for the dual fluorescence of cells under conditions in which docking was not anticipated. These conditions included pairs of connexins previously established to not show docking (Cx36 and Cx45 (FETCH = 0.7 ± 0.0%, *n* = 59 replicates), homotypic Cx23 (FETCH = 0.9 ± 0.4%, *n* = 6), Cx36 and Cx43 (FETCH = 1.2 ± 0.2%, *n* = 10)) and under conditions for which cells were transfected with cytoplasmic fluorophores rather than tagged connexins (FETCH = 4.4 ± 0.6%, *n* = 17). These 92 FETCH scores were used as the known-negative distribution. FETCH scores from each experimental condition were then compared against the known-negative distribution using a one-tailed *t*-test, with an *α* threshold that was Bonferroni-corrected for the total number of experimental conditions tested (*n* = 21, producing an *α* = 0.05/21). These FETCH replicates were independent of the replicates used for our screening analysis. Statistics are reported as mean ± s.e.m. values, and only uncorrected *P* values are reported throughout.

### Confocal imaging analysis of gap junction partners

For imaging of putative gap junction partners, different populations of HEK293FT cells were transfected with counterpart connexin proteins, incubated and combined as described for FETCH analysis. Combined samples of HEK293FT cells were plated onto 10 μg ml^–1^ fibronectin-coated 35-mm, glass-bottom Mattek dishes (P35GC-1.5-14-C). Cells were imaged at about 20 h after plating. Images were acquired on a Leica SP5 laser point-scanning inverted confocal microscope using Argon/2, HeNe 594 nm and HeNe633nm lasers, conventional fluorescence filters and a ×63, HCX PL APO W Corr CS, NA: 1.2, water, DIC, WD: 0.22 mm, objective. Images were taken with 1,024 × 1,024-pixel resolution at a 200 Hz frame rate.

For assessing Cx34.7(M1)–Cx35(M1) expression in vivo in *C. elegans*, we imaged strain DCR8669 *olaEx5214* (*Pgcy-8::Cx34.7(E214K,E223K)::GFP;Pttx-3::Cx35(K221E)::mCherry;Punc-122::GFP*). Animals at the L4 stage were mounted in 2% agarose in M9 buffer pads and anaesthetized with 10 mM levamisol (Sigma). Confocal images were acquired using dual Hamamatsu ORCA-FUSIONBT SCMOS cameras on a Nikon Ti2-E inverted microscope using a confocal spinning disk CSU-W1 system, 488 nm and 561 nm laser lines and a CFI SR HP PLAN APO LAMBDA S ×100 C SIL objective. Images were captured using NIS-ELEMENTS software, with 2,048 × 2,048-pixel resolution, 16-bit depth, 300 nm step size, 200 ms of exposure time and enough sections to cover the entire depth of the worm.

### Protein modelling pipeline

Our protein modelling pipeline is based on a previously published methodology^34^ and integrates five components: (1) homology model generation; (2) embedding of proteins in a lipid bilayer and aqueous solution; (3) protein mutagenesis; (4) short-timescale energy minimization protocol incorporating explicit solvent and bilayer effects; and (5) residue-wise energy calculation. The code for our model is available from GitHub (https://github.com/carlson-lab/VMD-and-NAMD-Connexin-Protein-Simulation-Protocol).

#### Homology modelling

We initially tested five homology modelling software suites: Robetta, SwissModel, Molecular Operating Environment (MOE; Chemical Computing Group, H3A 2R7, 2021), I-Tasser and Phyre2 (refs. ^62–70^). A quality assessment suite, MolProbity^71–73^, revealed that Robetta models outperformed the rest, on the basis of a set of standard metrics (Ramachandran plot outliers, clashscore, poor rotamers, bad bonds or angles, among others). As our aim was to model the extracellular loops responsible for connexin hemichannel docking, we picked all the resolved connexin structures that possessed a high degree of extracellular loop homology to our connexin of interest as the inputs for Robetta. The top homologue hits were generally the same for the three connexins of interest: Cx26 bound to calcium (Protein Data Bank (PDB) ID: 5ER7.1)^74^; human Cx26 (calcium-free) (PDB ID: 5ERA)^74^; structure of Cx46 intercellular gap junction channel at 3.4 Å resolution by cryogenic electron microscopy (PDB ID: 6MHQ)^33^; structure of Cx50 intercellular gap junction channel at 3.4 Å resolution by cryogenic electron microscopy (PDB ID: 6MHY)^33^; and structure of the Cx26 gap junction channel at 3.5 Å resolution (PDB ID: 2ZW3)^56^. The Cx34.7 and Cx35 WT sequences had the greatest homology degree with the 6MHQ structure, whereas Cx36 was most homologous to 5ER7.1. We generated three WT hemichannels for Cx34.7, Cx35 and Cx36.

#### System assembly

Next, we assembled hemichannels into homotypic and heterotypic gap junctions, embedded them in two palmitoyl-oleoyl-phosphatidylcholine membrane double bilayers, solvated them in water and added appropriate ion concentrations for the extracellular and two intracellular compartments. The primary software suite used for this modelling step was VMD^75,76^. We also used CHARMM GUI to generate the naturalistic model of a region of a double bilayer^77–81^. Membrane components were then selected in appropriate proportions to resemble experimentally derived data from a neuronal axonal membrane.

Specifically, as Robetta was unable to model the full gap junction, we merged hemichannels into full homotypic or heterotypic gap junctions in a semi-automated way. First, to make homotypic gap junctions, we loaded the two homology models for a hemichannel. We then aligned them using the centre of mass of the extracellular loops. To make heterotypic gap junctions, we created a homotypic gap junction for each hemichannel, aligned the extracellular loops for the two homotypic gap junctions, then removed an opposing hemichannel from each homotypic gap junction (leaving the two different hemichannels aligned). Next, using the constructed gap junction, we aligned two pre-made membrane bilayers with the centre of mass assigned as each embedded hemichannel. We then removed membrane molecules that overlapped the hemichannel or the hemichannel pore. Next, we solvated the system in water and removed water that overlapped the lipid bilayer. Extracellular water was then separated to a new file, where Na^+^ ions [130 mM], K^+^ ions [5 mM], Cl^–^ ions [150 mM] and Ca^2+^ ions [2 mM] were added to produce concentrations mirroring the extracellular environment of mammalian neurons^82^. Finally, Na^+^ ions [12 mM], K^+^ ions [125 mM], Cl^–^ ions [10 mM] and Ca^2+^ ions [0.0001 mM] were added to the intracellular space to mirror the intracellular environment of mammalian neurons, and the files containing the embedded connexin hemichannels and extracellular water were merged, which generated solvated hemichannels at a pH of 7.2–7.4. Notably, these stages were automated to produce a streamlined progression from a protein-only hemichannel model to a fully embedded gap junction model ready for subsequent simulation and/or mutagenesis.

#### Mutagenesis

We developed a Python command-line tool that uses VMD to generate mutation configuration files for subsequent molecular dynamics simulation. Here we simply specified the connexin hemichannels of interest and the position at which a specific mutation should be introduced.

#### Short-timescale energy minimization protocol incorporating explicit solvent and bilayer effects

Next, we minimized atomic energies, equilibrated the system and ran the stable system in a production simulation run. Specifically, molecular dynamics simulation was performed using NAMD^83^ and a CHARMM36 force field. Our approach was divided into four steps:

1. Melt lipid tails while keeping remaining atoms fixed (simulate for 0.5 ns, NPT, 300 K).
2. Minimize the system (NVT, 300 K), then allow the bilayers and solutions to take a natural conformation while keeping the gap junction fixed (split in two stages to accommodate the reduction in volume of the relaxing system; simulate for 0.5 ns in total, NVT, 300 K, with harmonic positional restraints on the protein).
3. Release the gap junction and equilibrate the whole system (simulate for 0.6 ns, NVT, 310 K, 1 atm, using Langevin dynamics).
4. Run the minimized and equilibrated system in a production run (simulate for 0.5 ns, 1-fs step, NPT, 310 K).

Although molecular dynamics simulation (step 4) is highly reliant on the input file provided by the System Assembly process, these steps render the simulation more robust to modelling imperfections. For example, the membrane model developed though System Assembly is rigid and has the potential to behave like a solid rather than like a liquid. Thus, melting the lipid tails encourages the model to embody a liquid. Similarly, many atoms in the input file may have unnatural initial energies, such that if they are all released at once, they would start moving at high velocities and the simulation would fail. Therefore, bringing the system to a local energy minimum increases stability. Removing constraints on the water and lipids enables them to surround the gap junction in a naturalistic form. Finally, releasing the constraints on the gap junction enables it to take the most energetically stable conformation given the environment.

#### Energy calculation

To predict the residues that play a prominent part in docking, we quantified all non-bonding interactions between the two connexin hemichannels at key residues on the extracellular loops. Output from the molecular dynamic simulation was loaded into the VMD NAMD Energy plugin. We then calculated non-bonding energies for all residues on each hemichannel that were within 12 Å from at least one residue on the other hemichannel. For each residue pair, we then averaged energies across the 250 simulation frames.

### Characterizing gap junction biophysical properties using *Xenopus* oocytes

DNA sequences encoding Cx34.7(WT)-mEmerald, Cx34.7(M1)-mEmerald, Cx35(WT)-mApple and Cx35(M1)-mApple were cut out from the plasmids used for our mouse studies (see below) using BamH1 and ECoR1 and then cloned into the pXMX_T3(+) *Xenopus* oocyte expression vector at identical restriction sites. The newly generated plasmids were linearized by cutting with NgoMIV to serve as templates for in vitro synthesis of complementary RNAs (cRNAs). Capped cRNAs were synthesized using a mMessage mMachine T3 Transcription kit (AM1348, ThermoFisher Scientific). Each cRNA injection solution was a mixture of a specific cRNA and a *Cx38* antisense oligonucleotide (5′-GCTTTAGTAATTCCCATCCTGCCATGTTTC-3′, 100 ng µl^–1^).

In preliminary experiments with *Cx34.7*^*WT*^ and *Cx35*^*WT*^ cRNAs injected at an identical concentration (900 ng µl^–1^), we observed much larger *I*_j_ values in a homotypic gap junction of Cx35(WT) than one of Cx34.7(WT) under identical experimental conditions. Moreover, there was a larger cytotoxic effect (oocytes dying) with Cx35(WT) but not Cx34.7(WT). Therefore, in the final injection solutions used for the main experiments presented in this study, the concentrations of *Cx34.7*^*WT*^*-mEmerald* and *Cx34.7*^*M1*^-*mEmerald* cRNAs were 900 ng µl^–1^, whereas those of *Cx35*^*WT*^*-mApple* and *Cx35*^*M1*^*-mApple* cRNAs were 90 ng µl^–1^. Approximately 50.6 nl of the mixture was injected per oocyte using a Drummond Nanoject II injector (Drummond Scientific). Injected oocytes were incubated in ND96 solution (96 mM NaCl, 2 mM KCl, 1.8 mM CaCl_2_, 1 mM MgCl_2_ and 5 mM HEPES (pH 7.5)) inside an environmental chamber (15 °C) before being paired. Oocytes were paired 20–24 h after *Cx35*^*WT*^ and *Cx35*^*M1*^ cRNA injections, but 68–72 h after *Cx34.7*^*WT*^ and *Cx34.7*^*M1*^ cRNA injections. To closely match experimental conditions between related groups, oocytes from one frog were used to analyse homotypic gap junctions of Cx34.7(WT) and Cx34.7(M1), whereas oocytes from another frog were used to examine homotypic gap junctions of Cx35(WT) and Cx35(M1). Furthermore, an identical batch of oocytes from a third frog was used to investigate heterotypic gap junctions of both Cx34.7(WT)–Cx35(WT) and Cx34.7(M1)–Cx35(M1). The vitelline membrane of the oocyte was removed using a pair of no. 5 Dumont tweezers (500342, Word Precision Instruments) during the pairing process. Paired oocytes were incubated in ND96 solution for 12–24 h at room temperature (20–22 °C) before electrophysiological recordings. Voltage–clamp recordings were performed using two Oocyte Clamp amplifiers (OC-725C, Warner Instruments) in the high-side current measuring mode. In the electrophysiological experiments, we applied a series of membrane voltage steps (–150 mV to +90 mV at 10-mV intervals, 7 s in duration) to one oocyte from a holding voltage of −30 mV while holding the other oocyte constant at −30 mV to record transjunctional currents (*I*_j_). A pre-pulse (–30 mV) was applied from the holding voltage before each voltage step so that the stability of the recordings could be monitored by the induced *I*_j_. Instantaneous current (*I*_inst_) and steady-state current (*I*_ss_) were defined as the peak *I*_j_ at the beginning of each *V*_j_ step and as the averaged *I*_j_ during the last 2 s of the *V*_j_ step, respectively. Details of the experimental and data analysis procedures can be found in our previous publications^35,36^.

### Generation and validation of *Cx43* and *Cx45* double-knockout HEK293FT cells

Connexin double-knockout HEK293FT cells were produced by the Duke Functional Genomics Core Facility using CRISPR–Cas9 technology. Specifically, insertion and/or deletion mutations were introduced via CRISPR–Cas9 in the *Cx43* and *Cx45* sequences to induce frameshifts in the *Cx43* (*GJA1*) and *Cx45* (*GJC1*) DNA sequence, which results in premature translation termination (Extended Data Fig. 5a–c). Specific mutations were identified through DNA sequencing and evaluation of sequence chromatograms (exported as encapsulated postscript (.eps) files) in ApE Plasmid editor^84^. To confirm *Cx43* and *Cx45* knockout, three double-knockout HEK293FT monoclonal cell lines were isolated and expanded for evaluation by western blotting. Cells were collected in ice-cold PBS (Gibco, 10010031) enriched with protease and phosphatase inhibitors 1:100 (Sigma-Aldrich, P8340 and P5726). Cells were then centrifugated at 1,000*g* for 5 min at 4 °C to remove PBS, and the pellet was sonicated twice for 30 s (20% output power) in ice-cold lysis buffer (Phosphosolutions, NC1671658) enriched with protease and phosphatase inhibitors 1:100 (Sigma-Aldrich, P8340 and P5726). Cell lysates were kept on ice for 30 min and then centrifugated at 14,000*g* for 30 min at 4 °C. Lysate supernatants were collected, and total protein concentrations were measured using a bicinchoninic acid assay (Thermo Fisher Scientific, 23228 and 1859078). Next, 40 μg of total protein was denatured at 95 °C for 5 min in LDS sample buffer (7 µl per sample) (Thermo Fisher Scientific, NP0007) and sample reducing agent (3 µl per sample) (Thermo Fisher Scientific, NP0009). The denatured samples were then separated using Novex Tris-Glycine gels 4–20% (Thermo Fisher Scientific, XP04200BOX) and electrophoresis at 125 V for 10 min and 150 V for around 1 h, then transferred to polyvinylidene fluoride membranes (Thermo Fisher Scientific, 88520) overnight at 0.04 A. Membranes were blocked with 5% dry non-fat milk (Blotting-Grade Blocker, Bio-Rad, 1706404) in PBST (0.1% Tween-20) for at least 2 h at room temperature and next incubated overnight at 4 °C with the following antibodies in blocking solution: anti-Cx43 (Cell Signaling Technologies, 3512, 1:1,000) and anti-Cx45 (Abcam, ab316742, 1:1,000). GAPDH was measured after the protein of interest (Cx43 or Cx45) as control (anti-GAPDH, Abcam, ab181602, 1:5,000). After incubation with the primary antibody, this was removed and blots were washed in PBST 0.1% (3 washes, 15 min each) at room temperature, following incubation with horseradish-peroxidase-labelled secondary anti-rabbit IgG antibody (Cell Signaling Technologies, 7074) for 1 h at room temperature. Membranes were washed in PBST 0.1% to remove the secondary antibody (3 washes, 15 min each). Immunoreactive bands were visualized with SuperSignal West Pico PLUS Chemiluminescent substrate (Thermo Fisher Scientific, 34578) and detected using a ChemiDoc Imaging system (Bio-Rad, 12003153). Bands were analysed with ImageJ. For each protein studied, the ratio was calculated by dividing the densitometric value of the protein of interest by the correspondent GAPDH value × 100 (Extended Data Fig. 5).

### PCR confirmation of *Cx43* and *Cx45* knockouts

Genomic DNA from HEK293FT double-knockout cell line clones of 405, 412 and 422 was extracted using a KAPA NGS DNA extraction kit (Roche, 09189823001), and PCR was performed using Invitrogen Platinum super Fi PCR master mix (12358010) following the manufacturer’s instructions. PCR products were prepared by adding a DNA stain (APEBIO, ab743) and then separated in 1% agarose gel (Bio-Rad, 1613102) in TAE buffer (Bio-Rad, 1610743) at 100 V for about 30 min. DNA fragments were detected using a ChemiDoc Imaging system (Bio-Rad, 12003153).

### WT and connexin double-knockout HEK293FT cultures for patching experiments

WT HEK293FT cells were purchased from Thermo Fisher Scientific (R70007). Connexin double-knockout HEK293FT cells were generated as described above. Cells were maintained according to the manufacturer’s instructions as noted above.

### Glass coverslip preparation

In brief, 12-mm German glass coverslips (NeuVitro, GG-12-15-fibronectin) were first pretreated with acetone for 20 min with shaking. Next, they were washed 3 times for 10 min in 70% ethanol with shaking and stored in 70% ethanol before use. Once needed, each coverslip was washed 6 times with cell-culture-grade deionized water to remove any ethanol remnants. For the patching experiments, coverslips were then coated using 1:100 fibronectin (Sigma-Aldrich, F1141-2MG) (stock at 1 mg ml^–1^) in poly-d-lysine (Gibco, A38904-01) (stock at 0.1 mg ml^–1^ in PBS) for 1 h at room temperature. After coating, the solution was aspirated and the coverslips were left to dry in a cell culture hood, uncovered, for 1–2 h.

### Sample preparation for paired cell recordings

For the recording experiments using non-transfected cells, HEK293FT or connexin double-knockout HEK293FT cells were grown in 10 cm tissue-culture-treated dishes and seeded onto the coverslips, at the same density conditions used for all patching experiments (35,000 cells per well in total), the day before the recording. To evaluate connexin proteins, connexin double-knockout HEK293FT cells were seeded with no antibiotic cell culture medium, and the next day they were transfected using Lipofectamine 2000 transfection reagent (Thermo Fisher, 11668027) at a Lipofectamine 2000 to DNA ratio of 2.5–1.9:1. (0.8–1 μg cDNA to 1.9–2 µl Lipofectamine). Opti-MEM (Gibco, 31985070) medium was used following the Lipofectamine 2000 protocol. Medium was changed after 4 h to remove the transfection reagent. For each patching condition, 4 or 20 h after transfection, cells were detached, counted and re-seeded in equal amounts of cells from each fluorescent condition (35,000 cells per well in total) onto a precoated glass coverslip in a 24-well plate (see the section above for coverslip preparation). The cells were left to form gap junctions overnight and recordings were conducted the following day. These patching experiments were performed using the construct cloning and preparation procedures described above, with minor changes. In brief, for the Cx34.7(M1)–Cx35(M1) and Cx36–Cx36 patching conditions tested, cells expressed either mEmerald or mCherry. For 2 out of the 23 connexin double-knockout HEK293FT negative control experiments, we used cell pairs in which one cell expressed mEmerald and the other expressed mCherry. Finally, we used only one fluorescent tag for the Cx34.7(M1)–Cx34.7(M1) or Cx35(M1)–Cx35(M1) experiments (mEmerald and mCherry respectively).

### Paired HEK293FT cell recordings

Glass coverslips with adhered WT or connexin double-knockout HEK293FT cells were placed into a recording chamber on an upright fluorescent microscope (Scientifica). The chamber was filled with extracellular solution (ECS) at room temperature (21–24 °C). The ECS contained the following factors (in mM): 5.4 KCl, 1 CaCl_2_, 140 NaCl, 5 HEPES, 1.8 MgCl_2_ and 5 glucose; pH was adjusted to 7.4 with NaOH, with an osmolarity of 310–320 mOsm. Patch pipettes were pulled using a micropipette puller (Sutter Instruments P1000) and filled with intracellular solution containing the following factors (in mM): 135 CsCl, 0.5 CaCl_2_, 2 MgCl_2_, 5.5 EGTA, 5 HEPES, 3 MgATP, 2 Na_2_ATP; pH was adjusted to 7.2 with CsOH, with an osmolarity of 290 mOsm. The patch pipette resistance was in the range of 2–4 MΩ. Isolated cell pairs were selected for dual whole-cell voltage clamp experiments.

To study transjunctional currents between cell pairs, the following protocol was applied in voltage clamp. A series of square voltage pulses (stepped up from −160 mV to +70 mV in 10-mV increments) was alternated between the two cells. While the first cell received the pulses, the second cell was held at −45 mV to measure the transjunctional current. This procedure was repeated for both cells.

### Analysis of transjunctional current

All current traces were filtered using Clampfit 11.4 (Molecular Devices). The baseline current was measured 500 ms before the voltage pulse. The current for each 200 ms voltage step was then determined as the difference between the current recorded during the last 50 ms of each step and this baseline current. The relationship between this current and transjunctional voltage was determined for each cell using Pearson correlation analysis, and the values were averaged across cells in a cell pair (Extended Data Fig. 6). The current at the +70 mV step was extracted for each cell and averaged across the pair to determine the maximum transjunctional current used for analysis.

To test Cx36, we plated cells expressing red or green fluorescent proteins. Because Cx36 shows homotypic docking, we patched fluorescent cell pairs irrespective of their colour. To test Cx34.7(M1)–Cx35(M1), we selected and patched pairs for which one of the cells was clearly labelled by green fluorescent protein and the other cell was labelled by red fluorescent protein. We also selected and patched pairs for which both cells were labelled by both fluorophores. Visual inspection at multiple views enabled us to separate out these experimental pairs on the basis of high confidence that one of the cells in a pair was not labelled by both green and red fluorescence (Extended Data Fig. 6). To test Cx34.7(M1) or Cx35(M1), we patched cell pairs expressing a single fluorophore.

### Statistical analysis of HEK293FT whole-cell recording data

We compared untransfected WT HEK293FT cell pairs to connexin double-knockout HEK293FT cell pairs. Next, the maximum transjunctional voltage recorded from connexin double-knockout HEK293FT cell pairs transfected with connexins was compared with that from a connexin double-knockout HEK293FT cell pair control group using a one-tailed Wilcoxon rank-sum test, followed by a FDR correction. We chose one-tailed testing here because we were specifically evaluating whether the expression of our mutant proteins increased current above the baseline current observed in the connexin double-knockout HEK293FT cell pairs. Notably, in contrast to the published methodology using HEK293 cells^37^, knocking down *Cx43* and *Cx45* did not completely abolish electrical coupling between HEK293FT cells. Indeed, in our preliminary experiments, we observed electrical coupling in 1 out of 10 connexin double-knockout HEK293FT cell pairs (as indicated by a transjunctional voltage × current relationship > *R*^2^ = 0.75, Pearson correlation). In our main experiments, we observed coupling in 6 out of 34 of our negative control pairs (5 out of 23 connexin double-knockout HEK293FT cell pairs and 1 out of 11 of the pairs that expressed Cx34.7(M1)–Cx35(M1) but did not exhibit dual fluorescence in both cells; Extended Data Fig. 6b). In our post hoc testing, we observed a significantly higher proportion of coupled cells in our two positive control groups (7 out of 10 Cx36–Cx36 cell pairs, *χ*^2^ = 10.2 and *P* = 0.001 compared with the pooled negative controls, chi-squared test; 7 out of 10 Cx34.7(M1)–Cx35(M1) that exhibited dual fluorescence in both cells, *χ*^2^ = 10.2 and *P* = 0.001). These findings increased our confidence that this mammalian cell system could be used to test Cx34.7(M1)–Cx34.7(M1) and Cx35(M)1–Cx35(M1) coupling. Indeed, no coupling was observed in the Cx34.7(M1)–Cx34.7(M1) group (0 out of 10 pairs tested), and the coupling observed in the Cx35(M1)–Cx35(M1) groups was statistically indistinguishable from the pooled negative control group (3 out of 10 Cx35(M1)–Cx35(M1) cell pairs, *χ*^2^ = 0.7 and *P* = 0.40).

### *C. elegans* strains and genetics

Nematodes were maintained at 20 °C on NGM plates seeded with a lawn of *Escherichia coli* strain OP50 using standard methods^85^. All worm experiments were performed using 1-day-old adult hermaphrodites. The strains used in this study are listed in Supplementary Table 2. All thermotaxis behavioural assays, calcium imaging experiments and generation of transgenic lines were performed as previously described^10^, with minor modifications as outlined in the following subsections.

### Thermotaxis behavioural assay

Animals were grown and assayed as previously described^10,86^. In brief, after being reared at 20 °C, animals were trained at 15 °C for 4 h before testing. Their migration tracks were analysed as previously described^10,86,87^. Each behavioural arena was split in half along the temperature gradient using a thin and clear plastic divider. This enabled WT controls to be assayed on one half of the arena and connexin-expressing animals on the other half simultaneously.

### Calcium imaging in *C. elegans*

Imaging calcium dynamics for assaying AFD–AIY functional coupling was performed as previously described^10^, with the following modifications: a Leica DM6B was used instead of Leica DM5500, and image acquisition was performed using MicroManager^88^. Segmentation into regions of interest (ROIs) and downstream data processing were performed using Fiji^89^ and custom scripts written in Matlab (Matlab, v.2021A-2024B, MathWorks) were used as previously detailed^10^. For analyses of AFD calcium transients, we generated and measured a ROI around a single AFD soma per animal. For analyses of AIY calcium dynamics, we generated and quantified a ROI at the synaptic subcellular region known as zone 2 (ref. ^90^). Responses were scored as the initial rise of the AFD or AIY calcium signal as determined by a human observer blinded to the experimental conditions. The genetic background for the AFD and some of the AIY calcium imaging lines used in this study (control and experimental) contained *olaIs23*, a *caPKC-1* GOF mutation. This was done to match previous work^10^ in which Cx36 was demonstrated to evoke AFD-locked responses in AIY compared with *caPKC-1* animals without Cx36.

### Vertebrate animal care and use

Male B6.129P2-*Pvalb*^*tm1(cre)Arbr*^/J (PV-Cre mice) and female C57BL/6J mice purchased from The Jackson Laboratory (strain 017320 and 000664, respectively) were bred to generate the male and female (*n* = 28 total virally injected and implanted mice) PV-Cre heterozygous mice subjected to the prefrontal cortex PYR–PV^+^ editing experiment quantifying LFP coupling. Male C57BL/6J mice (*n* = 29) purchased from the Jackson Laboratory (strain no 000664) were used for non-edited controls. Mice were housed at a density of three to five mice per cage on a 12-h light–dark cycle and were maintained in a room with controlled humidity (30–70%) and temperature (22.7 ± 1.8 °C), with water and food available ad libitum. Neural recordings were conducted during the dark cycle (Zeitgeber time: 13–19) given previous evidence that electrical synapse conductance can be reduced in the retina via circadian regulation^91^. PV-Cre mice were crossed with B6;129S-*Slc17a6*^*tm1.1(flpo)Hze*^/J mice purchased from The Jackson Laboratory (stock no 030212) to obtain the mice used for PV-Cre/VGLUT2-flp mice subjected to the prefrontal cortex PYR–PV^+^ editing experiment quantifying cellular coupling. Groups were balanced by age and sex. In-bred BALB/cJ male mice (*n* = 56) purchased from The Jackson Laboratory (strain 000651) were used for IL→MD circuit editing experiments. We chose this strain and sex of mice to mirror our previous study that optogenetically targeted the IL→MD circuit^50^. Behavioural and physiological experiments were conducted during the dark cycle (Zeitgeber time: 13–22). All vertebrate animal studies were conducted with approved protocols from the Duke University Institutional Animal Care and Use Committees and were in accordance with the NIH guidelines for the Care and Use of Laboratory Animals.

### Generation of mouse viral constructs

The mouse codon-optimized WT and mutant connexin genes were ordered from IDT as gBlocks Gene Fragments. To generate fluorescently tagged connexin constructs for viral transformation of mouse neurons, gBlocks were ligated into BamHI and EcoRI digested pAAV-CaMKIIa-eGFP (Addgene, 50469). To generate fluorescently tagged connexin constructs for viral transformation of Cre-expressing neurons, connexin constructs were amplified from aforementioned CaMKIIa constructs and ligated into AscI-digested and NheI-digested pAAV-Ef1a-DIO-EYFP (Addgene, 27056). Finally, to generate Flag-tagged connexin constructs with co-expressed cytoplasmic fluorescent proteins, a gBlock Gene Fragment of Cx35-Flag-T2A-mCherry was ordered from IDT, amplified and ligated into AscI and NheI digested pAAV-hSyn-DIO-eGFP (124; Addgene, 50457*). All ligations were accomplished using In-Fusion cloning (Takara Bio). AAV viruses were created by the Duke University Viral Vector Core.

### Mouse viral injection surgeries

For the PYR–PV^+^ interneuron microcircuit editing experiment using microwires, PV-Cre mice were anaesthetized with isoflurane (1%), placed in a stereotaxic device and injected with a 1:1 solution of AAV9-CaMKII-Cx34.7(M1)-mEmerald (titre: 5.0 × 10^12^ vector genomes (vg) per ml) and AAV9-Ef1α-DIO-Cx35(M1)-mApple (titre: 1.3 × 10^13^ vg per ml) on the basis of stereotaxic coordinates measured from bregma at the skull to target PrL bilaterally (2.1 mm anterior–posterior (AP), 0.65 mm medial–lateral (ML) and –1.45 mm dorsal–ventral (DV) from the dura at a 21° angle for male mice; or 2.05 mm AP, 0.62 mm ML and –1.41 mm DV from the dura at a 21° angle for female mice). A total of 1 µl viral solution was delivered to each hemisphere over 10 min using a 5 µl Hamilton syringe. This strategy induces the expression of Cx35(M1) solely by PV^+^ interneurons and nonselective expression of Cx34.7(M1). Control mice nonselectively expressed Cx34.7(M1) or Cx35(M1). In these mice, we injected with a 1:1 solution of AAV9-CaMKII-Cx34.7(M1)-mEmerald and AAV9-Ef1α-DIO-Cx34.7(M1)-mApple (titre: 1.1 × 10^13^ vg per ml) or a 1:1 solution of AAV9-CaMKII-Cx35(M1)-mEmerald (titre: 6.9 × 10^12^ vg per ml) and AAV9-Ef1α-DIO-Cx35(M1)-mApple, respectively, to mirror the injection conditions of the experimental group. All viruses were created by the Duke Viral Vector Core or purchased from Addgene. Viral injections were performed in male and female mice at age 2.5–5 months, and viral manipulations were balanced across cages and sex.

For the PYR–PV^+^ editing experiment using silicon probes, PV-Cre/VGLUT2-flp mice were anaesthetized with isoflurane (1%), placed in a stereotaxic device and injected with a 1:1 solution of AAV9-CaMKII-Cx34.7(M1)-mEmerald (titre: 1.4 × 10^13^ vg per ml) and AAV9-hsyn-DIO-Cx35(M1)-T2A-mCherry (titre: 1.3 × 10^14^ vg per ml) on the basis of stereotaxic coordinates measured from bregma at the skull to target the PrL bilaterally (1.8 mm AP, 0.3 mm ML, –2.85 mm and –2.5 mm DV from the skull for male mice; or 1.75 mm AP, 0.29 mm ML, –2.78 mm and –2.44 mm DV from the skull for female mice). A total of 0.3 µl viral solution was delivered to each DV target for each hemisphere over 10 min using a 5 µl Hamilton syringe. This strategy induced expression of Cx35(M1) solely by PV^+^ interneurons and nonselective expression of Cx34.7(M1). Control mice solely expressed fluorophores. In these mice, we injected with a 1:1 solution of AAV9-CaMKII-eGFP (titre: 2.8 × 10^13^ vg per ml) and AAV9-hSyn-DIO-mCherry (titre: 1.9 × 10^13^ vg per ml). All viruses were created by the Duke Viral Vector Core or purchased from Addgene. Viral injections were performed in four female and two male mice at age 2.5–5 months, and viral manipulations were balanced across cages and sex.

For the IL→MD circuit interrogation study, 3-month-old male BALB/cJ mice (*n* = 21) were injected with a 1:1 solution of AAV9-CaMKII-Cx34.7(M1)-mEmerald (titre: 2.3 × 10^13^ vg per ml) and AAV9-CamKII-ChR2-EYFP (titre: ≥1 × 10^13^ vg per ml) to target the left IL unilaterally (1.7 mm AP, 0.72 mm ML, measured from bregma; –2.03 mm DV from the dura at an angle of 10°). A total of 0.5 µl viral solution was delivered at a rate of 100 nl min^–1^ over 5 min using a Hamilton syringe. Three weeks after the first surgery, mice were again anaesthetized, placed in a stereotaxic device and injected with either AAV9-CaMKII-Cx35(M1)-mApple (titre: 3.16 × 10^13^ vg per ml) or AAV9-CaMKII-eGFP (titre: 2.3 × 10^13^ vg per ml) to target left MD unilaterally. A total of 0.5 µl viral solution was delivered. Viruses were infused at a rate of 100 nl min^–1^ over 5 min with a Hamilton syringe.

For the IL→MD behavioural experiment, 3-month-old male BALB/cJ mice (*n* = 16) were anaesthetized with isoflurane (1%), placed in a stereotaxic device and injected with AAV9-CaMKII-Cx34.7(M1)-mEmerald (titre: 5.0 × 10^12^ vg per ml) on the basis of stereotaxic coordinates measured from bregma at the skull to target the IL bilaterally (1.7 mm AP, ±0.72 mm ML, –2.03 mm DV from the dura at an angle of 10°). A total of 0.5 µl viral solution was delivered to each hemisphere over 5 min using a 5 µl Hamilton syringe, which was left in place for an additional 10 min before removal. Three weeks later (Extended Data Fig. 8d), mice were injected with AAV9-CaMKII-Cx35(M1)-mApple (titre: 6.9 × 10^12^ vg per ml) on the basis of stereotaxic coordinates measured from bregma at the skull to target the MD bilaterally (–1.58 mm AP, 0.5 mm ML, –2.88 mm DV from the dura at an angle of 10°). Control mice (*n* = 19) were injected with AAV9-CaMKII-Cx34.7(M1)-mEmerald in both the IL and MD, or with AAV9-CaMKII-Cx35(M1) in both the IL and MD, to express the mutant hemichannels in non-docking homotypic configurations.

### Electrode implantation surgery

For the PYR–PV+ interneuron microcircuit editing experiment, 29 PV-Cre mice were anaesthetized with isoflurane (1%), placed in a stereotaxic device and metal ground screws were secured to the cranium. A total of 8 tungsten microwires were implanted in the PrL (centred at 1.8 mm AP, ±0.25 mm ML and –1.75 mm DV from the dura for male mice; or centred at 1.76 mm AP, ±0.25 mm ML and –1.71 mm DV from the dura for female mice). C57BL/6J control mice were implanted at 2 months of age for another set of experiments outside the ones described here. A total of 32 tungsten microwires were arranged in our previously described multilimbic circuit recording design^92^. In brief, bundles were implanted to target basolateral and central amygdala (Amy), MD, nucleus accumbens core and shell (NAc), VTA, medial prefrontal cortex (mPFC) and ventral hippocampus (VHip) were centred on the basis of stereotaxic coordinates measured from bregma: Amy, –1.4 mm AP, 2.9 mm ML and –3.85 mm DV from the dura; MD, –1.58 mm AP, 0.3 mm ML and –2.88 mm DV from the dura; VTA, –3.5 mm AP, ±0.25 mm ML and –4.25 mm DV from the dura; VHip, –3.3 mm AP, 3.0 mm ML and –3.75 mm DV from the dura; mPFC, 1.62 mm AP, ±0.25 mm ML and 2.25 mm DV from the dura; NAc, 1.3 mm AP, 2.25 mm ML and –4.1 mm DV from the dura; all implanted at an angle of 22.1°. We targeted the cingulate cortex, PrL and IL using the mPFC bundle by building a 0.5 mm and 1.1 mm DV stagger into our electrode bundle microwires. Animals were implanted bilaterally in the mPFC and VTA. All other bundles were implanted in the left hemisphere. The NAc bundle included a 0.6 mm DV stagger such that wires were distributed across the NAc core and shell. We targeted basolateral amygdala (BLA) and central amygdala (CeA) by building a 0.5 mm ML stagger and 0.3 mm DV stagger into our AMY electrode bundle^92^.

For the IL→MD circuit interrogation study, 16 tungsten microwires were arranged into two bundles to target IL (8 wires) and MD (8 wires). The IL bundle was also built with an optical fibre (MFC_100/125-0.22_8.0mm_MF2.5_FLT, Doric Lenses) 0.5 mm above the tip of the wires as previously described^50^. An optic fibre was also built into the MD microwire bundle for two-thirds of the animals, although it was not used for this study. Mice were anaesthetized as described above, and metal ground screws were secured to the cranium. Bundles were implanted in left IL (1.7 mm AP, 0.15 mm ML, from bregma; –2.25 mm DV from the dura) and left MD (centred at –1.58 mm AP, 0.35 mm ML, –2.88 mm DV from the dura).

### Data acquisition for PrL PYR–PV^+^ interneuron microcircuit editing

Neural recording experiments were performed in PV-Cre mice (*n* = 29) 1 week after implantation surgery, and the experimenters were blinded to viral group. Mice were habituated to the recording room for at least 60 min before testing. PV-Cre mice were connected to a headstage (Blackrock Microsystems) without anaesthesia and given a single saline injection (10 ml kg^–1^ mouse, intraperitoneally). Notably, these saline injections were performed to facilitate comparison of acquired neural data with future drug studies. Then, 25 min later, mice were placed in a 44.45 × 44.45 × 29.85 cm (length × width× height (L × W× H)) chamber for 60 min. Recordings were conducted under low illumination conditions (1–2 lux), and only data from the first 10 min of exposure to the open field were used for neurophysiological analysis. For C57BL/6J control mice, experiments were performed at least 2 weeks following implantation surgery. Mice were habituated to the recording room for at least 60 min before testing, and headstages were connected without anaesthesia. Twenty-nine C57 male mice were placed in a 44.45 × 44.45 × 29.85 cm (L × W × H) chamber for 10 min, and 3 mice were recorded in a 49.53 × 30.48 cm (diameter × height) circular chamber. Six of these mice were injected with saline (10 ml kg^–1^ mouse, intraperitoneally) 30 min before recordings, and all recordings were conducted under an illumination of 125 lux.

Neuronal activity was sampled at 30 kHz using a Cerebus acquisition system (Blackrock Microsystems). LFPs were bandpass filtered at 0.5–250 Hz and stored at 1,000 Hz. All neurophysiological recordings were referenced to a ground wire connected to both ground screws, and an online noise cancellation algorithm was applied to reduce 60 Hz artefact.

### Determination of LFP cross-frequency phase coupling and spectral power

Signals recorded from all viable implanted microwires were used for analyses. LFPs were filtered using fourth-order Butterworth bandpass filters designed to isolate theta (4–10 Hz) prefrontal cortex oscillations and HFOs (80–200 Hz). The Matlab filtfilt function was used to minimize phase distortions. The instantaneous amplitude and phase of the filtered LFPs were then determined using the Hilbert transform, and the modulation index was calculated for each LFP channel using a previously published Matlab code^46^. In brief, a continuous variable *z*(*t*) is defined as a function of the instantaneous theta phase and instantaneous gamma amplitude such that *z*(*t*) = *A*_G_(*t*) × *e*^iϕ^_TH_^(*t*)^, where *A*_G_ is the instantaneous gamma oscillatory amplitude and *e*^iϕ^_TH_ is a function of the instantaneous theta oscillatory phase. A time lag is then introduced between the instantaneous HFO amplitude and theta phase values such that *z*_surr_ is parameterized by both time and the offset between the two variables, *z*_surr_ = *A*_HG_(*t* + *τ*) × *e*^iϕ^_TH_^(*t*)^. The modulus of the first moment of *z*(*t*), compared with the distribution of moduli for the surrogates, provides a measure of coupling strength. The normalized *z*-scored length, or modulation index, is then defined as *M*_NORM_ = (*M*_RAW _– *µ*)/*σ*, where *M*_RAW_ is the modulus of the first moment of *z*(*t*), *µ* is the mean of the surrogate lengths, and *σ* is their standard deviation^44,46,50^. The modulation index scores were averaged across all implanted channels for each mouse (around 7.3 channels for each PV-Cre mouse, and 2 channels implanted bilaterally for each C57BL/6J control mouse) to produce a single score per animal.

To quantify LFP oscillatory power, a sliding window Fourier transform with Hamming window was applied to the LFP signal using Matlab. Data were analysed with a 1-s window, 1-s step and a frequency resolution of 1 Hz. Signals were averaged across time windows and frequencies used for cross-frequency phase coupling analysis and then across all microwires to produce a single measure per animal.

### Determination of medial prefrontal cortex PV^+^ interneuron phase locking to local oscillations

Nine days following viral surgery, PV-Cre/VGLUT2-flp mice were anaesthetized with isoflurane (1%), placed in a stereotaxic device and metal ground screws were secured to the cranium. A 1,024-channel silicon probe (NeuroNexus, SINAPS_4S_1024) was implanted to target the medial prefrontal cortex (–1.7 mm AP in male mice or 1.65 mm AP in female mice, centred at the midline, measured from bregma; –4 mm DV from the dura). Individual shanks were spaced by 500 µm on the probe such that the medial 2 targeted the medial prefrontal cortex bilaterally at ±0.25 mm ML. All mice were pair-housed with one animal from the other experimental group. Following a 2-week recovery period, mice were placed in a new cage that contained bedding from their home cage, connected to a mezzanine board and headstage, and this new cage was placed in the recording arena. Mice were individually habituated to this arena during their light cycle, and each mouse was habituated to its own new home cage. After habituation, mice were returned to their pair housing in their original home cage. This habituation procedure was repeated during the dark cycle at least 3 times over the subsequent 2–4 days. On the recording day, mice were habituated to their second home cage for 10 min, connected to the mezzanine board and headstage, and their second home cage was placed in the recording arena. Recordings began after animals were habituated for an additional 10–15 min. Neural data were collected for at least 10 min. Two mice continued to audibly rustle the bedding after 15 min of habituation; therefore, we extended the habituation for another 5–10 min. For each mouse, the last 10 min of neural data, which corresponded to low activity periods, were used for subsequent electrophysiological analyses. We selected these periods as our previous work demonstrated that phase coupling is reduced during novelty exposure and exploration^44,93^.

Neural data were sampled at 20 kHz using a SmartBox Pro acquisition system (NeuroNexus). To extract single-unit activity, neural activity was converted to the Neurodata Without Borders (NWB) format, high-pass filtered at 300 Hz, and the medial 512 channels (two medial shanks) were automatically sorted using Kilosort4 (ref. ^94^). PV^+^ single units were identified on the basis of interspike interval (ISI) violations of <0.5, a presence ratio of >0.9, an amplitude cutoff of <0.1 (ref. ^95^ and https://allensdk.readthedocs.io/en/latest/_static/examples/nb/ecephys_quality_metrics.html), a peak to valley ratio of <1.1 and a mean firing rate >10 Hz (ref. ^47^). PYR single units were identified on the basis of ISI violations of <0.5, a presence ratio of >0.9, an amplitude cutoff of < 0.1 (ref. ^95^ and https://allensdk.readthedocs.io/en/latest/_static/examples/nb/ecephys_quality_metrics.html), a spike half-width of >250 µs and a mean firing rate of <20 Hz (ref. ^48^). Only single units that mapped to the medial prefrontal cortex (cingulate cortex, prelimbic cortex and IL) targeted channels (192 total PV^+^ interneurons (32 ± 5.4 per mouse) and 207 PYR neurons (34.5 ± 12.8 per mouse) across 6 mice) were used for analysis.

Only neuronal activity that occurred within the last 10 min of the recording was used to determine phase locking. This approach was taken to control for small differences in recording length across animals. LFP activity was high-pass filtered at 0.5 Hz using a first-order Butterworth filter and filtered again using a fourth-order Butterworth bandpass filter to isolate theta (4–10 Hz), gamma (30–80 Hz) and HFOs (80–200 Hz). The instantaneous phase of the filtered LFPs was then determined using the Hilbert transform, and phase locking was calculated using the Matlab circular statistics toolbox (the ‘circ_r’ function for the MRL and the ‘circ_rtest’ function for the Rayleigh test of uniformity analyses).

The cross-correlation between PYR–PV^+^ pair spike trains was determined using the Matlab ‘Xcorr’ function with normalization. Temporally shifted PV^+^ interneuron activity was compared with PYR neuron activity, and the maximum correlation strength was calculated within the 1–4 ms window after PYR neuron firing. To test for significant coupling, we isolated the correlation strength between a neuron pair for the –5,000 ms to −3,000 ms and 3,000 ms to 5,000 ms shifted windows. If the maximum correlation strength in the 1–4 ms window was higher than 3,950 of the ±3–5 s shifted values, a neuron pair was deemed to show significant coupling. This threshold corresponded to *α* = 0.05 with a correction for the 4 windows in the 1–4 ms timeframe.

### Behavioural testing in mice subjected to prefrontal cortex PYR–PV^+^ interneuron microcircuit editing

Eight PV-Cre/VGLUT2-flp mice (12–13 weeks old, balanced across sex) were injected with AAV9-CamKII-Cx34.7(M1)-mEmerald (titre: 1.4 × 10^13^ vg per ml) and AAV9-hsyn-DIO-Cx35(M1)-T2A-mCherry (titre: 1.3 × 10^14^ vg per ml) as described above. Seven PV-Cre/VGLUT2-flp control mice were injected with a 1:1 solution of AAV9-CaMKII-eGFP (titre: 2.8 × 10^13^ vg per ml) and AAV9-hSyn-DIO-mCherry (titre: 1.9 × 10^13^ vg per ml) to solely express fluorophores. Two weeks after surgical recovery, mice were subjected to behavioural testing.

The social-preference assay was conducted as previously reported^92^. In brief, mice were habituated to the experimental room (125 lux) for at least 1 h before behavioural testing. Mice were allowed to explore a rectangular arena (61 × 42.5 × 22 cm, L × W × H) for 10 min. Clear plexiglass walls divided the area into two equal chambers with an opening at the centre to allow free exploration. Each chamber contained a circular holding cage (8.3 cm diameter and 12 cm tall) containing either a novel object or a C3H target mouse matched for sex and age. Video data were tracked using Ethovision XT17 (Noldus), whereby the interaction time was identified on the basis of proximity (around 5 cm) to each chamber. Social-preference ratios were calculated as follows: (social interaction time – object interaction time)/(social interaction time + object interaction time).

For open-field testing, mice were placed in a square arena (45.72 × 45.72 × 40.64 cm, L × W × H) for 60 min. The arena was lit at 50 lux, and the location of the mice was tracked using Ethovision XT17 (Noldus). Several days later, mice were placed back into the area, and their location was tracked for an additional 60 min. Data recorded during the first 5 min of the first exposure were used to quantify the novel exploratory drive of mice. Data quantified during the second 60-min exposure were used to quantify gross locomotion function.

### Quantifying MD single-unit responses to direct IL activation

Two mice were injected with AAV9-CamKII-ChR2-EYFP (titre: ≥1 × 10^13^ vg per ml) to target IL bilaterally (1.7 mm AP, ±0.72 mm ML, measured from bregma; –2.03 mm DV from the dura at an angle of 10°). A total of 0.5 µl viral solution was delivered at a rate of 100 nl min^–1^ over 5 min using a Hamilton syringe. Mice were then implanted bilaterally with two optic fibres (MFC_100/125-0.22_8.0mm_MF2.5_FLT, Doric Lenses) directly above the IL (1.7 mm AP, ±0.3 mm ML, measured from bregma, –1.75 mm DV, measured from the dura) and with a 1,024-channel silicon probe (NeuroNexus, SINAPS_4S_1024) to target the MD (–1.58 mm AP, centred at 0.13 mm ML, measured from bregma; –4 mm DV from the dura). The two optic fibres were constructed onto a single holder such that they were implanted at the same depth.

Following a 2-week recovery period, mice were connected to a mezzanine board and headstage and optic fibres without anaesthesia and placed in a new cage. Two 473 nm lasers (CrystaLaser LC, DL473-025-O, CL-2005 Laser Power Supply and Laser Glow, R47-F-473-nm-DPSS-Laser-System/250 mW), calibrated using an optical power meter (ThorLabs PM100D), were used to deliver output at 1 mW. Laser stimulation was triggered using analog output from a Cerebus System (Blackrock Microsystems) to deliver 120 light pulses (10 ms pulse width), each separated by pseudorandomized inter-stimulus intervals ranging from 8 to 24 s. One mouse was stimulated bilaterally and the other mouse was stimulated unilaterally. Neural data were sampled at 20 kHz using a SmartBox Pro acquisition system (NeuroNexus), along with an analog input signal corresponding to the laser trigger. To extract single-unit activity, neural activity was converted to NWB format, high-pass filtered at 300 Hz, and the medial 512 channels (2 medial shanks) were automatically sorted using Kilosort4 (ref. ^94^). We also included one lateral shank that was verified histologically to traverse the MD in one of the mice. Single units were identified on the basis of ISI violations of <0.5, a presence ratio of >0.9 and an amplitude cutoff of <0.1 (ref. ^95^ and https://allensdk.readthedocs.io/en/latest/_static/examples/nb/ecephys_quality_metrics.html) (421 out of 569 total classified cells). Only single units that mapped to the MD-targeted channels (145 out 421 single units) were used for further analyses.

To determine the response of each MD single unit to IL stimulation, we used our previously described approach^49^. In brief, neuronal activity relative to the light stimulation was averaged in 20 ms bins, shifted by 1 ms, and averaged across 120 trials to construct the unit peri-event time histogram. Distributions of the histogram from the [–5,000 ms, –2,000 ms] interval were treated as baseline activity. We then determined which 20-ms bins, slid in 1-ms steps during an epoch spanning from the [0 ms, 30 ms] interval, met the criteria for modulation by cortical stimulation. A unit was found to be modulated by cortical stimulation if at least 20 bins had firing rates either larger than a threshold of 99% above baseline activity or smaller than a threshold of 94% below baseline activity. This approach was modelled after peri-event analytical approaches used in other published studies^96^. To determine the mean LFP evoked responses, neural data recorded from the same channel as each spike were band-pass filtered at 0.5–250 Hz and downsampled to 1,000 Hz. Activity was then averaged across 120 light pulses. Correlations between peri-stimulus firing rate time–histograms and mean LFP evoked responses were calculated for the [0 ms, 30 ms] interval using a linear regression at *α* = 0.05.

### Neural data acquisition and analysis in IL→MD circuit-edited mice

Experiments were conducted during two sessions: 3 weeks and 5 days after the first viral surgery, and again 5 weeks after the first viral surgery. The 473 nm laser was calibrated to deliver output at 3 mW, 1 mW, 0.75 mW, 0.5 mW and 0.25 mW. Laser stimulation was triggered using analog output from a Cerebus System (Blackrock Microsystems). Before recordings, mice were connected to a headstage and optic fibre without anaesthesia and placed in a new cage.

LFP activity was acquired using a Cerebus recording system as described above, concurrently with an analog signal corresponding to the laser TTL pulse. After a 10-min baseline recording period, mice received repeated 10 ms pulses of light in the IL, delivered with pseudorandomized inter-stimulus intervals ranging from 8 to 24 s. During the first session, mice were stimulated with light intensities of 0.25, 0.5, 0.75 and 1 mW (30 trials each) in a pseudorandomized order. The stimulation protocol was fully automated using a Matlab script available from GitHub (https://github.com/carlson-lab/OptoLinCx).

We then determined the proportion of mice that showed clear evoked responses at each light threshold. As our objective was to determine the impact of LinCx expression on IL→MD circuit physiology, our experimental approach required an IL-stimulation intensity that was strong enough to evoke a potential in MD but did not saturate the elicited MD response. When we analysed data from the first session (as described below), we observed that most animals did not show an evoked potential in the MD greater than 50 µV using 0.25, 0.5 or 0.75 mW stimulation. Thus, we used the 1 mW IL stimulation (for which 13 out of 21 mice showed mean evoked responses to >50 µV; Supplementary Fig. 4), to directly assess the impact of LinCx expression of IL→MD circuit function. We then added 30 trials of 3 mW IL stimulation to the second session. Here we reasoned that mice that did not show clear evoked responses in the MD in responses to 3 mW stimulation (>75 µW) would be below the detection threshold for determining the impact of LinCx expression at 1 mW stimulation. Note that these experimental design criteria were implemented in an initial cohort of eight mice before histological confirmation of viral expression and electrode placement. Identical experimental parameters were then used for the remainder of the mice in the study.

Custom Python scripts were used to analyse raw recordings (https://github.com/carlson-lab/OptoLinCx). First, the code loaded raw NSX files and filtered the LFPs with a forward and backward second-order IIR notch filter at 60 Hz, with a quality factor of 30 and a Butterworth analog high-pass filter of order 5 with a cutoff of 15 Hz using Scipy implementation. After filtering, the average voltage in the –200 ms to 1 ms pre-stimulus window was subtracted out, normalizing each evoked response to 0 mV. Trials were excluded if a given trace had more than 50% of the time points identified as outliers from a 1.5 interquartile range between the 25th and 75th quartile. The remaining trials were averaged in each channel and for each stimulus intensity. Channels that showed a pre-stimulus dominant frequency with peaks larger than 25 μV were removed from subsequent analyses. Only animals with at least 15 1 mW trials acquired during both sessions were used. Three mice that did not show a robust response in the MD to the 3 mW stimulus were removed from subsequent analyses.

The mean of the peak amplitude in the 0–10 ms window averaged across all IL microwires, and in the 10–25 ms window averaged across all MD microwires, was used for comparisons across groups. We reasoned that if an increase in the amplitude of MD-evoked potentials was solely driven by an increase in IL-evoked activity, we would observe a direct correlation between the two variables across animals. We did not find such a relationship between the amplitude change of MD-evoked and IL-evoked potentials across the mice that expressed Cx35(M1) (*R* = –0.02; *P* = 0.70, Pearson correlation). Furthermore, we observed a significant stimulation intensity × brain region effect when we compared the magnitude of neural responses across the first session (*F*_3,60_ = 34.32, *P* < 5 × 10^−13^, within-subjects two-way ANOVA; Supplementary Fig. 4). Together, these data established that IL and MD responses to light stimulation were not linearly correlated. Thus, our analysis was performed independently for each brain region.

We note that the first neurophysiological testing session was performed 5 days after electrode implantation, which is far shorter than our typical post-operative recovery period of 14 days. Although this timeline was necessary to obtain neural recording before Cx35(M1) expression (and therefore LinCx formation), this approach of recording neural activity during a period of high post-operative caused concern given that our aim was to quantify the function of a circuit that we knew was affected by stress exposure. We ultimately concluded that the probative value of measuring the function of the IL→MD circuit in a supraphysiological context (direct optogenetic stimulation of IL), exceeded such concerns. Nevertheless, we solely assessed the impact of LinCx expression on the normal physiological function of the IL→MD after a full post-operative recovery period (14 days). Specifically, the modulation index between IL 2–7 Hz and MD 30–70 Hz oscillations was calculated for the second session in which we anticipated strong Cx34.7(M1) trafficking to the MD and strong Cx35(M1) expression, as well as full recovery from surgery. Neural activity recorded from a 10-min baseline period (acquired immediately before light stimulation) was used for analysis. Four to eight LFPs were recorded from each brain region (IL and MD). We then calculated the modulation index for pairs of LFPs recorded from the 2 regions (6–8 per region), which produced up to 64 measured couplings per mouse. These values were then averaged together to produce a single score per animal.

### Behavioural testing of IL→MD circuit manipulation

For this study, behavioural testing was conducted under low illumination conditions (1–2 lux). Two weeks after the second viral surgery, mice were initially placed in a 44.45 × 44.45 × 29.85cm  (L × W × H) chamber for 5 min of open-field testing. Mice were then suspended 1 cm from the tip of their tail for 6 min for the tail-suspension assay. Open-field and tail-suspension behavioural data were acquired during a single testing session, and the entire behavioural testing session was repeated the next day. Testing sessions were video recorded, and open-field and tail-suspension behaviour was analysed using Ethovision XT 12 (Noldus) to quantify immobility in the tail-suspension assay and forward locomotion in the open field. Behavioural experiments and subsequent video analyses were performed blinded to the group allocation of the mice.

### Histology for mouse studies

Mice were perfused transcardially with ice-cold PBS followed by 4% PFA in PBS (EM Sciences). The brains were collected and coronally sliced in 1× PBS at 35 µm using a vibratome (Vibratome Series 3000 Plus, The Vibratome) and mounted onto positively charged slides using a mild acetate buffer (82.4 mM sodium acetate and 17.6 mM acetic acid) or 1× PBS solution. Brain slices were covered with DAPI-Mowiol mounting solution (glycerol, puriss. p.a., Mowiol 4-88 (Sigma-Aldrich), and 0.2 M Tris-Cl pH 8.5, DAPI (Sigma-Aldrich)) and coverslipped before imaging. Images were obtained using a Nikon Eclipse fluorescence microscope at ×4 magnification with the illumination source power and exposure kept consistent between samples.

For the PrL PYR–PV^+^ interneuron microcircuit-editing experiment, we only used mice with confirmed bilateral connexin expression for neurophysiological analysis (*n* = 26 out of the 29 brains analysed). We also performed immunohistochemistry to assess the specificity of our viral targeting approach for this study. In brief, we injected three C57BL/6J mice in the PrL with AAV-CamKII-Cx34.7(M1)-mEmerald. Following a 4-week expression period, brains were removed, sliced and stained. We performed immunohistochemistry using anti-PV antibody (1:1,000 rabbit anti-PV (Abcam ab11427)) and AlexaFluor 568 dye. Neurons were identified by DAPI expression and a diameter of >13 µm. Green fluorescence (indicating viral expression) and red fluorescence (indicating PV expression) was then quantified across all neurons in the PrL. Using this approach, we found that only 4.3% of all cells that expressed Cx34.7(M1) also expressed PV, and 15.5% of PV^+^ interneurons expressed Cx35(M1). Thus, most of the cells that expressed Cx34.7(M1) were excitatory.

For the IL→MD behavioural study, we used mice that showed bilateral connexin expression in one region and at least unilateral expression in the other region (*n* = 26 out of the 35 brains analysed). We chose this strategy because our previous work indicated that unilateral optogenetic stimulation of the IL→MD circuit was sufficient to alter stress-related behaviour in the tail-suspension assay^50^. For the IL→MD behavioural study, histology was performed as described above to confirm electrode placement, EYFP–mEmerald trafficking to the MD and mApple expression in the MD. In a subset of animals, verification of MD expression required additional staining against mApple. Here we used a rabbit primary antibody against RFP (Rockland, 600-401-379) and a secondary antibody with Alexa Fluor 568 (Abcam, ab175471). ChR2 expression was confirmed via electrophysiology (that is, neural responses to blue light). Only mice with accurate targeting of both viral sites and electrodes were used for analysis (*n* = 6 control mice and 9 LinCx-edited mice).

### Statistical philosophy

We used nonparametric and parametric tests throughout. We used parametric tests for our flow cytometry, *Xenopus* oocyte preparation electrophysiology and behaviour analyses given that such an approach was the standard in the field. For these experiments, data were assumed to be drawn from a normal distribution, although formal testing was not performed. We used nonparametric tests for our analysis of transjunctional currents in the HEK293FT cell preparation and for our in vivo electrophysiological analyses based on oscillatory coupling. For the analyses in which we had clear priors or a directional hypothesis, we used a one-tailed test. In most cases, our prior was that the expression of docking-compatible connexin pairs produced higher measures of coupling between cells (that is, docking-compatible pairs will show higher FETCH scores compared with a known-negative distribution of non-docking cell pairs; electrical synapse formation will increase transjunctional current between HEK293FT cell pairs lacking Cx43 and Cx45; LinCx expression will increase PYR–PV^+^ interneuron cross-correlated firing; and LinCx expression will potentiate the IL→MD circuit). The one-tailed approach was particularly useful for the in vivo mouse physiological studies to limit the number of animals needed. All studies for which there was no clear prior or directional hypothesis used two-tailed tests (for example, comparison of LinCx versus Cx36–Cx36 FETCH scores; impact of LinCx expression on LFP power and unit firing rates; impact of mPFC PYR–PV^+^ interneuron microcircuit editing on distance travelled), unless otherwise specified. In some instances, additional control analyses requested via peer review were performed on collected data. Such testing is described as secondary analyses in the paper. The initial testing of our priors was considered independent from such secondary analysis and therefore was not subject to multiplicity correction owing to these secondary analyses. In all instances, we provide complete statistical reporting, including *P* values, *t* stats, *F* stats, degrees of freedom and *U* statistics, where appropriate. When multiple hypothesis testing was used, we report uncorrected *P* values, the approach to *α* threshold correction, and the number of comparisons for which the correction was applied. Finally, we report effect sizes for the key in vivo studies that directly tested whether LinCx expression increased our a priori measures of physiological coupling (when moving across levels of analysis from cells to behaviour, Supplementary Table 3). For each instance when the effect size is reported using Cohen’s *D*, we verified that the data did not deviate from a normal distribution using a Kolmogorov–Smirnov test. Such detailed reporting enables an independent post hoc assessment of the robustness of our observations in the absence of specific directional-hypothesis or multiple-hypothesis testing.

### Data visualization

For bar graphs, data are plotted as the mean ± s.e.m. Box and whisker plots were created using the Matlab boxplot function. The central mark is the median, the edges of the box are the 25th and 75th percentiles, the whiskers extend to the most extreme data points the algorithm does not consider to be outliers, and the outliers are plotted individually as a plus symbol.

### Material availability

All non-publicly available materials used in this study will be made upon reasonable request. Requests for such materials should be addressed to the corresponding author.

### Reporting summary

Further information on research design is available in the Nature Portfolio Reporting Summary linked to this article.

## Online content

Any methods, additional references, Nature Portfolio reporting summaries, source data, extended data, supplementary information, acknowledgements, peer review information; details of author contributions and competing interests; and statements of data and code availability are available at 10.1038/s41586-026-10501-y.

## Supplementary information

Supplementary Information Supplementary Tables 1–3, Supplementary Figs. 1–5 and supplementary references.

Reporting Summary

## Data Availability

All data generated in support of the findings of this study are available from the corresponding author for academic purposes upon reasonable request. Such data will be made available under a material transfer agreement. Connexin gene information was procured from the National Center for Biotechnology Information (https://www.ncbi.nlm.nih.gov) and the Ensembl genome browser (http://ensembl.org), and are readily accessible.

## Code availability

The following codes for the methods implemented in this study are available from GitHub: (1) https://github.com/carlson-lab/FETCH; (2) https://github.com/carlson-lab/VMD-and-NAMD-Connexin-Protein-Simulation-Protocol; (3) https://github.com/carlson-lab/OptoLinCx. All are available stored in an online repository (10.7924/r4r486).
