# Supplementary material for: Long-term editing of brain circuits using an engineered electrical synapse
Source: Nature. 2026 May 13;655(8123):703–15. doi: 10.1038/s41586-026-10501-y (PMC13372691; doi:10.1038/s41586-026-10501-y)
Supplement: Supplementary file 1 — Supplementary Tables 1–3, Supplementary Figs. 1–5 and supplementary references. [file 41586_2026_10501_MOESM1_ESM.pdf]

nature portfolio

https://doi.org/10.1038/s41586-026-10501-y

# Supplementary information

# Long-term editing of brain circuits using an engineered electrical synapse

In the format provided by the authors and unedited

**Supplemental Table S1: FETCH scores for homotypic and heterotypic screening of Cx34.7 and Cx35 mutants**

| Cx34.7 | | |
|---|---|---|
| Mutant | mean | st-err |
| 'D216E' | 17.60 | 4.29 |
| 'D216H' | 17.51 | 3.86 |
| 'D216K' | 18.14 | 4.08 |
| 'D216N' | 19.08 | 2.32 |
| 'D216Q' | 17.20 | 2.96 |
| 'D216R' | 14.91 | 4.04 |
| 'E214A' | 14.37 | 3.73 |
| 'E214H' | 13.95 | 2.98 |
| 'E214K' | 11.44 | 1.71 |
| 'E214N' | 17.16 | 3.65 |
| 'E214Q' | 17.98 | 2.97 |
| 'E214R' | 6.23 | 0.68 |
| 'E214T' | 19.36 | 3.87 |
| 'E214V' | 18.32 | 3.26 |
| 'E223D' | 17.37 | 4.63 |
| 'E223H' | 14.81 | 2.45 |
| 'E223K' | 14.87 | 2.67 |
| 'E223N' | 17.77 | 3.86 |
| 'E223P' | 19.97 | 2.95 |
| 'E223Q' | 19.82 | 4.06 |
| 'E223R' | 14.47 | 3.42 |
| 'E223S' | 18.75 | 3.52 |
| 'E223T' | 16.72 | 4.05 |
| 'E223Y' | 13.21 | 3.97 |
| 'E225D' | 12.76 | 3.57 |
| 'E225H' | 16.58 | 2.84 |
| 'E225K' | 0.95 | 0.25 |
| 'E225N' | 16.22 | 1.49 |
| 'E225R' | 1.15 | 0.49 |
| 'E43K' | 27.39 | 3.25 |
| 'I54H' | 21.29 | 1.68 |
| 'I54K' | 21.46 | 3.90 |
| 'I54R' | 21.29 | 3.49 |
| 'I54V' | 25.43 | 2.84 |
| 'K222H' | 20.73 | 1.75 |
| 'K222N' | 19.51 | 0.56 |
| 'K222Q' | 1.17 | 0.26 |

| Cx35 | | |
|---|---|---|
| Mutant | mean | st-err |
| 'D215E' | 23.32 | 1.06 |
| 'D215H' | 19.71 | 0.64 |
| 'D215K' | 19.17 | 1.59 |
| 'D215N' | 24.34 | 1.99 |
| 'D215Q' | 22.81 | 1.55 |
| 'D215R' | 22.93 | 1.56 |
| 'D222E' | 28.96 | 3.88 |
| 'D222H' | 29.65 | 1.54 |
| 'D222K' | 29.45 | 2.63 |
| 'D222N' | 27.98 | 4.56 |
| 'D222P' | 28.61 | 4.26 |
| 'D222Q' | 27.37 | 1.36 |
| 'D222R' | 27.17 | 2.39 |
| 'D222S' | 27.89 | 1.83 |
| 'D222T' | 27.16 | 1.20 |
| 'D222Y' | 24.93 | 1.62 |
| 'E213H' | 25.36 | 1.24 |
| 'E213K' | 9.80 | 1.06 |
| 'E213R' | 21.13 | 1.91 |
| 'E224D' | 19.75 | 0.53 |
| 'E224H' | 1.47 | 0.15 |
| 'E224K' | 2.80 | 0.54 |
| 'E224Q' | 27.08 | 3.70 |
| 'E224R' | 1.54 | 0.25 |
| 'E43K' | 30.57 | 1.21 |
| 'K221H' | 35.12 | 0.63 |
| 'K221N' | 31.48 | 2.38 |
| 'K221Q' | 14.00 | 2.23 |
| 'K221R' | 30.19 | 1.20 |
| 'K69H' | 32.12 | 1.36 |
| 'K69N' | 28.69 | 1.91 |
| 'K69Q' | 26.14 | 1.52 |
| 'K69R' | 22.06 | 1.53 |
| 'L237S' | 15.51 | 0.95 |
| 'L237T' | 11.40 | 0.77 |
| 'L237Y' | 0.40 | 0.12 |
| 'M52A' | 27.11 | 0.96 |

| Cx34.7 - Cx35 | | |
|---|---|---|
| Mutant | mean | st-err |
| E225K - E224H' | 0.66 | 0.12 |
| 'E225K - E224K' | 0.84 | 0.11 |
| 'E225K - E224R' | 0.90 | 0.12 |
| 'E225K - L237Y' | 0.44 | 0.03 |
| 'E225K - N56E' | 1.06 | 0.13 |
| 'E225K - Y78S' | 0.56 | 0.09 |
| 'E225K - Y78T' | 0.35 | 0.04 |
| 'E225K - Y78V' | 0.41 | 0.05 |
| 'E225R - E224H' | 0.83 | 0.08 |
| 'E225R - E224K' | 0.69 | 0.13 |
| 'E225R - E224R' | 0.80 | 0.13 |
| 'E225R - L237Y' | 4.40 | 0.74 |
| 'E225R - N56E' | 4.06 | 0.21 |
| 'E225R - Y78S' | 0.54 | 0.12 |
| 'E225R - Y78T' | 0.50 | 0.10 |
| 'E225R - Y78V' | 0.37 | 0.06 |
| 'K222Q - E224H' | 51.59 | 0.77 |
| 'K222Q - E224K' | 45.31 | 0.72 |
| 'K222Q - E224R' | 47.81 | 1.15 |
| 'K222Q - L237Y' | 7.33 | 0.85 |
| 'K222Q - N56E' | 0.83 | 0.13 |
| 'K222Q - Y78S' | 0.60 | 0.04 |
| 'K222Q - Y78T' | 0.52 | 0.08 |
| 'K222Q - Y78V' | 0.54 | 0.05 |
| 'L238Y - E224H' | 10.04 | 0.87 |
| 'L238Y - E224K' | 0.61 | 0.11 |
| 'L238Y - E224R' | 1.05 | 0.17 |
| 'L238Y - L237Y' | 3.11 | 0.43 |
| 'L238Y - N56E' | 1.28 | 0.21 |
| 'L238Y - Y78S' | 0.65 | 0.12 |
| 'L238Y - Y78T' | 0.41 | 0.13 |
| 'L238Y - Y78V' | 0.71 | 0.14 |
| 'Y78S - E224H' | 0.77 | 0.16 |
| 'Y78S - E224K' | 0.92 | 0.18 |
| 'Y78S - E224R' | 0.87 | 0.18 |
| 'Y78S - L237Y' | 0.51 | 0.08 |
| 'Y78S - N56E' | 0.83 | 0.22 |

| | | |
|---|---|---|
| 'K222R' | 17.99 | 3.30 |
| 'K69H' | 21.22 | 2.41 |
| 'K69N' | 17.91 | 4.20 |
| 'K69Q' | 17.91 | 3.03 |
| 'K69R' | 19.61 | 4.62 |
| 'L238S' | 12.04 | 2.62 |
| 'L238T' | 18.16 | 2.67 |
| 'L238Y' | 4.82 | 1.94 |
| 'M52A' | 17.98 | 3.48 |
| 'M52D' | 17.05 | 3.75 |
| 'M52E' | 17.21 | 2.73 |
| 'M52G' | 19.98 | 3.14 |
| 'M52K' | 2.81 | 0.36 |
| 'M52T' | 14.07 | 3.08 |
| 'N56D' | 10.17 | 3.27 |
| 'N56E' | 19.76 | 3.19 |
| 'N56Q' | 16.03 | 4.35 |
| 'N63E' | 18.26 | 2.69 |
| 'N63H' | 13.68 | 1.39 |
| 'N63K' | 15.84 | 2.52 |
| 'N63R' | 18.16 | 1.29 |
| 'Q50K' | 19.84 | 3.30 |
| 'R217A' | 24.19 | 4.26 |
| 'R217H' | 19.40 | 3.01 |
| 'R217K' | 19.39 | 3.69 |
| 'R217V' | 22.45 | 4.41 |
| 'V80A' | 13.81 | 4.22 |
| 'V80S' | 10.30 | 2.38 |
| 'WT' | 20.91 | 3.60 |
| 'Y78F' | 20.77 | 4.77 |
| 'Y78L' | 17.83 | 1.98 |
| 'Y78S' | 0.45 | 0.18 |
| 'Y78T' | 0.54 | 0.16 |
| 'Y78V' | 1.36 | 0.50 |

| | | |
|---|---|---|
| 'M52D' | 29.17 | 1.76 |
| 'M52E' | 20.79 | 1.62 |
| 'M52G' | 25.50 | 2.68 |
| 'M52R' | 21.61 | 1.33 |
| 'M52S' | 25.29 | 3.11 |
| 'M52T' | 28.47 | 1.12 |
| 'N56D' | 15.23 | 1.66 |
| 'N56E' | 2.35 | 0.30 |
| 'N56Q' | 25.26 | 2.12 |
| 'N63D' | 23.74 | 1.51 |
| 'N63E' | 29.71 | 2.04 |
| 'N63H' | 26.38 | 2.56 |
| 'N63K' | 14.71 | 1.13 |
| 'N63Q' | 30.98 | 1.58 |
| 'N63R' | 24.12 | 4.12 |
| 'Q50K' | 24.15 | 3.20 |
| 'R216A' | 30.29 | 1.40 |
| 'R216H' | 25.45 | 1.47 |
| 'R216K' | 23.01 | 1.33 |
| 'R216V' | 28.86 | 3.37 |
| 'V54H' | 26.95 | 2.13 |
| 'V54I' | 29.45 | 1.95 |
| 'V54R' | 19.91 | 1.97 |
| 'V80S' | 7.53 | 0.91 |
| 'WT' | 25.05 | 3.22 |
| 'Y78F' | 26.63 | 1.05 |
| 'Y78L' | 8.19 | 0.83 |
| 'Y78S' | 0.66 | 0.12 |
| 'Y78T' | 0.73 | 0.20 |
| 'Y78V' | 0.86 | 0.18 |

| | | |
|---|---|---|
| 'Y78S - Y78S' | 0.59 | 0.21 |
| 'Y78S - Y78T' | 0.48 | 0.10 |
| 'Y78S - Y78V' | 0.25 | 0.07 |
| 'Y78T - E224H' | 0.72 | 0.15 |
| 'Y78T - E224K' | 0.63 | 0.11 |
| 'Y78T - E224R' | 0.90 | 0.13 |
| 'Y78T - L237Y' | 0.49 | 0.10 |
| 'Y78T - N56E' | 0.88 | 0.18 |
| 'Y78T - Y78S' | 0.53 | 0.07 |
| 'Y78T - Y78T' | 0.36 | 0.09 |
| 'Y78T - Y78V' | 0.42 | 0.06 |
| 'Y78V - E224H' | 1.08 | 0.14 |
| 'Y78V - E224K' | 0.53 | 0.13 |
| 'Y78V - E224R' | 1.13 | 0.13 |
| 'Y78V - L237Y' | 0.70 | 0.09 |
| 'Y78V - N56E' | 0.88 | 0.07 |
| 'Y78V - Y78S' | 0.52 | 0.10 |
| 'Y78V - Y78T' | 0.46 | 0.05 |
| 'Y78V - Y78V' | 0.50 | 0.07 |

**Supplemental Table S2: *C. elegans* strain table**

| | Genotype | Source | Line # |
|---|---|---|---|
| N2 | *Wild-type* | CGC | |
| DCR3056 | *olaIs17 [Pmod-1::GCaMP6s (25ng/ul) Pttx-3::mCherry (25ng/ul) Punc-122::dsRed (40ng/ul)] I* | Hawk et al. 2018[1] | |
| DCR6604 | *olaIs23 [Pgcy-8(800)::caPKC-1B (30ng/ul), Pgcy-8(800)::tagRFP (10ng/ul), Punc-122::RFP (30ng/ul)] V; wyIs629 [Pgcy-8(2kb)::GCaMP6s (30ng/ul), Pgcy-8(2kb)::mCherry (5ng/ul), Punc-122:GFP (20ng/ul)] X* | Hawk et al. 2018[1] | |
| DCR5790 | *olaIs17 [Pmod-1::GCaMP6s (25ng/ul) Pttx-3::mCherry (25ng/ul) Punc-122::dsRed (40ng/ul)] I; olaIs72 [Pelt-7::mCherry (25ng/ul) + Pttx-3::CX36::mCherry (25ng/ul)]* | Hawk et al. 2018[1] | |
| DCR5793 | *olaIs17 [Pmod-1::GCaMP6s (25ng/ul) Pttx-3::mCherry (25ng/ul) Punc-122::dsRed (40ng/ul)] I; olaIs70 [Pelt-7::GFP (15ng/ul) + Pgcy-8::CX36::mCherry (25ng/ul)]; olaIs72 [Pelt-7::mCherry (25ng/ul) + Pttx-3::CX36::mCherry (25ng/ul)]* | Hawk et al. 2018[1] | |
| DCR5404 | *olaIs17 [Pmod-1::GCaMP6s (25ng/ul) Pttx-3::mCherry (25ng/ul) Punc-122::dsRed (40ng/ul)] I; olaIs23 [Pgcy-8(800)::caPKC-1B (30ng/ul), Pgcy-8(800)::tagRFP (10ng/ul), Punc-122::RFP (30ng/ul)] V; olaEx3219 [Pgcy-8::CX36::mCherry (25ng/ul); Pttx-3::CX36::mCherry (25ng/ul); Pmyo-3::Red(10ng/ul)]* | Hawk et al. 2018[1] | |
| DCR8225 | *olaIs17 [Pmod-1::GCaMP6s (25ng/ul) Pttx-3::mCherry (25ng/ul) Punc-122::dsRed (40ng/ul)] I; olaIs23 [Pgcy-8(800)::caPKC-1B (30ng/ul), Pgcy-8(800)::tagRFP (10ng/ul), Punc-122::RFP (30ng/ul)] V 13xOC* | This Paper | |
| DCR8678 | *olaEx5223 [Pgcy-8::CX34.7::GFP; Pttx-3::CX34.7::mCherry; Punc-122::GFP (All 25ng/ul)]* | This Paper | Line 1 |
| DCR8717 | *olaEx5255 [Pgcy-8::CX34.7::GFP; Pttx-3::CX34.7::mCherry; Punc-122::GFP (All 25ng/ul)]* | This Paper | Line 1 |
| DCR8716 | *olaEx5254 [Pgcy-8::CX34.7(E214K, E223K)::GFP; Pttx-3::CX34.7(E214K, E223K)::mCherry; Punc-122::GFP (All 25ng/ul)]* | This Paper | Line 1 |
| DCR8673 | *olaEx5218 [Pgcy-8::CX35(K221E)::GFP; Pttx-3::CX35(K221E)::mCherry; Punc-122::GFP (All 25ng/ul)]* | This Paper | Line 1 |
| DCR8669 | *olaEx5214 [Pgcy-8::CX34.7(E214K, E223K)::GFP; Pttx-3::CX35(K221E)::mCherry; Punc-122::GFP (All 25ng/ul)]* | This Paper | Line 1 |
| DCR8684 | *olaEx5230 [Pgcy-8::CX35::GFP; Pttx-3::CX35::mCherry; Punc-122::GFP (All 25ng/ul)]* | This Paper | Line 2 |
| DCR8719 | *olaEx5256 [Pgcy-8::CX35::GFP; Pttx-3::CX35::mCherry; Punc-122::GFP (All 25ng/ul)]* | This Paper | Line 2 |
| DCR8715 | *olaEx5253 [Pgcy-8::CX34.7(E214K, E223K)::GFP; Pttx-3::CX34.7(E214K, E223K)::mCherry; Punc-122::GFP (All 25ng/ul)]* | This Paper | Line 2 |
| DCR8674 | *olaEx5219 [Pgcy-8::CX35(K221E)::GFP; Pttx-3::CX35(K221E)::mCherry; Punc-122::GFP (All 25ng/ul)]* | This Paper | Line 2 |

| | | | |
|---|---|---|---|
| DCR8676 | *olaEx5221 [Pgcy-8::CX34.7(E214K, E223K)::GFP; Pttx-3::CX35(K221E)::mCherry; Punc-122::GFP (All 25ng/ul)]* | This Paper | Line 2 |
| DCR8685 | *olaEx5231 [Pgcy-8::CX34.7::GFP; Pttx-3::CX34.7::mCherry; Punc-122::GFP (All 25ng/ul)]* | This Paper | Line 3 |
| DCR8720 | *olaEx5257 [Pgcy-8::CX35::GFP; Pttx-3::CX35::mCherry; Punc-122::GFP (All 25ng/ul)]* | This Paper | Line 3 |
| DCR8714 | *olaEx5252 [Pgcy-8::CX34.7(E214K, E223K)::GFP; Pttx-3::CX34.7(E214K, E223K)::mCherry; Punc-122::GFP (All 25ng/ul)]* | This Paper | Line 3 |
| DCR8672 | *olaEx5217 [Pgcy-8::CX35(K221E)::GFP; Pttx-3::CX35(K221E)::mCherry; Punc-122::GFP (All 25ng/ul)]* | This Paper | Line 3 |
| DCR8677 | *olaEx5222 [Pgcy-8::CX34.7(E214K, E223K)::GFP; Pttx-3::CX35(K221E)::mCherry; Punc-122::GFP (All 25ng/ul)]* | This Paper | Line 3 |
| DCR8675 | *olaIs17 [Pmod-1::GCaMP6s (25ng/ul) Pttx-3::mCherry (25ng/ul) Punc-122::dsRed (40ng/ul)] I; olaIs23 [Pgcy-8(800)::caPKC-1B (30ng/ul), Pgcy-8(800)::tagRFP (10ng/ul), Punc-122::RFP (30ng/ul)] V; olaEx5220 [Pgcy-8::CX34.7::GFP; Pttx-3::CX34.7::mCherry; Pelt-7::NLS::mCherry (All 25ng/ul)]* | This Paper | Line 1 |
| DCR8776 | *olaIs17 [Pmod-1::GCaMP6s (25ng/ul) Pttx-3::mCherry (25ng/ul) Punc-122::dsRed (40ng/ul)] I; olaIs23 [Pgcy-8(800)::caPKC-1B (30ng/ul), Pgcy-8(800)::tagRFP (10ng/ul), Punc-122::RFP (30ng/ul)] V; olaEx5287 [Pgcy-8::CX35::GFP; Pttx-3::CX35::mCherry; Punc-122::GFP (All 25ng/ul)]* | This Paper | Line 1 |
| DCR8777 | *olaIs17 [Pmod-1::GCaMP6s (25ng/ul) Pttx-3::mCherry (25ng/ul) Punc-122::dsRed (40ng/ul)] I; olaIs23 [Pgcy-8(800)::caPKC-1B (30ng/ul), Pgcy-8(800)::tagRFP (10ng/ul), Punc-122::RFP (30ng/ul)] V; olaEx5288 [Pgcy-8::CX34.7(E214K, E223K)::GFP; Pttx-3::CX34.7(E214K, E223K)::mCherry; Punc-122::GFP (All 25ng/ul)]* | This Paper | Line 1 |
| DCR8671 | *olaIs17 [Pmod-1::GCaMP6s (25ng/ul) Pttx-3::mCherry (25ng/ul) Punc-122::dsRed (40ng/ul)] I; olaIs23 [Pgcy-8(800)::caPKC-1B (30ng/ul), Pgcy-8(800)::tagRFP (10ng/ul), Punc-122::RFP (30ng/ul)] V; olaEx5216 [Pgcy-8::CX35(K221E)::GFP; Pttx-3::CX35(K221E)::mCherry; Pelt-7::NLS::mCherry (All 25ng/ul)]* | This Paper | Line 1 |
| DCR8670 | *olaIs17 [Pmod-1::GCaMP6s (25ng/ul) Pttx-3::mCherry (25ng/ul) Punc-122::dsRed (40ng/ul)] I; olaIs23 [Pgcy-8(800)::caPKC-1B (30ng/ul), Pgcy-8(800)::tagRFP (10ng/ul), Punc-122::RFP (30ng/ul)] V; olaEx5215 [Pgcy-8::CX34.7(E214K, E223K)::GFP; Pttx-3::CX35(K221E)::mCherry; Pelt-7::NLS::mCherry (All 25ng/ul)]* | This Paper | Line 1 |
| DCR9178 | *olaEx5452 [Pgcy-8::CX34.7(E214K, E223K)::GFP; Pttx-3::CX43::mCherry; Pelt-7::NLS::mCherry (All 25ng/ul)]* | This Paper | Line 1 |
| DCR9179 | *olaEx5453 [Pgcy-8::CX34.7(E214K, E223K)::GFP; Pttx-3::CX43::mCherry; Pelt-7::NLS::mCherry (All 25ng/ul)]* | This Paper | Line 2 |

| | | | |
|---|---|---|---|
| DCR9180 | *olaEx5454 [Pgcy-8::CX34.7(E214K, E223K)::GFP; Pttx-3::CX43::mCherry; Pelt-7::NLS::mCherry (All 25ng/ul)]* | This Paper | Line 3 |
| DCR9181 | *olaIs17 [Pmod-1::GCaMP6s (25ng/ul) Pttx-3::mCherry (25ng/ul) Punc-122::dsRed (40ng/ul)] I; olaIs23 [Pgcy-8(800)::caPKC-1B (30ng/ul), Pgcy-8(800)::tagRFP (10ng/ul), Punc-122::RFP (30ng/ul)] V; olaEx5455 [Pgcy-8::CX34.7(E214K, E223K)::GFP; Pttx-3::CX43::mCherry; Pelt-7::NLS::mCherry (All 25ng/ul)]* | This Paper | Line 1 |
| DCR9182 | *olaEx5456 [Pgcy-8::CX43::GFP; Pttx-3::CX35(K221E)::mCherry; Pelt-7::NLS::mCherry (All 25ng/ul)]* | This Paper | Line 1 |
| DCR9183 | *olaIs17 [Pmod-1::GCaMP6s (25ng/ul) Pttx-3::mCherry (25ng/ul) Punc-122::dsRed (40ng/ul)] I; olaIs23 [Pgcy-8(800)::caPKC-1B (30ng/ul), Pgcy-8(800)::tagRFP (10ng/ul), Punc-122::RFP (30ng/ul)] V; olaEx5457 [Pgcy-8::CX43::GFP; Pttx-3::CX35(K221E)::mCherry; Pelt-7::NLS::mCherry (All 25ng/ul)]* | This Paper | Line 1 |
| DCR9184 | *olaIs17 [Pmod-1::GCaMP6s (25ng/ul) Pttx-3::mCherry (25ng/ul) Punc-122::dsRed (40ng/ul)] I; olaIs72 [Pelt-7::mCherry (25ng/ul) + Pttx-3::CX36::mCherry (25ng/ul)]; olaEx5458 [Pgcy-8::CX34.7(E214K, E223K)::GFP; Pelt-7::NLS::GFP (All 25ng/ul)]* | This Paper | Line 1 |
| DCR9185 | *olaIs17 [Pmod-1::GCaMP6s (25ng/ul) Pttx-3::mCherry (25ng/ul) Punc-122::dsRed (40ng/ul)] I; olaIs72 [Pelt-7::mCherry (25ng/ul) + Pttx-3::CX36::mCherry (25ng/ul)]; olaEx5459 [Pgcy-8::CX34.7(E214K, E223K)::GFP; Pelt-7::NLS::GFP (All 25ng/ul)]* | This Paper | Line 2 |
| DCR9186 | *olaIs17 [Pmod-1::GCaMP6s (25ng/ul) Pttx-3::mCherry (25ng/ul) Punc-122::dsRed (40ng/ul)] I; olaIs72 [Pelt-7::mCherry (25ng/ul) + Pttx-3::CX36::mCherry (25ng/ul)]; olaEx5460 [Pgcy-8::CX34.7(E214K, E223K)::GFP; Pelt-7::NLS::GFP (All 25ng/ul)]* | This Paper | Line 3 |
| DCR7790 | *olaIs17 [Pmod-1::GCaMP6s (25ng/ul) Pttx-3::mCherry (25ng/ul) Punc-122::dsRed (40ng/ul)] I; olaIs70 [Pelt-7::GFP (15ng/ul) + Pgcy-8::CX36::mCherry (25ng/ul)]* | This Paper | |
| DCR9187 | *olaIs17 [Pmod-1::GCaMP6s (25ng/ul) Pttx-3::mCherry (25ng/ul) Punc-122::dsRed (40ng/ul)] I; olaIs70 [Pelt-7::GFP (15ng/ul) + Pgcy-8::CX36::mCherry (25ng/ul)]; olaEx5461 [Pttx-3::CX35(K221E)::mCherry; Pelt-7::NLS::mCherry (All 25ng/ul)]* | This Paper | Line 1 |
| DCR9188 | *olaIs17 [Pmod-1::GCaMP6s (25ng/ul) Pttx-3::mCherry (25ng/ul) Punc-122::dsRed (40ng/ul)] I; olaIs70 [Pelt-7::GFP (15ng/ul) + Pgcy-8::CX36::mCherry (25ng/ul)]; olaEx5462 [Pttx-3::CX35(K221E)::mCherry; Pelt-7::NLS::mCherry (All 25ng/ul)]* | This Paper | Line 2 |

| | | | |
|---|---|---|---|
| DCR9189 | *olaIs17 [Pmod-1::GCaMP6s (25ng/ul) Pttx-3::mCherry (25ng/ul) Punc-122::dsRed (40ng/ul)] I; olaIs70 [Pelt-7::GFP (15ng/ul) + Pgcy-8::CX36::mCherry (25ng/ul)]; olaEx5463 [Pttx-3::CX35(K221E)::mCherry; Pelt-7::NLS::mCherry (All 25ng/ul)]* | This Paper | Line 3 |

**Supplemental Table S3: Summary of effect sizes resulting from LinCx circuit editing.**

| Circuit | Experiment | tails | n (LinCx and Control) | Cohen's D or phi | P-value |
|---|---|---|---|---|---|
| PYR-PV+ | PV+-PYR coupling | N/A | 2815 and 2801 neuron pairs | Phi: 0.08 | <0.0001 |
| | Theta-PV+ Coupling | 1 | 101 and 91 neurons | CD: 0.33 | 0.01 |
| | Theta-HFO Coupling | 1 | 11 and 14 mice | CD: 0.99 | 0.013 |
| | **Social Behavior** | **1** | **8 and 7 mice** | **CD: 1.1** | **0.012** |
| | | | | | |
| IL→MD | IL-MD (Optostimulation) | 1 | 9 and 6 mice | CD: 0.91 | 0.043 |
| | IL-MD coupling | 1 | 9 and 6 mice | CD: 0.92 | 0.033 |
| | **TST behavior** | **NA** | **10 and 16 mice** | **CD: 1** | **0.01** |

## Cx36 Extracellular Loop 2

```
Homo sapiens           227- GLYECNRYPCIKEVECYVSRPTEKTVFLVF -256
Mus musculus           227- GLYECNRYPCIKEVECYVSRPTEKTVFLVF -256
Macaca mulatta         227- GLYECNRYPCIKEVECYVSRPTEKTVFLVF -256
Callithrix jacchus     289- GLYECNRYPCIKEVECYVSRPTEKTVFLVF -318
Taeniopygia guttata    196- AIFECDRYPCVKEVECYVSRPTEKSVFLVF -225
Danio rerio (Cx34.7)   209- GIFECDRYPCLKEVECYVSRPTEKTVFLVF -238
Danio rerio (Cx35)     210- AVYECDRYPCIKDVECYVSRPTEKTVFLVF -239
```

## Cx43 Extracellular Loop 2

```
Homo sapiens           171- LIQWYIYGFSLSAVYTCKRDPCPHQVDCFLSRPTEK -206
Mus musculus           171- LIQWYIYGFSLSAVYTCKRDPCPHQVDCFLSRPTEK -206
Macaca mulatta         171- LIQWYIYGFSLSAVYTCKRDPCPHQVDCFLSRPTEK -206
Callithrix jacchus     171- LIQWYIYGFSLSAVYTCKRDPCPHQVDCFLSRPTEK -206
Taeniopygia guttata    171- LIQWYIYGFSLNAIYTCERDPCPHRVDCFLSRPTEK -206
Danio rerio            171- VIQWYLYGFSLSAVYTCERTPCPHRVDCFLSRPTEK -206
```

## Cx45 Extracellular Loop 2

```
Homo sapiens           200- GFQVHPFYVCSRLPCPHKIDCFI -222
Mus musculus           200- GFQVHPFYVCSRLPCPHKIDCFI -222
Macaca mulatta         200- GFQVHPFYVCSRLPCPHKIDCFI -222
Callithrix jacchus     200- GFQVHPFYVCSRLPCPHKIDCFI -222
Taeniopygia guttata    198- RFEVSPSYVCSRSPCPHTVDCFV -220
Danio rerio            196- GFEVAPSYVCTRSPCPHTVDCFV -218
```

**Supplemental Figure S1: Sequence alignment of several connexin proteins predicted for extracellular loop 2 (EL2).** Predicted EL2 regions of Cx36 (GJD2), Cx43 (GJA1), and Cx45 (GJC1) for humans and several species broadly utilized in neuroscience research, related to Fig. 1D. Identical residues across species are shown in black, residues that are variable are highlighted with red text. Residues of Cx36 that align to the interaction motif of Cx34.7 and Cx35 are indicated by dark blue underline. Note zebrafish (*Danio rerio*) do not have a Cx36 gene, thus the closest homolog, Cx34.7, was used for comparison.

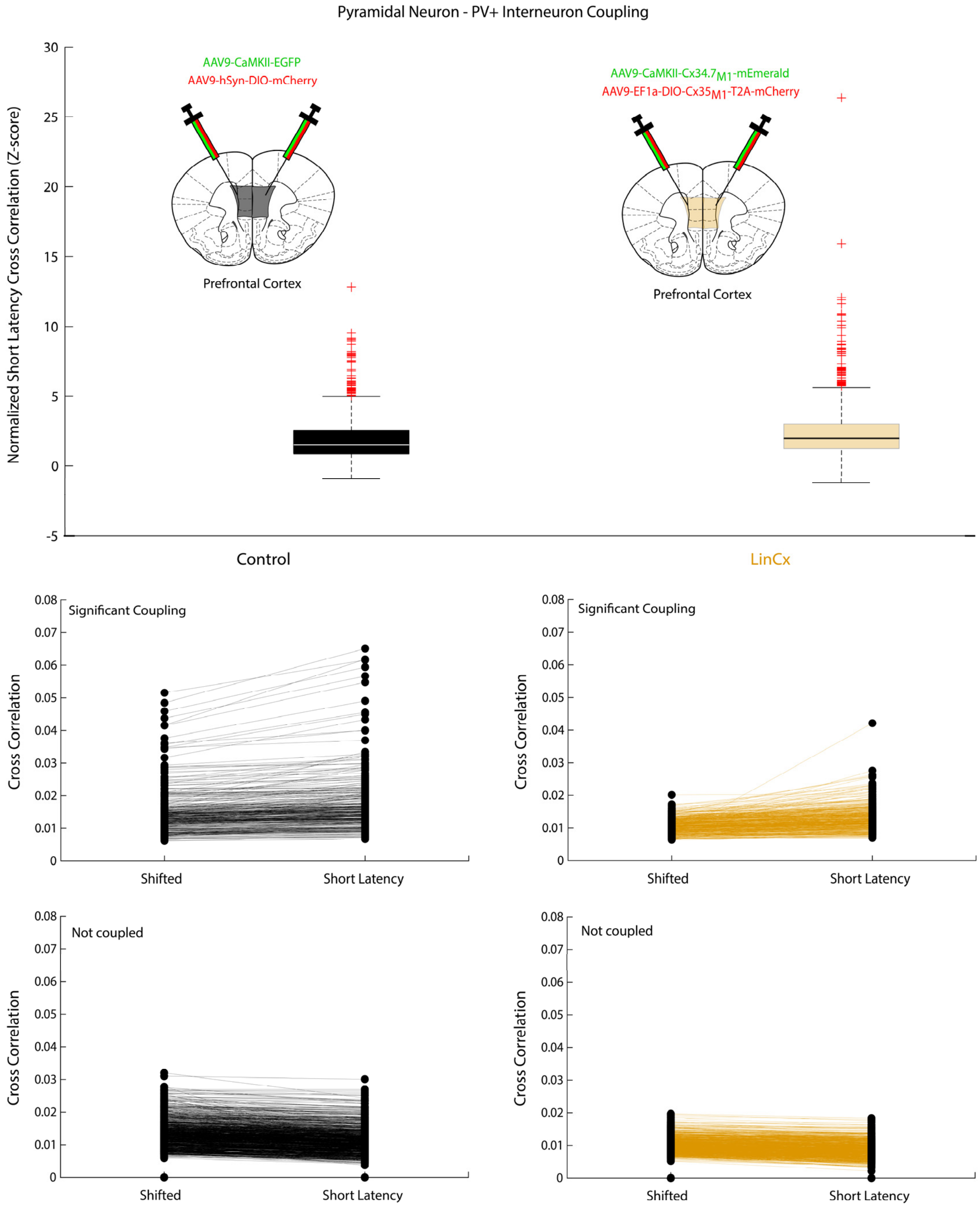


**Supplemental Figure S2: Coupling of PV+ interneurons with pyramidal neurons in Medial Prefrontal Cortex (mPFC).** Top row: Short latency cross-correlation values are shown for each PYR neuron -PV+ interneuron pair. The maximum short latency values were normalized to the shifted values for each pair using a z-scale for LinCx-expressing mice (right) and control mice (left). Middle and bottom rows: The 98.75 percentile for the shifted cross-correlation values and the maximum short latency cross-correlation values are shown for each neuron. All significantly coupled pairs are in the middle row, and the non-coupled pairs are in the bottom row, for control mice (left, black) and mice that expressed $Cx34.7_{M1}$/$Cx35_{M1}$ (right, tan). Brain slice images were adapted from Paxinos and Franklin[2].

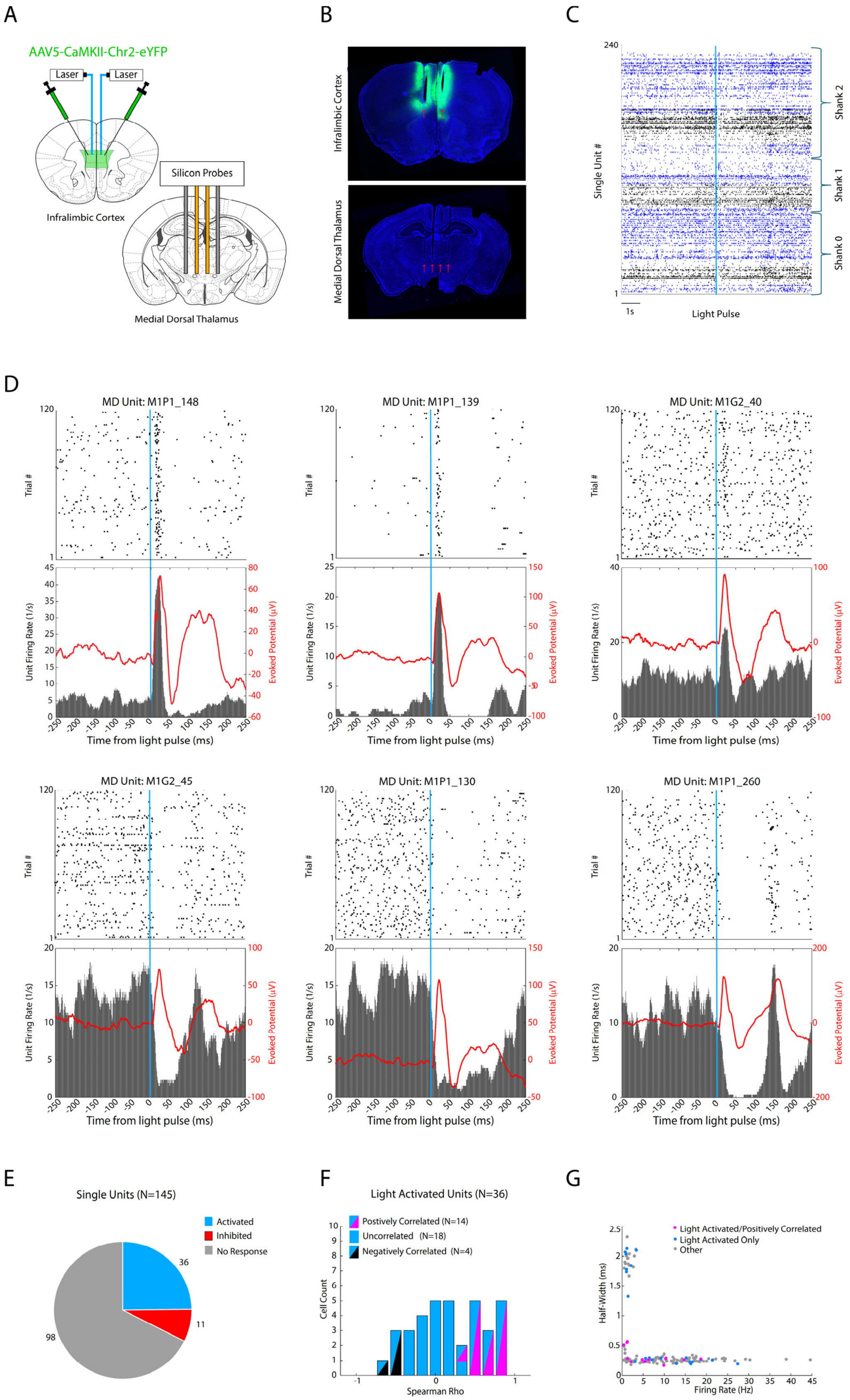
A
AAV5-CaMKII-Chr2-eYFP
Laser
Laser
Silicon Probes
Infralimbic Cortex
Medial Dorsal Thalamus
B
Infralimbic Cortex
Medial Dorsal Thalamus
C
Single Unit #
Shank 2
Shank 1
Shank 0
1s
Light Pulse
D
MD Unit: M1P1_148
MD Unit: M1P1_139
MD Unit: M1G2_40
MD Unit: M1G2_45
MD Unit: M1P1_130
MD Unit: M1P1_260
Trial #
Unit Firing Rate (1/s)
Evoked Potential (μV)
Time from light pulse (ms)
E
Single Units (N=145)
Activated
Inhibited
No Response
36
11
98
F
Light Activated Units (N=36)
Postively Correlated (N=14)
Uncorrelated (N=18)
Negatively Correlated (N=4)
Cell Count
Spearman Rho
G
Light Activated/Positively Correlated
Light Activated Only
Other
Half-Width (ms)
Firing Rate (Hz)

**Supplemental Figure S3: Medial dorsal thalamus (MD) cellular and local field potential responses to optogenetic stimulation of infralimbic cortex (IL). A)** Viral and electrical recording targeting approach. Two mice were infected with AAV5-ChR2-GFP in IL and implanted with a stimulating fiber in IL (bilaterally) and a 1024-channel silicon probe targeting MD. Activity was recorded from the two medial probes during quiet waking while mice were stimulated with blue light (1mW, 10ms pulse width, 493nm) 120 times with a pseudorandomized intertrial interval ranging from 8-24 seconds. **B)** Histological images showing ChR2-EYFP expression and optic fiber tracks in IL (red arrows, top), and individual electrode shank tracks in MD (red arrows, bottom). **C)** Raster plot showing 240 single units recorded concurrently from three implanted shanks that traversed MD. Cellular firing is shown relative to a light pulse in IL (light blue vertical line), and units from MD neurons are depicted in black. **D)** Examples of single units recorded from MD that showed a change in their activity following a light pulse. A raster plot depicting firing of each neuron relative to the 120 light pulses is shown on top. The average firing rate across all the trials is shown on the bottom for each neuron, and the mean LFP activity recorded from the same electrode channel is overlaid in red. Note that the three neurons in the top row showed an increase in their activity following a light pulse, while the three in the bottom row showed a decrease in their activity. **E)** Light stimulation increased the activity of 36 out of 145 single neurons isolated from MD and inhibited the activity of 11 MD neurons. **F)** Activity of the 36 MD neurons that were induced by light stimulation was compared to the evoked potential measured in the same channel. 14/36 of these neurons showed activity that was directly correlated with the evoked potential, confirming its local relevance. 4/36 neurons showed activity that was inversely correlated with the evoked potential. **G)** Firing rate and waveform properties for the 145 MD neurons. The 36 neurons that were activated by light stimulation are highlighted in pink (positively correlated with the evoked potential), or blue (not positively correlated with the evoked potential). The remaining neurons in gray were either suppressed or not modulated by light. Brain slice images were adapted from Paxinos and Franklin[2].

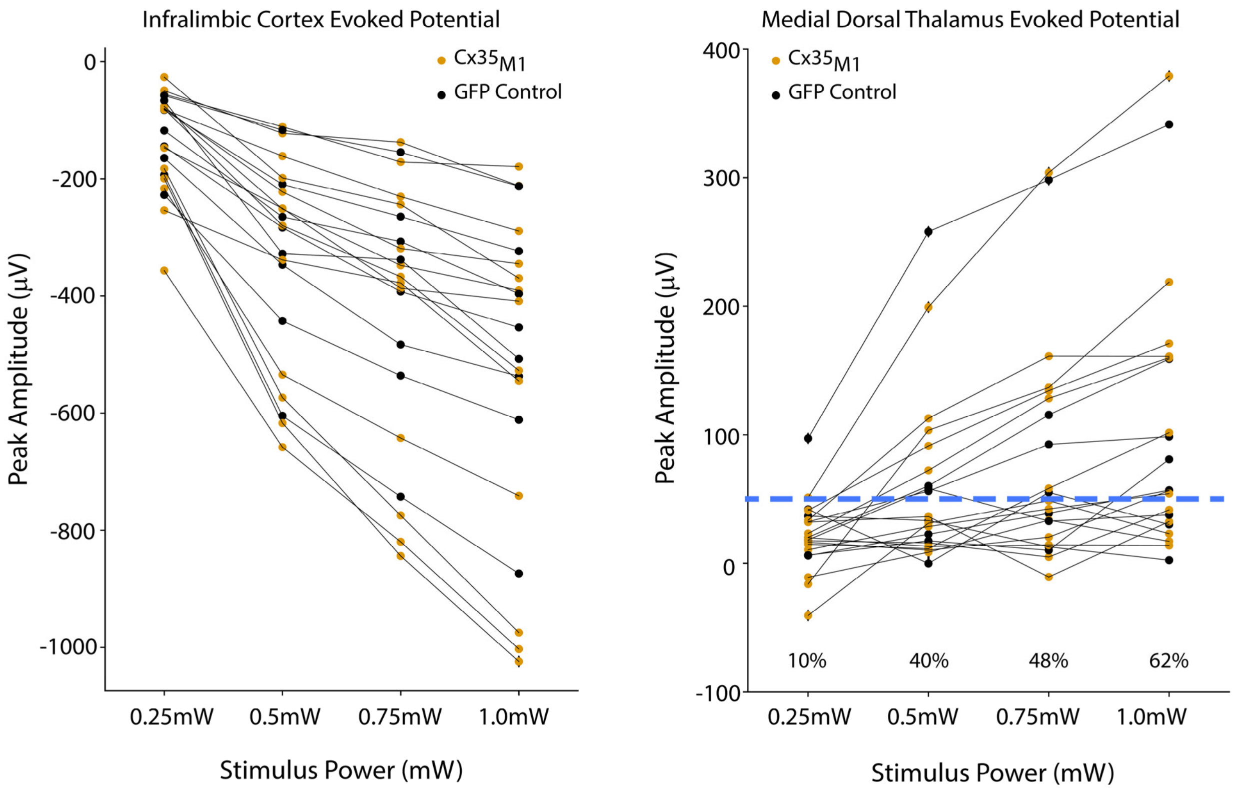


**Supplemental Figure S4: Cortical and thalamic potentials evoked by IL stimulation during first experimental session.** All responses were averaged across microwires. Left: Peak cortical amplitudes within 10ms of stimulation. Right: Peak thalamic amplitudes within 25ms of stimulation. The percentage of mice that showed thalamic evoked responses above 50mV (indicated by dashed blue line) is shown for each light intensity. Each line represents one mouse; N=21 total mice.

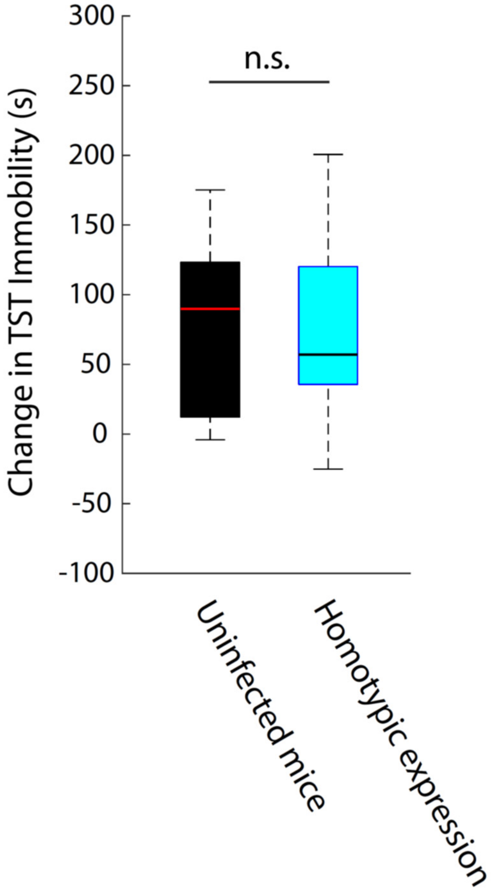


**Supplemental Figure S5: Homotypic expression of engineered gap junction hemichannels does not impact behavior.** Homotypic expression of $Cx34.7_{M1}$ or $Cx35_{M1}$ across the IL→MD circuit does not impact stress-induced behavioral adaptation in the tail suspension test (TST) relative to uninfected BALB/cJ mice ($T_{29}$=0.16, P=0.87, two-tailed unpaired t-test).

## Supplementary Reference


1 Hawk, J. D. *et al.* Integration of Plasticity Mechanisms within a Single Sensory Neuron of C. elegans Actuates a Memory. *Neuron* **97**, 356-367 e354 (2018). https://doi.org/10.1016/j.neuron.2017.12.027

2 Paxinos, G. & Franklin, K. B. J. *The mouse brain in stereotaxic coordinates*. Second edn, (Academic Press, 2001).
