# Supplementary material for: Long-term editing of brain circuits using an engineered electrical synapse
Source: Nature. 2026 May 13;655(8123):703–15. doi: 10.1038/s41586-026-10501-y (PMC13372691; doi:10.1038/s41586-026-10501-y)
Supplement: Supplementary file 2 — Reporting Summary [file 41586_2026_10501_MOESM2_ESM.pdf]

nature portfolio

Corresponding author(s): Dzirasa

Last updated by author(s): Mar 6, 2026

# Reporting Summary

Nature Portfolio wishes to improve the reproducibility of the work that we publish. This form provides structure for consistency and transparency in reporting. For further information on Nature Portfolio policies, see our Editorial Policies and the Editorial Policy Checklist.

## Statistics

For all statistical analyses, confirm that the following items are present in the figure legend, table legend, main text, or Methods section.

| n/a | Confirmed | |
|---|---|---|
| ☐ | ☒ | The exact sample size (*n*) for each experimental group/condition, given as a discrete number and unit of measurement |
| ☐ | ☒ | A statement on whether measurements were taken from distinct samples or whether the same sample was measured repeatedly |
| ☐ | ☒ | The statistical test(s) used AND whether they are one- or two-sided<br>*Only common tests should be described solely by name; describe more complex techniques in the Methods section.* |
| ☐ | ☒ | A description of all covariates tested |
| ☐ | ☒ | A description of any assumptions or corrections, such as tests of normality and adjustment for multiple comparisons |
| ☐ | ☒ | A full description of the statistical parameters including central tendency (e.g. means) or other basic estimates (e.g. regression coefficient) AND variation (e.g. standard deviation) or associated estimates of uncertainty (e.g. confidence intervals) |
| ☐ | ☒ | For null hypothesis testing, the test statistic (e.g. *F*, *t*, *r*) with confidence intervals, effect sizes, degrees of freedom and *P* value noted<br>*Give P values as exact values whenever suitable.* |
| ☒ | ☐ | For Bayesian analysis, information on the choice of priors and Markov chain Monte Carlo settings |
| ☒ | ☐ | For hierarchical and complex designs, identification of the appropriate level for tests and full reporting of outcomes |
| ☐ | ☒ | Estimates of effect sizes (e.g. Cohen's *d*, Pearson's *r*), indicating how they were calculated |

*Our web collection on statistics for biologists contains articles on many of the points above.*

## Software and code

Policy information about availability of computer code

| | |
|---|---|
| Data collection | Flow cytometry data were acquired using a BD FACSCanto II cytometer and BD FACSDiva software (BD Biosciences). Confocal imaging was performed on Nikon Ti2-E CSU-W1 and Leica SP5 systems, with image acquisition using NIS-Elements (Nikon). Calcium imaging in C. elegans was conducted using microscopes controlled by µManager (open-source). Electrophysiological recordings were obtained using a Scientifica upright microscope with data filtered using Clampfit 11.4 (Molecular Devices). For molecular dynamics studies, structural models were generated using Robetta, embedded into membrane systems via CHARMM-GUI, visualized with VMD, and simulated using NAMD. Plasmid constructs were designed and annotated using ApE (A Plasmid Editor). |
| Data analysis | Flow cytometry data were processed using a custom Python-based pipeline (FETCH), available at https://github.com/carlson-lab/FETCH, which includes kernel density estimation-based gating, quadrant classification, and normalization. Imaging data were analyzed in Fiji/ImageJ (open-source), including use of the "Colocalization Threshold" plugin for quantifying overlap of fluorescent signals. Molecular dynamics stimulation was assisted with a custom Python package available at https://github.com/carlson-lab/VMD-and-NAMD-Connexin-Protein-Simulation-Protocol. Murine circuit interogation data were analyzed using custom Python scripts available at https://github.com/carlson-lab/OptoLinCx. HEK293 cell electrophysiological traces were analyzed by plotting current–voltage relationships and applying filtering and quantification in Clampfit. Neurophysiological and statistical analyses were performed using MATLAB (Versions 2021A-2024B, The MathWorks, Inc., Natick, MA). All analyses used standard frequentist statistical approaches; no Bayesian or hierarchical models were applied. |

For manuscripts utilizing custom algorithms or software that are central to the research but not yet described in published literature, software must be made available to editors and reviewers. We strongly encourage code deposition in a community repository (e.g. GitHub). See the Nature Portfolio guidelines for submitting code & software for further information.

# Data

Policy information about availability of data

All manuscripts must include a data availability statement. This statement should provide the following information, where applicable:

- Accession codes, unique identifiers, or web links for publicly available datasets
- A description of any restrictions on data availability
- For clinical datasets or third party data, please ensure that the statement adheres to our policy

All data generated in support of the findings of this study are available from the corresponding author for academic purposes upon reasonable request. Such data will be made available under a material transfer agreement. Connexin gene information was procured from the National Center for Biotechnology Information and the Ensembl genome browser. These datasets can be readily accessed ncbi.nlm.nih.gov and ensembl.org.

# Research involving human participants, their data, or biological material

Policy information about studies with human participants or human data. See also policy information about sex, gender (identity/presentation), and sexual orientation and race, ethnicity and racism.

| | |
|---|---|
| Reporting on sex and gender | N/A |
| Reporting on race, ethnicity, or other socially relevant groupings | N/A |
| Population characteristics | N/A |
| Recruitment | N/A |
| Ethics oversight | N/A |

Note that full information on the approval of the study protocol must also be provided in the manuscript.

# Field-specific reporting

Please select the one below that is the best fit for your research. If you are not sure, read the appropriate sections before making your selection.

☒ Life sciences ☐ Behavioural & social sciences ☐ Ecological, evolutionary & environmental sciences

For a reference copy of the document with all sections, see nature.com/documents/nr-reporting-summary-flat.pdf

# Life sciences study design

All studies must disclose on these points even when the disclosure is negative.

**Sample size**

No formal statistical methods were used to pre-determine sample sizes. Instead, sample sizes were chosen based on standards in the field and our prior studies using similar assays (e.g., FETCH, calcium imaging, electrophysiology, and behavior). For most FETCH experiments, 5–6 samples were used per experimental condition. We also measured the scores for two large populations of non-docking pairs (54 and 92 pairs).which we used screen the effect of mutating amino acid residues on Cx34.7 and Cx35. Our preliminary analysis demonstrated that these sample sizes provided sufficient power to determine the impact of amino acid substitutions on docking. The sample size for our oocyte experiments was based on our prior work probing gap junction function using this system. The sample size for our in vivo electrophysiological studies using HEK293FT cells was based on our preliminary experiments in this system. Our sample sizes for the c. elegans studies were informed by our prior work using Cx36 to modify the same circuitry evaluated in this manuscript (Hawk et al, Neuron 2018). For mouse in vivo physiology experiments designed to evaluate LFP actvity, we set out to use group sizes of 7-14 mice. This sample size was based on our prior work quantifying cross frequency coupling in hippocampus (Dzirasa et al, Journal of Neuroscience, 2009) and prefrontal cortex (Dzirasa, Coque et al, Journal of Neuroscience, 2011), and optogentically evoked potentials (Kumar et al, Journal of Neuroscience 2011). For mouse behavioral experiments, we used group sizes of 7-14 for most experiments. This sample size was based on our prior work quantifying stress (Carlson et al, Biological Psychiatry, 2017) and social behavior (Mague et al, Neuron 2022) across groups. For in vivo electrophysiological experiments at the cellular level, sample sizes reflect the number of successfully recorded neurons (e.g., 91 vs. 101 PV+ neurons), which were sufficient to detect group differences using standard statistical tests. In all cases, sample sizes and statistical tests are detailed in figure legends and methods.

**Data exclusions**

Data were excluded in several contexts based on pre-established, method-specific quality control criteria. For flow cytometry analyses using the FETCH pipeline, samples were excluded automatically if fewer than 500 gated single cells were detected, if fluorescent expression was poor, or if quadrant asymmetry suggested unreliable transfection (e.g., >2-fold imbalance in red vs. green single-positive cells); such samples were flagged as “dubious” and excluded from downstream quantification. In electrophysiological experiments evaluating optogenetically evoked potentials, individual trials were excluded if >50% of their time points were identified as outliers based on a 1.5 interquartile range (IQR) rule, and channels were excluded if baseline activity exceeded 25 µV. Additionally, mice were excluded from these analyses if they failed to exhibit evoked responses to 3 mW stimulation in the MD region, indicating sub-threshold responsiveness for meaningful quantification. Finally mice were removed if histological analysis failed to confirm viral expression or appropriate electrode targeting. These criteria were

established a priori based on expected physiological responses and pilot data.

No other explicit data exclusions were reported for behavioral, imaging, or in vitro electrophysiology experiments. All exclusions are described in the Methods and Supplementary Figure legends where applicable.

| | |
|---|---|
| Replication | All key findings reported in the study were performed using biological replicates. Each major assay—FETCH docking, electrophysiological measurements, calcium imaging in C. elegans, and behavioral assessments in mice—was repeated across multiple biological replicates and/or independent experimental cohorts. For in vitro experiments, results were replicated across at least three independent transfections or culture preparations. Behavioral findings in C. elegans and mice were confirmed across distinct genetic lines or independently injected cohorts, respectively. Electrophysiological recordings were repeated across multiple cells or animals and consistently showed similar trends. For in vivo experiments in mice, physiological and behavioral measures were taked from different groups of animals to ensure that any convergence of findings were not due to the overlap biological samples. The effect size for each of these experiments is provided in the manuscript. Criteria for assay inclusion and quality control (e.g., flow cytometry gating thresholds, electrophysiological trial exclusion, behavioral responsiveness) were pre-established and applied consistently. All attempts at replication were successful. |
| Randomization | Samples and animals were assigned to experimental groups based on experimental design rather than randomization. In vitro assays, including FETCH docking and electrophysiological analyses, were structured around defined pairing of connexin-expressing cell populations, where group assignment was dictated by specific Cx combinations. In C. elegans experiments, animals were grouped by genotype or transgene identity. In mouse studies, animals were assigned to groups based on viral injection protocols (e.g., expression of LinCx vs. fluorescent controls) and were sex- and age-matched across groups where applicable.<br><br>While randomization was not formally applied, allocation procedures ensured that experimental and control groups were balanced for potential confounding factors (e.g., age, sex, surgical timing). Covariates such as fluorescent expression quality, baseline behavior, or physiological response thresholds were either matched across groups or controlled through inclusion/exclusion criteria and statistical modeling where appropriate. Full details of group structure and sample matching are described in the Methods and figure legends. |
| Blinding | Blinding was applied to behavioral experiments and video analyses. Specifically, all tail suspension and open field behavioral data were recorded and analyzed using Ethovision XT software by investigators blinded to group identity. For other experiments (e.g., flow cytometry, imaging, electrophysiology), blinding was not routinely applied due to the nature of the experimental design, where sample identity (e.g., connexin isoform or mutant construct) was essential for downstream pairing and analysis. In these cases, potential biases were mitigated through predefined analysis pipelines, automated gating or quantification scripts (e.g., in FETCH or electrophysiology), and consistent quality control thresholds applied across all conditions. Full details are provided in the Methods section. |

# Reporting for specific materials, systems and methods

We require information from authors about some types of materials, experimental systems and methods used in many studies. Here, indicate whether each material, system or method listed is relevant to your study. If you are not sure if a list item applies to your research, read the appropriate section before selecting a response.

## Materials & experimental systems

| n/a | Involved in the study | |
|---|---|---|
| ☐ | ☒ | Antibodies |
| ☐ | ☒ | Eukaryotic cell lines |
| ☒ | ☐ | Palaeontology and archaeology |
| ☐ | ☒ | Animals and other organisms |
| ☒ | ☐ | Clinical data |
| ☒ | ☐ | Dual use research of concern |
| ☒ | ☐ | Plants |

## Methods

| n/a | Involved in the study | |
|---|---|---|
| ☒ | ☐ | ChIP-seq |
| ☐ | ☒ | Flow cytometry |
| ☒ | ☐ | MRI-based neuroimaging |

## Antibodies

| | |
|---|---|
| Antibodies used | All antibodies used in the study were commercially sourced and validated for western blotting. Anti-Cx43 (Cell Signaling #3512, 1:1000), anti-Cx45 (Abcam #ab316742, 1:1000), and anti-GAPDH (Abcam #ab181602, 1:5000) were used to validate knockout and expression in HEK293FT cell lines. HRP-conjugated anti-rabbit secondary antibody (Cell Signaling #7074) was used for detection. Lot numbers were not recorded. No antibodies were used for immunostaining or immunohistochemistry |
| Validation | Both primary antibodies used in this study were commercially sourced and selected based on extensive prior validation and community use. The anti-Cx43 antibody (Cell Signaling Technology, cat. #3512) is a rabbit polyclonal antibody validated for western blotting in human, mouse, and rat samples. According to the manufacturer and third-party databases (e.g., CiteAb), this antibody has been cited in over 300 peer-reviewed publications and has been independently validated in Cx43 knockout models, supporting its specificity for endogenous protein detection in mammalian lysates (CiteAb – Cx43 #3512).<br><br>The anti-Cx45 antibody (Abcam, cat. #ab316742) is a rabbit polyclonal antibody validated for western blot applications, with documented reactivity in human samples. While fewer citations are listed compared to Cx43, this antibody has been used in published studies for protein quantification and western blot analysis of connexin isoforms, and is included in Abcam's validation for use in lysate-based assays. No additional in-house validation was performed for either antibody beyond their use in western blotting for knockout verification of connexin-deficient HEK293FT cells. |

## Eukaryotic cell lines

Policy information about cell lines and Sex and Gender in Research

**Cell line source(s)**

HEK293FT cells were purchased from Thermo Fisher Scientific (Cat# R70007) and maintained under standard tissue culture conditions. These cells are a commercially available human embryonic kidney cell line with a female origin, though sex was not explicitly confirmed or varied in this study
. Cx43 and Cx45 double-knockout HEK293FT cell lines were generated via CRISPR-Cas9 editing by the Duke Functional Genomics Core, using clonal isolation and validation via sequencing and western blotting.

Xenopus laevis oocytes used for dual voltage clamp recordings were harvested from adult female frogs, the standard source for oocyte-based expression systems. Oocytes were allocated from individual frogs to specific experimental conditions to reduce inter-animal variability (e.g., one frog per connexin condition)

**Authentication**

HEK293FT cells (Thermo Fisher Scientific, Cat# R70007) were obtained directly from the supplier and maintained under standard culture conditions. These cells were not further authenticated in-house. The Cx43/Cx45 double-knockout HEK293FT line was generated using CRISPR-Cas9 and validated functionally through genotyping PCR and western blot analysis confirming the absence of Cx43 and Cx45 protein expression.

**Mycoplasma contamination**

No mycoplasma contamination testing was performed on the HEK293FT or Cx43/Cx45 double knockout HEK293FT cell lines during the course of this study. Cells were maintained under sterile conditions and used at low passage, but sterility was not confirmed through dedicated mycoplasma assays.

**Commonly misidentified lines**
(See ICLAC register)

HEK293FT cells were used in this study and are listed by ICLAC as commonly misidentified. These cells originate from a transformed human embryonic kidney cell line but have undergone significant genetic drift and contain adenoviral sequences. Despite this, they are widely accepted for transfection-based assays and were selected in this study for their high transfection efficiency, mammalian expression compatibility, and suitability for gap junction formation and fluorescence-based analysis. No other commonly misidentified cell lines were used.

## Animals and other research organisms

Policy information about studies involving animals; ARRIVE guidelines recommended for reporting animal research, and Sex and Gender in Research

**Laboratory animals**

Mice were of the Mus musculus species and included several strains: All mice were housed 3-5/cage on a 12-hour light/dark cycle and maintained in a humidity (30-70%)- and temperature (73±5 °F)-controlled room with water and food available ad libitum.

C57BL/6J (Jackson Laboratory, Stock No. 000664), PV-Cre (B6.129P2-Pvalbtm1(cre)Arbr/J, Stock No. 017320), VGLUT2-flp (B6;129S-Slc17a6tm1.1(flpo)Hze/J, Stock No. 030212), and BALB/cJ (Stock No. 000651). Mice were between 2.5 to 5 months old at the time of viral surgeries and electrophysiological recordings, with behavioral testing conducted in PV-Cre/VGLUT2-flp mice at 12–13 weeks of age, and IL–MD circuit experiments conducted in 3-month-old BALB/cJ males. Both male and female mice were used in the study, with groups generally sex-balanced unless otherwise noted.

For C. elegans experiments, wild-type N2 hermaphrodites and a range of genetically modified strains (e.g., DCR3056, DCR5793, DCR8678) were used. All C. elegans animals were hermaphrodites, which is standard for behavioral and neuronal imaging studies. Strains were obtained from the Caenorhabditis Genetics Center or generated in-house, and all were maintained under standard laboratory conditions.

**Wild animals**

This study did not involve any wild animals. All animal experiments were conducted using laboratory strains of mice and C. elegans obtained from established repositories.

**Reporting on sex**

Both male and female mice were used in the majority of experiments, including viral injections, electrophysiology, and behavioral studies. For example, PV-Cre and VGLUT2-flp mice were bred and used in sex-balanced groups (N=28 total injected mice), and the study explicitly states that viral manipulations were balanced by sex in multiple experiments
. In contrast, 3-month-old male BALB/cJ mice were used exclusively for the infralimbic cortex–medial dorsal thalamus (IL–MD) circuit editing experiments to mirror prior studies in that circuit.

In the analysis of social preference and open field exploration, the mice were 12–13 weeks old and balanced by sex, though the data are not reported disaggregated by sex in the figures or results text.No formal sex-based statistical comparisons were reported in the manuscript. The rationale for this is that no sex differences were hypothesized, and sample sizes within each sex may have been underpowered for sex-specific analysis.

For C. elegans experiments, only hermaphrodites were used, which is standard for behavioral and neural imaging studies in this species. The sex of cultured HEK293FT cell lines was not varied or considered relevant to the experimental design.

**Field-collected samples**

This study did not involve any samples collected from the field. All experimental organisms and materials were obtained from laboratory-maintained strains, established repositories, or commercial suppliers.

**Ethics oversight**

All vertebrate animal experiments were conducted with protocols approved by the Duke University Institutional Animal Care and Use Committee (IACUC) and were carried out in accordance with the National Institutes of Health (NIH) guidelines for the Care and Use of Laboratory Animals. This oversight applied to all studies involving mice, including viral surgeries, electrophysiology, and behavioral

assays. No additional ethical approval was required for C. elegans studies, as these nematodes are not classified as vertebrate animals and are exempt from IACUC regulation under current U.S. federal guidelines.

Note that full information on the approval of the study protocol must also be provided in the manuscript.

## Plants

Seed stocks

*Report on the source of all seed stocks or other plant material used. If applicable, state the seed stock centre and catalogue number. If plant specimens were collected from the field, describe the collection location, date and sampling procedures.*

Novel plant genotypes

*Describe the methods by which all novel plant genotypes were produced. This includes those generated by transgenic approaches, gene editing, chemical/radiation-based mutagenesis and hybridization. For transgenic lines, describe the transformation method, the number of independent lines analyzed and the generation upon which experiments were performed. For gene-edited lines, describe the editor used, the endogenous sequence targeted for editing, the targeting guide RNA sequence (if applicable) and how the editor was applied.*

Authentication

*Describe any authentication procedures for each seed stock used or novel genotype generated. Describe any experiments used to assess the effect of a mutation and, where applicable, how potential secondary effects (e.g. second site T-DNA insertions, mosiacism, off-target gene editing) were examined.*

## Flow Cytometry

### Plots

Confirm that:

☒ The axis labels state the marker and fluorochrome used (e.g. CD4-FITC).

☒ The axis scales are clearly visible. Include numbers along axes only for bottom left plot of group (a 'group' is an analysis of identical markers).

☒ All plots are contour plots with outliers or pseudocolor plots.

☒ A numerical value for number of cells or percentage (with statistics) is provided.

### Methodology

Sample preparation

All flow cytometry experiments were conducted in wild-type HEK293FT cells (Thermo Fisher Scientific, Cat# R70007). Cells were seeded on 10 µg/mL fibronectin-coated multi-well plates and transfected with fluorescently tagged connexin constructs using polyethyleneimine (PEI) in Opti-MEM medium. Following transfection, the media was replaced after 16–18 hours to reduce toxicity, and cells were allowed to express the constructs for an additional 24–48 hours.

For the FETCH assay, separately transfected populations of HEK293FT cells expressing different connexin-fluorophore combinations were trypsinized, combined into a single well (to induce cell–cell contact), and plated at high density on fibronectin-coated plates to promote gap junction formation. These co-plated cells were incubated for ~20–24 hours to allow for internalization of fluorescently labeled gap junctions.

After incubation, cells were again trypsinized, resuspended in PBS containing 10 U/mL DNase, and fixed with 1.5% paraformaldehyde. Final volumes for flow cytometry were ~150 µL for samples from 96-well plates and ~600 µL for samples from 24-well plates.

Instrument

All flow cytometry data were acquired using a BD FACSCanto II cytometer (Becton Dickinson) equipped with 488 nm and 633 nm lasers and analyzed using BD FACSDiva software.

Software

Flow cytometry data were collected using BD FACSDiva software (Becton Dickinson), which controls the BD FACSCanto II cytometer and manages .fcs file export. Data were analyzed using a custom Python-based pipeline developed specifically for the FETCH assay. This analysis pipeline includes modules for kernel density estimation (KDE)-based gating, standard deviation filtering, quadrant classification of dual-fluorescence, and normalization of fluorescence intensity.

The FETCH analysis pipeline has been made publicly available on GitHub at:
https://github.com/carlson-lab/FETCH

This repository contains all scripts necessary for batch processing of .fcs files, along with documentation and example data.

Cell population abundance

The FETCH assay is based on analyzing co-plated, separately transfected HEK293FT populations without physical sorting; instead, cell populations are assessed by flow cytometry based on fluorescent protein expression. After co-plating, dual-labeled cells (those positive for both fluorescent constructs) represent the fraction of cells that have internalized gap junctional material from both populations. Purity and abundance of relevant populations were quantified directly during analysis by gating for single cells and measuring the proportion of cells falling into each fluorescence quadrant (single positive for each fluorophore, double positive, and double negative).

Purity was determined by applying strict gating thresholds using kernel density estimation and standard deviation filters within the custom FETCH analysis pipeline. Only events falling within the main population of single cells (as assessed by forward and side scatter) and passing the automated fluorescence quality filters were included in the analysis. Samples were excluded if there was a >2-fold imbalance between red and green single-positive populations or if fewer than 500 single cells were detected, ensuring that the double-positive (dual-labeled) fraction was measured in high-quality, well-mixed

populations.

This approach allowed for accurate determination of the abundance and purity of dual-labeled cells (indicative of gap junction transfer) in each experimental condition. No additional post-sort purification was performed, as the assay quantifies cellular mixing and transfer in situ following co-culture and fixation.

Gating strategy

Flow cytometry data were analyzed using a custom Python-based FETCH pipeline. Initial gating was performed on forward scatter (FSC) and side scatter (SSC) to isolate single, intact HEK293FT cells and exclude debris, dead cells, and aggregates.

Fluorescence thresholds for red (mCherry) and green (mEmerald) channels were determined using kernel density estimation (KDE) to identify inflection points separating negative and positive populations. A standard deviation filter was also applied to refine quadrant boundaries.

Cells were classified into four fluorescence quadrants:
Q1: Red-only (mCherry$^{+}$ / mEmerald$^{-}$)
Q2: Dual-labeled (mCherry$^{+}$ / mEmerald$^{+}$) — indicates gap junction-mediated exchange
Q3: Green-only (mEmerald$^{+}$ / mCherry$^{-}$)
Q4: Double-negative (mCherry$^{-}$ / mEmerald$^{-}$)

Samples were excluded if fewer than 500 gated single cells were detected or if there was >2-fold imbalance between single-positive quadrants (Q1 vs. Q3). All gating and quadrant classification were performed automatically within the FETCH pipeline (https://github.com/carlson-lab/FETCH) to ensure consistency and objectivity.

☒ Tick this box to confirm that a figure exemplifying the gating strategy is provided in the Supplementary Information.
